# A comprehensive checklist of vascular epiphytes of the Atlantic Forest reveals outstanding endemic rates

**DOI:** 10.3897/phytokeys.58.5643

**Published:** 2016-01-12

**Authors:** Leandro Freitas, Alexandre Salino, Luiz Menini Neto, Thaís Elias Almeida, Sara Ribeiro Mortara, João Renato Stehmann, André Marcio Amorim, Elsie Franklin Guimarães, Marcus Nadruz Coelho, Ana Zanin, Rafaela Campostrini Forzza

**Affiliations:** 1 Jardim Botânico do Rio de Janeiro; 2 Universidade Federal de Minas Gerais; 3 Centro de Ensino Superior de Juiz de Fora; 4 Universidade Federal do Oeste do Pará; 5 Universidade de São Paulo; 6 Universidade Estadual de Santa Cruz e Herbário do Centro de Pesquisas do Cacau; 7 Universidade Federal de Santa Catarina

**Keywords:** Angiosperms, canopy, ferns, lycophytes, hotspots, life-forms, monocots, tropical forests

## Abstract

Knowledge of the geographic distribution of plants is essential to underpin the understanding of global biodiversity patterns. Vascular epiphytes are important components of diversity and functionality of Neotropical forests but, unlike their terrestrial counterparts, they are under-represented in large-scale diversity and biogeographic analyses. This is the case for the Atlantic Forest - one of the most diverse and threatened biomes worldwide. We provide the first comprehensive species list of Atlantic Forest vascular epiphytes; their endemism patterns and threatened species occurrence have also been analyzed. A list with 2,256 species of (hemi-)epiphytes - distributed in 240 genera and 33 families - is presented based on the updated Brazilian Flora Checklist. This represents more than 15% of the total vascular plant richness in the Atlantic Forest. Moreover, 256 species are included on the Brazilian Red List. More than 93% of the overall richness is concentrated in ten families, with 73% represented by Orchidaceae and Bromeliaceae species alone. A total of 78% of epiphytic species are endemic to the Atlantic Forest, in contrast to overall vascular plant endemism in this biome estimated at 57%. Among the non-endemics, 13% of epiphytic species also occur either in the Amazon or in the Cerrado - the other two largest biomes of Brazil – and only 8% are found in two or more Brazilian biomes. This pattern of endemism, in addition to available dated phylogenies of some genera, indicate the dominance of recent radiations of epiphytic groups in the Atlantic Forest, showing that the majority of divergences dating from the Pliocene onwards are similar to those that were recently reported for other Neotropical plants.

## Introduction

Geographic distribution of vascular plant species forms the framework to understand terrestrial diversity patterns, their relationship to environmental and historical factors and the ecological and evolutionary mechanisms underlying these patterns (Currie et al. 2004, Ricklefs 2004, Kreft and Jetz 2007).The Neotropical region harbours more species than any other place on Earth (Gentry 1982). However, the scarcity of distributional data has hampered progress towards developing more general models for the region (Kamino et al. 2012).Vascular epiphytes are known to be an important component of plant diversity in Neotropical forests, although less is understood about their ecology and phytogeography than about their terrestrial counterparts (Kreftet al. 2004). This lack of knowledge of vascular epiphytes stems from logistical constraints associated with the collection of samples (Barker and Pinard 2001, Burns and Zotz 2010). Whether richness and endemism patterns of Neotropical ecosystems are similar for epiphytes and terrestrial plants remains an underexplored question (see Fontoura et al. 2012).

The high abundance of vascular epiphytes constitutes a remarkable characteristic of tropical forests. These plants may exceed 50% of local vascular plant richness in some montane forests (Kelly et al. 2004) and as many as 126 species have been found on a single tree (Schuettpelz and Trapnell 2006, see also Krömer et al. 2005). The importance of epiphytic plants functional role in forest communities cannot be underestimated, as they influence nutrient cycles and provide shelter as well as nesting materials and food for animals (review in Bartels and Chen 2012). They can also enhance diversity, as is the case of the microcosms associated with bromeliad phytotelmata (Richardson 1999). Epiphytes represent 9–10% of the total known vascular flora, comprising between 23,000 and 29,000 species, distributed among 73–84 families (Kress 1986, Gentry and Dodson 1987, Zotz 2013) but most species belong to a few families (e.g., about 80% of the species belong to the Orchidaceae, Araceae, Bromeliaceae, Polypodiaceae and Piperaceae; Gentry and Dodson 1987). Although taxonomic distribution of epiphytism in vascular plants is well established, few studies explicitly address biogeographical aspects of epiphytes (reviewed in Kreft et al. 2004).

The Atlantic Forest, one of the floristic diversity centres in the Neotropics (Gentry 1982, Stehmann et al. 2009) used to be among the largest tropical forests of the Americas, originally covering around 150 million hectares. Unfortunately, only 12% of the original area still remains (Ribeiro et al. 2009). This biome occupies a narrow strip of land along the eastern coast of Brazil, from sea level towards the west, reaching the hinterland mountains (up to 3,000 m) and becoming broader between southeastern and southern Brazil. With a latitudinal range spanning from around 6° to 30°S, it spreads from tropical into subtropical regions, covering 13% of the Brazilian territory. Around 95% of this vast biome occurs in Brazil, extending only marginally into Argentina, Paraguay and Uruguay (Ribeiro et al. 2009, Stehmann et al. 2009). Altitudinal, latitudinal and longitudinal ranges create highly heterogeneous environmental conditions that lead to highly variable forest composition. While the coastal areas receive abundant rain all year-round, reaching more than 4,000 mm/year, inland forests receive as little as 1,000 mm/year, and this precipitation is distributed according to a seasonal pattern (Câmara 2003). Recent compilations regarding Brazilian floristic diversity recorded more than 16,000 species of vascular plants for the Atlantic Forest, approximately half of them being endemic to this biome (e.g., Stehmann et al. 2009, Forzza et al. 2012, List of Species of the Brazilian Flora 2014). These compilations offer important insights into the significance of the Atlantic Forest for Neotropical diversity and provide a basis for other studies related to biodiversity and conservation.

This paper provides the first comprehensive species list of vascular epiphytes for the Brazilian Atlantic Forest, identifying: (i) taxonomic representativeness, (ii) geographic distribution and endemism, and (iii) occurrence of threatened species among epiphytes of this biome. This study is the first approach to the analysis of epiphyte diversity patterns in the Neotropics involving the mechanisms underlying floristic diversity in tropical forests.

## Methods

Raw data for this study were obtained from the database developed by Stehmann et al. (2009). This list includes 14,552 vascular species from different vegetation types within the original extention of the Atlantic Forest, according to Brazilian legislation (Atlantic Forest law, nº. 11,428). Plant habit (hemi-epiphytic or epiphytic) was assigned by consulting labels from the Rio de Janeiro Botanical Garden Herbarium samples (RB, JABOT database, www.jbrj.gov.br), species descriptions in taxonomic literature, and information provided by specialists. Scientific names were updated according to the List of Species of the Brazilian Flora (2014), which listed 15,490 vascular species occurring in Atlantic Forest when last acessed.

All facultative and holoepiphytes, as well as all primary and secondary hemiepiphytes were listed, but accidental and heterotrophic epiphytes were excluded following the concept provided by Benzing (2000). The term epiphyte was used for both holoepiphytic and hemiepiphytic species throughout the text for convenience, with exceptions explicitly mentioned. Geographic distribution patterns were determined for the four major Brazilian biomes (Atlantic Forest, Amazon, Caatinga and Cerrado), based on the same criteria used in The Brazilian Catalogue of Plants and Fungi (Forzza et al. 2010, 2012). Data regarding conservation status were obtained from the Red Book of Brazilian Flora (Martinelli and Moraes 2013). The resulting list was checked for inconsistencies in life-form terminology and geographic distribution, and finally species and family richness and distribution patterns were compared (i.e., endemics to the Atlantic Forest and to Brazil; occurrence in the Atlantic Forest and in one other biome; and widespread species).

## Results

### Taxonomic diversity

Our compilation recorded 2,256 species of epiphytes distributed in 33 vascular plant families (Suppl. material 1). This corresponds to 15% of the vascular flora from the Atlantic Forest (Table 1). Almost one third of fern and lycophyte species from the Atlantic Forest are epiphytes (Table 1).

**Table 1.** Number of vascular taxa for the Brazilian Atlantic Forest. Compiled from the List of Species of the Brazilian Flora (2014).

	Taxonomic group	Families	Genera	Species
**All life-Forms**	Angiosperms	211	1,975	14,638
	Ferns and lycophytes	36	127	852
	Total - vascular plants	247	2,102	15,490
**(Hemi-) Epiphytes**	Angiosperms	22	197	2,013
	Ferns and lycophytes	11	43	243
	Total - vascular plants (epiphytes/all life-forms)	33 (13.4%)	240 (11.4%)	2,256 (15.4%)

**Figure 1.**
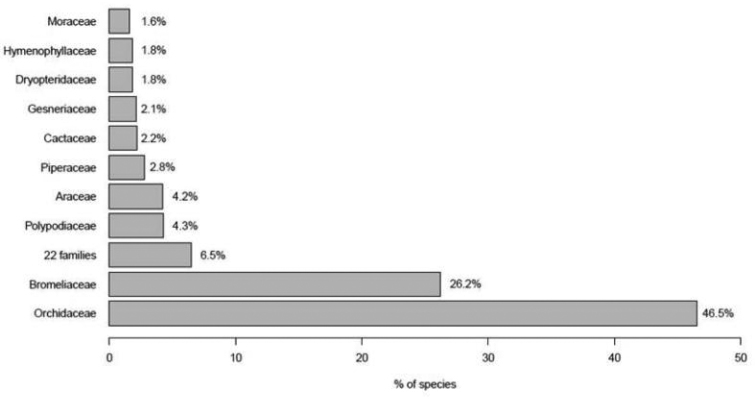
Family representativeness of Atlantic Forest vascular epiphyte species.

**Figure 2.**
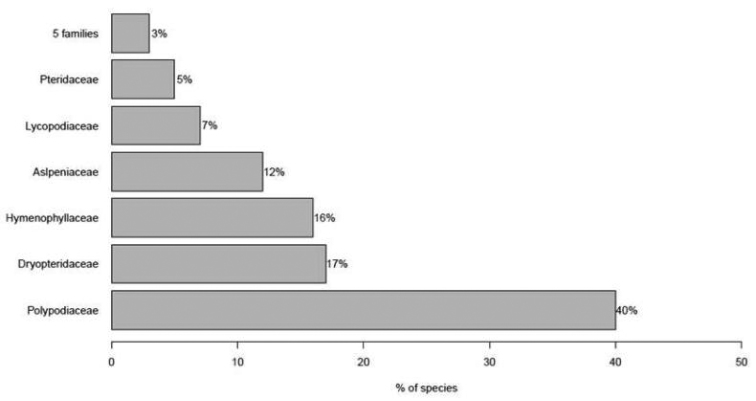
Family representativeness of Atlantic Forest fern and lycophyte epiphyte species.

Representation of vascular epiphytes is highly concentrated in a few families, with the ten richest families accounting for around 93% of epiphyte diversity (Fig. 1). Only nine Atlantic Forest families (five angiosperms and four fern families) are represented by only one or two epiphytic species (Suppl. material 1). Orchidaceae and Bromeliaceae comprised the richest families with almost 73% of vascular epiphyte species, while Polypodiaceae alone comprises more than 40% of fern and lycophyte epiphytic species (Fig. 2).

### Geographic distribution and endemism

About three out of four vascular epiphytic species from the Atlantic Forest are endemic to this biome (Table 2), including 21 genera, most of them belonging to Orchidaceae (11) and Bromeliaceae (5). In terms of overall endemism, 78% of the vascular epiphytes of the Atlantic Forest (1,761 species) are endemic to Brazil. Less than 8% of the Atlantic Forest epiphytes are widely distributed (i.e., occur in three or four Brazilian biomes). The remaining 13.5% are species that occur either in the Amazon or Cerrado in addition to the Atlantic Forest (Table 2). Atlantic Forest vascular epiphytes are unequally distributed among angiosperms and ferns and lycophytes, with 60% of the species belonging to the latter group being restricted to the Atlantic Forest, while more than 14% are widely distributed. Moreover, disjunct distribution between Atlantic Forest and the Amazon is higher among ferns and lycophytes (Table 2).

**Table 2.** Distribution patterns of vascular (hemi-)epiphytes for Atlantic Forest along the four main Brazilian biomes, and the number of endangered species.

	**Number and percentage of species**		
	**Angiosperms**	**Ferns and lycophytes**	**Vascular flora**
**Restricted to Atlantic Forest**	1,595 (79.2%)	146 (60.1%)	1,741 (77.2%)
**Atlantic Forest + Amazonia**	110 (5.5%)	42 (17.3%)	152 (6.7%)
**Atlantic Forest + Cerrado**	134 (6.7%)	20 (8.2%)	154 (6.8%)
**Atlantic Forest + Caatinga**	31 (1.5%)	0	31 (1.4%)
**Wide (3 or more biomes)**	143 (7.1%)	35 (14.4%)	178 (7.9%)
**Endangered species**	237 (11.8%)	19 (7.9%)	256 (11.3%)

## Discussion

### Diversity and distributions

Very high levels of vascular epiphytic species richness and endemism were found for the Atlantic Forest, with the vast majority of species being restricted to a small number of plant families. Taxonomic representation of vascular epiphytes for the Atlantic Forest is similar to that found for other tropical forests (Johansson 1989, Wallace 1989, Küper et al. 2004, Obermuller et al. 2014). Moreover, this concentration of epiphytic species in a small number of families in this biome is consistent with worldwide estimates. Orchidaceae, Bromeliaceae, Polypodiaceae and Araceae have had the most success in colonizing the canopy, as they account for 80% of global vascular epiphyte diversity (Kress 1986, Benzing 1990). In fact, the association of epiphytism with high rates of diversification was demonstrated taking into account phylogeny at various levels in both the Bromeliaceae and the Orchidaceae (Givnish et al. 2014, 2015). However, despite expressive species concentration into a few families, others also contribute to the diversity of epiphytic species, and there are a total of 33 families with at least one epiphytic species in the Atlantic Forest. Vascular epiphytes are distributed among 73–84 families worldwide (Kress 1986, Gentry and Dodson 1987, Zotz 2013), supporting the classic idea that epiphytism has evolved many times and in different plant groups (Lüttge 2008).

The high number of species and endemism rate of epiphytes found in the Brazilian Atlantic Forest follows the trend for this biome, one of the leading world hotspots for many groups of organisms including vascular plants (Myers et al. 2000). High diversity and endemism in the Atlantic Forest is frequently associated with high topographic complexity, abundant precipitation, and latitudinal range, favouring an increased variety of habitats (Gentry 1982, Werneck et al. 2011). Two divergent bioclimatic domains have been recognized within the Atlantic Forest, and endemism patterns of vertebrates have been associated with different climate features during the Pleistocene (Carnaval et al. 2014). The existence of areas with relative climatic stability (*refugia*) is at the center of the explanation for the phytogeographic endemism towards the north of the biome, while areas with climatic heterogeneity appear to be associated with the endemism in the south Atlantic Forest. In Myrtaceae, a representative group of trees in the Atlantic Forest, lower extinction rates were found to be associated with those refugium areas, while higher speciation rates were found in southern, unstable areas (Staggemeier et al. 2015). As epiphytes are intrinsically associated with forests, we can expect that similar patterns should be found in typical epiphytic lineages of the Atlantic Forest. In an attempt to answer this question, Fontoura et al. (2012) have found that species similarity among assemblages of epiphyte bromeliads was divided into one block of south–southeastern localities, and a second block of northeastern–southeastern localities, resembling the distribution of phorophyte species.

Nevertheless, a recurrent question regarding the value of focusing on biodiversity hotspots revolves around the conflicting results that often arise when comparing different groups (Prendergast et al. 1993, Kohlmann et al. 2010, Crain and Tremblay 2014). In the present case, 77% of the epiphytic vascular flora endemic to the Atlantic Forest (roughly 80% for angiosperms) contrasts with 57% of the overall vascular flora endemism and with around 60% of endemic tree species found for this biome (Forzza et al. 2010, List of Species of the Brazilian Flora 2014). Moreover, so far vascular epiphyte endemism is the highest registered for any organism group from the Atlantic Forest.This is more, for instance, than the ca. 60% found for amphibians and roughly 30% for mammals (Myers et al. 2000). Epiphytes are very peculiar organisms in terms of their environmental requirements, and three main factors (light, water, and mineral nutrients) limit epiphytic life (Lüttge 2008). Among them, water availability is considered to be the major constraint for epiphytes (Zotz and Hietz 2001), and this can be observed in the Atlantic Forest, where a drastic reduction in species richness and epiphyte abundance has occurred in the drier and more seasonal semi-deciduous forests that occur in the southwestern portion of the biome (Forzza et al. 2014). Accordingly, epiphytes are more abundant where precipitation and air moisture are high, such as in montane rainforests and upper cloud forests (Freiberg and Freiberg 2000).

This high rate of epiphytic endemism in the Atlantic Forest is reinforced by the contiguity of the xeric Caatinga biome that harbours few epiphytic species and may act as a barrier to biotic exchanges between the Atlantic Forest and the rainforest in the Amazonia (Prado and Gibbs 1993). Less than 7% of the epiphytic species (5.5% for angiosperms) are shared between these two biomes. Thus, even the gallery forest expansion that occurred during the Quaternary wet periods (Wang et al. 2004) does not seem to have been highly effective in enabling epiphyte flora exchange. This is the case for some Episcieae-Gesneriaceae clades (*Codonanthe* (Mart.) Hanst. and *Nematanthus* Schrad. lineages) that are absent in the Amazon, Cerrado and Caatinga, suggesting that the spread of a more open vegetation along a dry corridor separating the Atlantic Forest from the Amazon has been an efficient barrier to the dispersal of these groups from the early Miocene to the present day (Perrett et al. 2013). It also seems to have occurred among epiphytic cacti of the monophyletic tribe Rhipsalideae (genera *Lepismium* Pfeiff., *Rhipsalis* Gaertn., *Hatiora* Britton & Rose and *Schlumbergera* Lem.), mostly endemic to the Atlantic Forest (Calvente et al. 2011, Moreno et al. 2015). However some *Lepismium* species have disjunct distribution between the Atlantic Forest and the yungas forest of the eastern Andes, and *Rhipsalis baccifera* (J.M.Muell.) Stearn is the only cactus with an inter-continental distribution (Moreno et al. 2015). Nevertheless, the surrounding drier and/or more seasonal biomes of the Atlantic Forest may not represent such strong barriers for the dispersal of secondary hemi-epiphytes, which are relatively less dependent on air-humidity, at least in their initial seedling establishment stages. For instance, several Atlantic Forest aroid species of the Philodendron Schott subg. Philodendron lineage that probably diverged during the Latest Miocene/Pliocene are widely distributed, also occuring in the Amazon, Caatinga, and Cerrado (Oliveira 2014). In addition, the diversification of the Philodendron subg. Meconostigma Schott lineage into the Cerrado biome occurred during the Late Pliocene from Atlantic Forest ancestors (Oliveira 2014).

The historical discussion on geotemporal trajectories of plant diversification in Neotropical biomes has focused on two alternative models: the museum hypothesis, highlighting an ancient history of steady accumulation of diversity, and the cradle model, favouring more recent diversification and high speciation rates (reviewed in Hughes et al. 2013). More recent models and empirical evidence from dated phylogenetic trees combine episodes of rapid and slower diversification for Neotropical plants (see Linder 2008, Antonelli and Sanmartín 2011). There is very little fossil record of epiphytism and classic studies suggested that this habit might have developed fairly recently among vascular plants, with most diversification occurring during the Pliocene-Pleistocene (Benzing 1990, Lüttge 2006). Particularly, in the case of Orchidaceae, recent evidence based on broad-scale phylogeny indicates that epiphytism in the family appears to have evolved once at the base of the upper epidendroids (by far the most representative group of epiphyte orchids) no later than 35 Mya, with most diversification of subtribes from the Miocene on (Givnish et al. 2015). Among Bromeliaceae almost all cases of epiphytism can be traced to two origins: the first occurred at the base of Tillandsioideae, ca. 16.9–15.2 Mya, with dispersal from the Guayana Shield into the Andes, Central America, and/or the northern littoral of South America or Caribbean while the second one occurred more recently in the Late Miocene (ca. 5.9 Mya) in the Atlantic Forest, in the clade including tank bromelioids (Givnish et al. 2014).

The very high endemism rates found for Atlantic Forest epiphytes, in particular for predominantly holoepiphytic groups of angiosperms are in accordance with the idea of predominant recent diversification. This is the case of Bromeliaceae native to the Atlantic Forest, which rates of net diversification are especially high in the bromelioid tank epiphyte clade (1.11 and 1.05 Mya for stem and crown rates, respectively) and the core tillandsioids (i.e., Tillandsioideae minus *Catopsis* Griseb. and *Glomeropitcairnia* Mez; 0.47 and 0.67 Mya) (Givnish et al. 2014). Another example of this is a major clade of the Gesneriaceae tribe Episcieae mentioned above, composed of epiphytic genera *Codonanthe* and *Nematanthus* that are restricted to the Atlantic Forest. Most species in this clade have diverged from the Pliocene onwards (Perrett et al. 2013), coinciding with species of two subgenera of *Philodendron* Schott, Philodendron subg. Meconostigma and Philodendron subg. Philodendron (Oliveira 2014) and species of *Rhipsalis*, *Hatiora* and *Schlumbergera* (Cactaceae, tribe Rhipsalideae) (Arakaki et al. 2011, Moreno et al. 2015). Further, more refined studies which trace dated phylogenies at low taxonomic levels and relate determinants of net diversification, key innovations and invasion of specific ecological zones (e.g., Givnish et al. 2014) are necessary to test hypotheses of recent *in situ* diversification predominance of clades belonging to the major epiphytic angiosperm groups in the Atlantic Forest.

The Atlantic Forest holds the highest richness of fern and lycophyte species in Brazil (Prado and Sylvestre 2010), and the biome was defined by Tryon (1972) as one of the primary centres of Neotropical richness and endemism for non-flowering vascular plants. Specific endemism in ferns and lycophytes is known to be less expressive than endemism in flowering plants (Smith 1972). Propagules of these plants are light, wind-dispersed spores that can easily cross geographic barriers (Tryon 1972). Environmental conditions are, therefore, main features determining geographic ranges in fern and lycophyte species (Page 1979). This is evident in the distribution patterns found within the Atlantic Forest: ferns and lycophytes have more widespread species (species occurring in three or more biomes) than angiosperms. In contrast to the latter, fern and lycophyte species are more likely to occur in forest environments, such as the Atlantic Forest and the Amazon rather than in open, savannic habitats such as Cerrado and xeric Caatinga habitats.

### Conservation matters

Currently there are 78 fern and lycophyte species listed as threatened in Brazil (Martinelli and Moraes 2013), and, amongst these, 25% of them are Atlantic Forest epiphytes. Of these endangered species, eight (*Alansmia senilis* (Fée) Moguel & M.Kessler, *Ceradenia capillaris* (Desv.) L.E.Bishop, *Phlegmariurus aqualupianus* (Spring) B.Øllg., *Philodendron mollicomus* (Spring) B.Øllg., *Philodendron taxifolius* (Sw.) Á.Löve & D.Löve, *Stenogrammitis limula* (Christ) Labiak, *Terpsichore semihirsuta* (Klotzsch) A.R.Sm. and *Terpsichore taxifolia* (L.) A.R.Sm.) are not endemic to Brasil, presenting disjunct distribution between the Atlantic Forest and the Andes or with Mesoamerica. Considering the other 11 species, a single one, *Phlegmarius martii* (Wawra) B.Øllg., is not endemic to the Brazilian Atlantic Forest. Among epiphyte angiosperms, only 13 out of the 237 threatened species are not endemic to Brazil, as well as 20 species that are not endemic to the Atlantic Forest. All such endangered species have either a small area of occupancy or a small extent of occurrence, and/or are subject to threats due to habitat supression/ecosystem degradation (see Martinelli and Moraes 2013). Habitat supression resulting from human impact on vegetation plays an important role in epiphytic communities, as changes in moisture, irradiation and nutrient availability may limit survival and population dynamics of epiphytes (Hietz 1999, Werner et al. 2011). Less-structured secondary vegetation may not offer a similar range of microsites and the diversity of disturbances in space and time that are found in well established forests, and thus, they may be unsuitable for the establishment and survival of certain epiphytic species (Hietz 1999). For instance, mortality of vascular epiphytes was substantially increased on remnant trees in fragmented areas (72% over 3 years) relative to undisturbed forest (11%) in montane southern Ecuador (Werner 2011). Moreover, gradual changes in species richness and abundance of vascular epiphytes were detected from edge to inward at mixed ombrophilous (”Araucaria) Atlantic Forest, reflecting the gradients of light intensity, humidity and other environmental factors after habitat fragmentation (Bianchi and Kersten 2014).

Vascular epiphytes in the Atlantic Forest are possibly more prone to projected effects of global climate change in their area of occurrence, specifically with increased mean temperatures, and higher frequency of drastic events such as long dry periods (Marengo 2007). Such effects may affect air humidity levels required by vascular epiphytes to survive and the integrity of the vertical microclimatic gradient that is crucial for the maintenance of epiphyte diversity in forests, mainly in those under moderately seasonal climates (Werner et al. 2011), which predominate in the Atlantic Forest. In short, as pointed out by Prendergast et al. (1993), many hotspots are not effective conservation surrogates for rare or restricted species. Therefore, specific conservation actions are needed to ensure the welfare of this peculiar group of plants, in particular for obligate and preferential holoepiphytes.

## Conclusion

Vascular epiphytes are a characteristic feature of Neotropical forests, however, our understanding on biogeographical and floristic relationships of epiphytes as well as of the mechanisms that structure epiphyte communities are still rather poor (Kreft et al. 2004 Wagner et al. 2015). As in the majority of Neotropical forest sites (see Kreft et al. 2004), present knowledge on diversity, phytogeography and the community ecology of the Atlantic Forest is mainly based on studies of tree species (e.g., Oliveira-Filho and Fontes 2000, Santos et al. 2010, Eisenlohr et al. 2013). Trees represent about 22% of angiosperm species of the Atlantic Forest (List of Species of the Brazilian Flora 2014), which means that the vascular epiphytes have an equivalent specific diversity (roughly 15%) of the trees found in this biome. Moreover, the high occurrence of endemisms, roughly 77%, is an outstanding feature among vascular epiphytes of the Atlantic Forest, which harbours roughly 7.5 to 10% of the total known vascular epiphytic flora worldwide. These general patterns should be viewed as starting points for further studies focusing on processes and mechanisms to better understand the role of the epiphytes in the dynamics of communities of the Atlantic Forest, as well as the evolution of the most representative groups of Neotropical epiphytes. For instance, studies contrasting the geographic distribution and diversity patterns of epiphytes and trees within the biome, dated phylogenies of those epiphytic clades highly distributed in the biome, and studies on diversification rates, key innovations and functional traits of epiphytes in the assembly of communities are fundamental for answering these questions.

## Supplementary materials

Supplementary material 1 Species list of vascular (hemi-)epiphytes of Brazilian Atlantic Forest Data type: species list. This dataset is made available under the Open Database License (http://opendatacommons.org/licenses/odbl/1.0/). The Open Database License (ODbL) is a license agreement intended to allow users to freely share, modify, and use this Dataset while maintaining this same freedom for others, provided that the original source and author(s) are credited.Leandro Freitas, Alexandre Salino, Luiz Menini Neto, Thaís Elias Almeida, Sara Ribeiro Mortara, João Renato Stehmann, André Marcio Amorim, Elsie Franklin Guimarães, Marcus Nadruz Coelho, Ana Zanin, Rafaela Campostrini Forzza

## References

[B1] Antonelli A Sanmartín I (2011) Why are there so many plant species in the Neotropics? Taxon 60: 403–414.

[B2] Arakaki M Christin P-A Nyffeler R Lendel A Eggli U Ogburn RM Spriggs E Moore MJ Edwards EJ (2011) Contemporaneous and recent radiations of the world’s major succulent plant lineages. Proceedings of the National Academy of Sciences of the United States of America 108: 8379–8384. doi: 10.1073/pnas.1100628108 21536881 10.1073/pnas.1100628108 PMC3100969

[B3] Barker MG Pinard MA (2001) Forest canopy research: sampling problems, and some solutions. Plant Ecology 152: 23–38. doi: 10.1023/A:1017584130692

[B4] Bartels SF Chen HYH (2012) Mechanisms regulating epiphytic plant diversity. Critical Reviews in Plant Sciences 31: 391–400. doi: 10.1080/07352689.2012.680349

[B5] Benzing DH (1990) Vascular epiphytes. Cambridge University Press, New York, 376 pp. doi: 10.1017/cbo9780511525438

[B6] Benzing DH (2000) Bromeliaceae: profile of an adaptive radiation. Cambridge University Press, New York, 690 pp. doi: 10.1017/cbo9780511565175

[B7] Bianchi JS Kersten RA (2014) Edge effect on vascular epiphytes in a subtropical Atlantic Forest. Acta Botanica Brasilica 28: 120–126. doi: 10.1590/S0102-33062014000100012

[B8] Burns KC Zotz G (2010) A hierarchical framework for investigating epiphyte assemblages: networks, meta-communities, and scale. Ecology 91: 377–385. doi: 10.1890/08-2004.1 20392003 10.1890/08-2004.1

[B9] Calvente A Zappi DC Forest F Lohmann LG (2011) Molecular phylogeny of tribe Rhipsalideae (Cactaceae) and taxonomic implications for *Schlumbergera* and *Hatiora*. Molecular Phylogenetics and Evolution 58: 456–68. doi: 10.1016/j.ympev.2011.01.001 21236350 10.1016/j.ympev.2011.01.001

[B10] Câmara IG (2003) Brief history of conservation in the Atlantic Forest. In: Galindo-Leal C Câmara IG (Eds) The Atlantic Forest of South America: Biodiversity Status, Trends, and Outlook. Center for Applied Biodiversity Science and Island Press, Washington, DC, 31–42.

[B11] Carnaval AC Waltari E Rodrigues MT Rosauer D VanDerWal J Damasceno R Prates I Strangas M Spanos Z Rivera D Pie MR Firkowski CR Bornschein MR Ribeiro LF Moritz C (2014) Prediction of phylogeographic endemism in an environmentally complex biome. Proceedings of the Royal Society B: Biological sciences 281: 1471–2954. doi: 10.1098/rspb.2014.1461 10.1098/rspb.2014.1461 PMC4150330 25122231

[B12] Crain BJ Tremblay RL (2014) Do richness and rarity hotspots really matter for orchid conservation in light of anticipated habitat loss? Diversity and Distributions 20: 652–662. doi: 10.1111/ddi.12179

[B13] Currie DJ Mittelbach GG Cornell HV Field R Guégan JF Hawkins BA Kaufman DM Kerr JT Oberdorff T O’Brien E Turner JRG (2004) Predictions and tests of climate-based hypotheses of broad-scale variation in taxonomic richness. Ecology Letters 7: 1121–1134. doi: 10.1111/j.1461-0248.2004.00671.x

[B14] Eisenlohr PV Alves LF Bernacci LC Padgurschi MCG Torres RB Prata EMB Santos FAM Assis MA Ramos E Rochelle ALC Martins FR Campos MCR Pedroni F Sanchez M Pereira LS Vieira SA Gomes JAMA Tamashiro JY Scaranello MAS Caron CJ Joly CA (2013) Disturbances, elevation, topography and spatial proximity drivevegetation patterns along an altitudinal gradient of a top biodiversity hotspot. Biodiversity and Conservation 22: 2767–2783. doi: 10.1007/s10531-013-0553-x

[B15] Fontoura T Scudeller VV Costa AF (2012) Floristics and environmental factors determining the geographic distribution of epiphytic bromeliads in the Brazilian Atlantic Rain Forest. Flora 207: 662–672. doi: 10.1016/j.flora.2012.05.003

[B16] Forzza RC Baumgratz JFA Bicudo CEM Canhos DAL Carvalho AA Coelho MAN Costa AF Costa DP Hopkins MG Leitman PM Lohmann LG Lughadha EN Maia LC Martinelli G Menezes M Morim MP Peixoto AL Pirani JR Prado J Queiroz LP Souza S Souza VC Stehmann JR Sylvestre LS Walter BMT Zappi DC (2012) New Brazilian floristic list highlights conservation challenges. BioScience 62: 39–45. doi: 10.1525/bio.2012.62.1.8

[B17] Forzza RC Leitman PM Costa AF Carvalho Jr AA Peixoto AL Walter BMT Bicudo C Zappi D Costa DP Lleras E Martinelli G Lima HC Prado J Stehmann JR Baumgratz JFA Pirani JR Sylvestre LS Maia LC Lohmann LG Paganucci L Silveira M Nadruz M Mamede MCH Bastos MNC Morim MP Barbosa MR Menezes M Hopkins M Secco R Cavalcanti T Souza VC (2010) The Brazilian catalogue of plants and funghi. Andrea Jakobsson: Jardim Botânico do Rio de Janeiro, Rio de Janeiro, 870 pp.

[B18] Forzza RC Pifano DS Oliveira-Filho A Meireles L Faria PCL Salimena FRG Mynssen CM Prado J (2014) Flora vascular da Reserva Biológica da Represa do Grama, Descoberto, Minas Gerais e suas relações florísticas com Florestas Ombrófilas e Semidecíduas do sudeste brasileiro. Rodriguésia 65: 275–292. doi: 10.1590/S2175-78602014000200001

[B19] Freiberg M Freiberg E (2000) Epiphyte diversity and biomass in the canopy of lowland and montane forests in Ecuador. Journal of Tropical Ecology 16: 673–688. doi: 10.1111/j.1744-7429.2010.00745.x

[B20] Gentry AH (1982) Neotropical floristic diversity: phytogeographical connections between central and South America, Pleistocene climatic fluctuations, or an accident of the Andean orogeny? Annals of Missouri Botanical Garden 69: 557–593. doi: 10.2307/2399084

[B21] Gentry AH Dodson CH (1987) Diversity and biogeography of Neotropical vascular epiphytes. Annals of the Missouri Garden 74: 205–233. doi: 10.1111/j.1365-2699.2004.01083.x

[B22] Givnish TJ Barfuss MHJ Van Ee B Riina R Schulte K Horres R Gonsiska PA Jabaily RS Crayn DM Smith JAC Winter K Brown GK Evans TM Holst BK Luther H Till W Zizka G Berry PE Sytsma KJ (2014) Adaptive radiation, correlated and contingent evolution, and net species diversification in Bromeliaceae. Molecular Phylogenetics and Evolution 71: 55–78. doi: 10.1016/j.ympev.2013.10.010 24513576 10.1016/j.ympev.2013.10.010

[B23] Givnish TJ Spalink D Ames M Lyon SP Hunter SJ Zuluaga A Iles WJD Clements MA Arroyo MTK Leebens-Mack J Endara L Kriebel R Neubig KM Whitten WM Williams NH Cameron KM (2015) Orchid phylogenomics and multiple drivers of their extraordinary diversification. Proceedings of the Royal Society B282: . doi: 10.1098/rspb.2015.1553 10.1098/rspb.2015.1553 PMC4571710 26311671

[B24] Hietz P (1999) Diversity and conservation of epiphytes in a changing environment. In: Proceedings International Conference on Biodiversity and Bioresources: Conservation and Utilization. Phuket, Thailand, 1–11.

[B25] Hughes CE Pennington RT Antonelli A (2013) Neotropical plant evolution: assembling the big picture. Botanical Journal of the Linnean Society 171: 1–18. doi: 10.1111/boj.12006

[B26] Johansson D (1989) Vascular epiphytes in Africa. In: Lieth H Werger MJA (Eds) Ecosystems of the world (v. 14b): Tropical Rain Forest ecosystems. Elsevier, Amsterdam, 183–194. doi: 10.1016/B978-0-444-42755-7.50015-8

[B27] Kamino LHY Stehmann JR Amaral S De Marco P Rangel TF Siqueira MF De Giovanni R Hortal J (2012) Challenges and perspectives for species distribution modelling in the Neotropics. Biology letters 8: 324–6. doi: 10.1098/rsbl.2011.0942 22031720 10.1098/rsbl.2011.0942 PMC3367727

[B28] Kelly DL O’Donovan G Feehan J Murphy S Drangeid SO Marcano-Berti L Marcano (2004) The epiphyte communities of a montane rain forest in the Andes of Venezuela: patterns in the distribution of the flora. Journal of Tropical Ecology 20: 643–666. http://www.jstor.org/stable/4092110

[B29] Kohlmann B Roderus D Elle O Solís Á Soto X Russo R (2010) Biodiversity conservation in Costa Rica: A correspondence analysis between identified biodiversity hotspots (Araceae, Arecaceae, Bromeliaceae, and Scarabaeinae) and conservation priority life zones. Revista Mexicana de Biodiversidad 81: 511–559.

[B30] Kreft H Jetz W (2007) Global patterns and determinants of vascular plant diversity. Proceedings of the National Academy of Sciences of the United States of America 104: 5925–5930. doi: 10.1073/pnas.0608361104 17379667 10.1073/pnas.0608361104 PMC1851593

[B31] Kreft H Köster N Küper W Nieder J Barthlott W (2004) Diversity and biogeography of vascular epiphytes in Western Amazonia, Yasuní, Ecuador. Journal of Biogeography 31: 1463–1476. doi: 10.1111/j.1365-2699.2004.01083.x

[B32] Kress WJ (1986) The systematic distribution of the vascular epiphytes: an update. Selbyana 9: 2–22. doi: 10.1111/boj.12010

[B33] Krömer T Kessler M Gradstein RS Acebey A (2005) Diversity patterns of vascular epiphytes along an elevational gradient in the Andes. Journal of Biogeography 32: 1799–1809. doi: 10.1111/j.1365-2699.2005.01318.x

[B34] Küper W Kreft H Nieder J Körter N Bartholott W (2004) Large-scale diversity patterns of vascular epiphytes in Neotropical montane rain forests. Journal of Biogeography 31: 1477–1487. doi: 10.1111/j.1365-2699.2004.01093.x

[B35] Linder HP (2008) Plant species radiations: where, when, why? Proceedings of the Royal Society B: Biological Sciences 363: 3097–3105. doi: 10.1098/rstb.2008.0075 10.1098/rstb.2008.0075 PMC2607312 18579472

[B36] List of Species of the Brazilian Flora (2014) Rio de Janeiro Botanical Garden. http://floradobrasil.jbrj.gov.br/ [accessed 31.09.2014]

[B37] Lüttge U (2008) Physiological ecology of tropical plants. Springer, Berlin, Heidelberg, 458 pp.

[B38] Marengo JA (2007) Caracterização do clima no Século XX e Cenários Climáticos no Brasil e na América do Sul para o Século XXI derivados dos Modelos Globais de Clima do IPCC. Relatório, Ministério do Meio Ambiente (SBF-MMA) https://www.scribd.com/fullscreen/7511437?access_key=key-2dwfvw0u4y9to6ttj9y [accessed 31.03.2014]

[B39] Martinelli G Moraes MA (2013) Livro vermelho da flora do Brasil. Andrea Jakobsson: Jardim Botânico do Rio de Janeiro, Rio de Janeiro, 1100 pp.

[B40] Moreno NC Amarilla LD Las Peñas ML Bernardello G (2015) Molecular cytogenetic insights into the evolution of the epiphytic genus *Lepismium* (Cactaceae) and related genera. Botanical Journal of the Linnean Society 177: 263–277. doi: 10.1111/boj.12242

[B41] Myers N Mittermeier RA Mittermeier CG Fonseca GAB Kent J (2000) Biodiversity hotspots for conservation priorities. Nature 403: 853–858. doi: 10.1038/35002501 10706275 10.1038/35002501

[B42] Obermuller FA Freitas L Daly DC Silveira M (2014) Patterns of diversity and gaps in vascular (hemi-)epiphyte flora of Southwestern Amazonia. Phytotaxa 166: 259–272. doi: 10.11646/phytotaxa.166.4.2

[B43] Oliveira LL (2014) Filogenia molecular, evolução do gineceu e biogeografia histórica do gênero *Philodendron* (Araceae). PhD Thesis, Universidade Federal do Rio de Janeiro, Brazil.

[B44] Oliveira-Filho AT Fontes MAL (2000) Patterns of floristic differentiation among Atlantic forests in south-eastern Brazil, and the influence of climate. Biotropica 32: 793–810. doi: 10.1646/0006-3606(2000)032[0793:POFDAA]2.0.CO;2

[B45] Page CN (1979) Experimental aspects of fern ecology. In: Dyer AF (Ed.) The experimental biology of ferns. Academic Press, London, 551–589.

[B46] Perret M Chautems A Araujo AO Salamin N (2013) Temporal and spatial origin of Gesneriaceae in the New World inferred from plastid DNA sequences. Botanical Journal of the Linnean Society 171: 61–79. doi: 10.1111/j.1095-8339.2012.01303.x

[B47] Prado DE Gibbs PE (1993) Patterns of species distributions in the dry seasonal forest South America. Annals of the Missouri Botanical Garden 80: 902–927. doi: 10.2307/2399937

[B48] Prado J Sylvestre LS (2010) Pteridófitas. In: Forzza RC Leitman PM Costa AF Carvalho Jr AA Peixoto AL Walter BMT Bicudo C Zappi D Costa DP Lleras E Martinelli G Lima HC Prado J Stehmann JR Baumgratz JFA Pirani JR Sylvestre LS Maia LC Lohmann LG Paganucci L Silveira M Nadruz M Mamede MCH Bastos MNC Morim MP Barbosa MR Menezes M Hopkins M Secco R Cavalcanti T Souza VC (Eds) Catálogo de Plantas e Fungos do Brasil. Jardim Botânico do Rio de Janeiro, Rio de Janeiro, 522–526.

[B49] Prendergast JR Quinn RM Lawton JH Eversham BC Gibbons DW (1993) Rare species, the coincidence of diversity hotspots and conservation strategies. Nature 365: 335–337. doi: 10.1038/365335a0

[B50] Ribeiro MC Metzger JP Martensen AC Ponzoni FJ Hirota MM (2009) The Brazilian Atlantic Forest: how much is left, and how is the remaining forest distributed? Implications for conservation. Biological Conservation 142: 1141–1153. doi: 10.1016/j.biocon.2009.02.021

[B51] Richardson BA (1999) The bromeliad microcosm and the assessment of faunal diversity in a Neotropical forest. Biotropica 31: 321–336. doi: 10.1111/j.1744-7429.1999.tb00144.x

[B52] Ricklefs RE (2004) A comprehensive framework for global patterns in biodiversity. Ecology Letters 7: 1–15. doi: 10.1046/j.1461-0248.2003.00554.x

[B53] Santos BA Arroyo-Rodríguez V Moreno CE Tabarelli M (2010) Edge-related loss of tree phylogenetic diversity in the severely fragmented Brazilian Atlantic Forest. PLoS ONE 5: . doi: 10.1371/journal.pone.0012625 10.1371/journal.pone.0012625 PMC2935881 20838613

[B54] Schuettpelz E Trapnell DW (2006) Exceptional epiphyte diversity on a single tree in Costa Rica. Selbyana 27: 65–71. doi: 10.2307/41760262

[B55] Smith AR (1972) Comparison of fern and flowering plant distributions with some evolutionary interpretations for ferns. Biotropica 4: 4–9. doi: 10.2307/2989639

[B56] Staggemeier VG Diniz-Filho JAF Forest F Lucas E (2015) Phylogenetic analysis in Myrcia section Aulomyrcia and inferences on plant diversity in the Atlantic rainforest. Annals of Botany 115: 747–61. doi: 10.1093/aob/mcv005 25757471 10.1093/aob/mcv005 PMC4373287

[B57] Stehman JR Forzza RC Salino A Sobral M Costa DP Kamino LHY (2009) Plantas da Floresta Atlântica. Jardim Botânico do Rio de Janeiro, Rio de Janeiro, 505 pp.

[B58] Tryon R (1972) Endemic areas and geographic speciation in tropical American ferns. Biotropica 4: 121–131. doi: 10.2307/2989774

[B59] Wagner K Mendieta-Leiva G Zotz G (2015) Host specificity in vascular epiphytes: a review of methodology, empirical evidence and potential mechanisms. AoB PLANTS 7: . doi: 10.1093/aobpla/plu092 10.1093/aobpla/plu092 PMC4306756 25564514

[B60] Wallace BJ (1989) Vascular epiphytism in Australo-Asia. In: Lieth H Werger MJA (Eds) Ecosystems of the world, v. 14b: Tropical Rain Forest ecosystems. Elsevier, Amsterdam, 261–282.

[B61] Wang X Auler AS Edwards RL Cheng H Cristalli PS Smart PL Richards DA Shen CC (2004) Wet periods in northeastern Brazil over the past 210 kyr linked to distant climate anomalies. Nature 432: 740–743. doi: 10.1038/nature03067 15592409 10.1038/nature03067

[B62] Werneck MDS Sobral MEG Rocha CTV Landau EC Stehmann JR (2011) Distribution and endemism of angiosperms in the Atlantic Forest. Natureza & Conservação 9: 188–193. doi: 10.4322/natcon.2011.024

[B63] Werner FA (2011) Reduced growth and survival of vascular epiphytes on isolated remnant trees in a recent tropical montane forest clear-cut. Basic and Applied Ecology 12: 172–181. doi: 10.1016/j.baae.2010.11.002

[B64] Werner FA Kòster N Kessler M Gradstein SR (2011) Is the resilience of epiphyte assemblages to human disturbance a function of local climate? Ecotropica 17: 15–20. doi: 10.5167/uzh-76892

[B65] Zotz G (2013) The systematic distribution of vascular epiphytes - a critical update. Botanical Journal of the Linnean Society 171: 453–481. doi: 10.1111/boj.12010

[B66] Zotz G Hietz P (2001) The physiological ecology of vascular epiphytes: current knowledge, open questions. Journal of Experimental Botany 52: 2067–2078. doi: 10.1093/jexbot/52.364.2067 11604445 10.1093/jexbot/52.364.2067

