# Supplementary material for: A comprehensive checklist of vascular epiphytes of the Atlantic Forest reveals outstanding endemic rates
Source: PhytoKeys. 2016 Jan 12;(58):65–79. doi: 10.3897/phytokeys.58.5643 (PMC4743015; doi:10.3897/phytokeys.58.5643)
Supplement: Supplementary material 1 — Species list of vascular (hemi-)epiphytes of Brazilian Atlantic Forest [file phytokeys-058-065-s001.doc]

**Supplemental online material File 1**.

Table. Species list of vascular (hemi-)epiphytes of Brazilian Atlantic Forest. Abbreviations for Habit: Ep – epiphyte; Hep – hemi-epiphyte; Threatened category (Thr-Cat) after Martinelli and Moraes (2013): CR – critical endangered; EN – endangered; VU – vulnerable; and Occurrence: BR – endemic to Brazil; AF – Atlantic Forest; Am – Amazonia; Ce – Cerrado; Ca – Caatinga.

|  |  |  |  | **Occurrence** | | | | |
| --- | --- | --- | --- | --- | --- | --- | --- | --- |
| **Family** | **Species** | **Habit** | **Thr-Cat** | **BR** | **AF** | **Am** | **Ce** | **Ca** |
| **Acanthaceae** | *Clistax bahiensis* Profice & Leitmam | Ep |  | X | X |  |  |  |
| **Amaryllidaceae** | *Hippeastrum aulicum* Herb. | Ep |  | X | X |  |  |  |
|  | *Hippeastrum calyptratum* Herb. | Ep |  | X | X |  |  |  |
| **Araceae** | *Anthurium bocainense* Catharino & Nadruz | Ep |  | X | X |  |  |  |
|  | *Anthurium boudetii* Nadruz | Ep |  | X | X |  |  |  |
|  | *Anthurium brachypodum* G.Barroso | Ep |  | X | X | X |  |  |
|  | *Anthurium bromelicola* Mayo & l.P.Felix | Ep |  | X | X |  |  | X |
|  | *Anthurium comtum* Schott | Ep |  | X | X |  |  |  |
|  | *Anthurium gaudichaudianum* Kunth | Ep |  | X | X |  |  |  |
|  | *Anthurium gladiifolium* Schott | Ep |  | X | X |  |  |  |
|  | *Anthurium gomesianum* Nadruz | Ep |  | X | X |  |  |  |
|  | *Anthurium harrisii* (Graham) Endl. | Ep |  | X | X |  |  |  |
|  | *Anthurium ianthinopodium* (Schott ex Engl.) Nadruz & Mayo | Ep |  | X | X |  |  |  |
|  | *Anthurium intermedium* Kunth | Ep |  | X | X |  |  |  |
|  | *Anthurium itanhaense* Engl. | Ep |  | X | X |  |  |  |
|  | *Anthurium jilekii* Schott | Ep |  | X | X |  |  | X |
|  | *Anthurium langsdorffii* Schott | Ep | EN | X | X |  |  |  |
|  | *Anthurium leonii* E.G.Gonç. | Ep |  | X | X |  |  |  |
|  | *Anthurium lhotzkyanum* Schott | Ep |  | X | X |  |  |  |
|  | *Anthurium longifolium* (Hoffmanns.) G.Don | Ep |  | X | X |  |  |  |
|  | *Anthurium lucidum* Kunth | Ep | EN | X | X |  |  |  |
|  | *Anthurium minarum* Sakur. & Mayo | Ep |  | X | X |  | X |  |
|  | *Anthurium miquelianum* C.Koch & Augustin | Ep |  | X | X |  |  |  |
|  | *Anthurium organense* Engl. | Ep |  | X | X |  |  |  |
|  | *Anthurium parvum* N.E.Br. | Ep |  | X | X |  |  |  |
|  | *Anthurium pentaphyllum* (Aubl.) G.Don | Ep |  |  | X | X |  |  |
|  | *Anthurium santaritensis* Nadruz & Croat | Ep |  | X | X |  |  |  |
|  | *Anthurium scandens* (Aubl.) Engl. | Ep |  |  | X | X | X | X |
|  | *Anthurium sellowianum* Kunth | Ep |  | X | X |  |  |  |
|  | *Anthurium sinuatum* Benth. ex Schott | Ep |  |  | X | X | X | X |
|  | *Anthurium solitarium* Schott | Ep |  |  | X | X | X |  |
|  | *Anthurium undatum* Schott | Ep |  | X | X |  |  |  |
|  | *Anthurium urvilleanum* Schott | Ep |  | X | X |  |  |  |
|  | *Heteropsis flexuosa* (Kunth) G.S.Bunting | Hep | VU |  | X | X |  |  |
|  | *Heteropsis oblongifolia* Kunth | Hep |  |  | X | X | X |  |
|  | *Heteropsis rigidifolia* Engl. | Hep |  | X | X |  |  |  |
|  | *Heteropsis salicifolia* Kunth | Hep |  | X | X |  |  |  |
|  | *Monstera adansonii* Schott | Hep |  |  | X | X | X | X |
|  | *Monstera obliqua* Miq. | Hep |  |  | X | X |  |  |
|  | *Monstera praetermissa* E.G.Gonç. & Temponi | Hep |  | X | X | X |  |  |
|  | *Philodendron acutatum* Schott | Ep |  |  | X | X | X | X |
|  | *Philodendron aemulum* Schott | Hep |  | X | X |  |  |  |
|  | *Philodendron alternans* (Vell.) Schott | Hep |  | X | X |  |  |  |
|  | *Philodendron altomacaense* Nadruz & Mayo | Hep |  | X | X |  |  |  |
|  | *Philodendron appendiculatum* Nadruz & Mayo | Hep |  | X | X |  |  |  |
|  | *Philodendron bernardopazii* E.G.Gonç. | Hep |  | X | X |  |  |  |
|  | *Philodendron bipennifolium* Schott | Hep |  |  | X | X |  |  |
|  | *Philodendron bipinnatifidum* Schott | Hep |  |  | X |  | X | X |
|  | *Philodendron blanchetianum* Schott | Hep |  | X | X |  |  |  |
|  | *Philodendron corcovadense* Kunth | Hep |  | X | X |  |  |  |
|  | *Philodendron cordatum* Schott | Hep |  | X | X |  |  |  |
|  | *Philodendron crassinervium* Lindl. | Hep |  | X | X |  |  |  |
|  | *Philodendron curvilobum* Schott | Hep |  | X | X |  |  |  |
|  | *Philodendron edmundoi* G.Barroso | Hep |  | X | X |  |  |  |
|  | *Philodendron eximium* Schott | Hep |  | X | X |  |  |  |
|  | *Philodendro follii* Nadruz | Hep |  | X | X |  |  |  |
|  | *Philodendron fragile* Nadruz & Mayo | Hep | EN | X | X |  |  |  |
|  | *Philodendron fragrantíssimo* (Hook.) G.Don | Hep |  |  | X | X |  |  |
|  | *Philodendron glaziovii* Hook.f. | Hep |  | X | X |  |  |  |
|  | *Philodendron hastatum* K.Koch & Sello | Hep |  | X | X |  |  |  |
|  | *Philodendron hatschbachii* Nadruz & Mayo | Hep |  | X | X |  |  |  |
|  | *Philodendron hederaceum* (Jacq.) Schott | Hep |  |  | X | X |  |  |
|  | *Philodendron inops* Schott | Hep |  | X | X |  |  |  |
|  | *Philodendron insigne* Schott | Ep |  |  | X | X |  |  |
|  | *Philodendron kautskyi* G.S.Bunting | Hep |  | X | X |  |  |  |
|  | *Philodendron leal-costae* Mayo & G.Barroso | Hep |  | X | X |  |  | X |
|  | *Philodendron loefgrenii* Engl. | Hep |  | X | X |  |  |  |
|  | *Philodendron longilaminatum* Schott | Hep |  | X | X |  |  |  |
|  | *Philodendron longilobatum* Sakur. | Hep |  | X | X |  |  |  |
|  | *Philodendron martianum* Engl. | Hep |  | X | X |  |  |  |
|  | *Philodendron meridionale* Buturi & Sakur. | Hep |  | X | X |  |  |  |
|  | *Philodendron millerianum* Nadruz & Sakur. | Hep |  | X | X |  |  |  |
|  | *Philodendron minarum* Engl. | Hep |  | X | X |  | X |  |
|  | *Philodendron missionum* (Hauman) Hauman | Hep |  | X | X |  |  |  |
|  | *Philodendron nadruzianum* Sakur. | Hep |  | X | X |  |  |  |
|  | *Philodendron oblongum* (Vell.) Kunth | Hep |  | X | X | X | X |  |
|  | *Philodendron ochrostemon* Schott | Hep |  | X | X |  |  |  |
|  | *Philodendron ornatum* Schott | Hep |  |  | X | X |  | X |
|  | *Philodendron pedatum* (Hook.) Kunth | Hep |  |  | X | X | X | X |
|  | *Philodendron propinquum* Schott | Hep |  | X | X |  |  |  |
|  | *Philodendron recurvifolium* Schott | Hep |  | X | X |  |  |  |
|  | *Philodendron renauxii* Reitz | Hep |  | X | X |  |  |  |
|  | *Philodendron rhodospermum* Calazans & Sakur. | Hep |  | X | X |  |  |  |
|  | *Philodendron roseopetiolatum* Nadruz & Mayo | Hep |  | X | X |  |  |  |
|  | *Philodendron rudgeanum* Schott | Hep |  |  | X | X |  |  |
|  | *Philodendron ruthianum* Nadruz | Hep |  | X | X |  |  |  |
|  | *Philodendron simonianum* Sakur. | Hep |  | X | X |  |  |  |
|  | *Philodendron speciosum* Schott ex Endl. | Hep |  | X | X |  |  |  |
|  | *Philodendron spirictus-sancti* G.S.Bunting | Hep | EN | X | X |  |  |  |
|  | *Philodendron stenolobum* E.G.Gonç. | Hep |  | X | X |  |  |  |
|  | *Philodendron surinamense* (Miq.) Engl. | Hep |  |  | X | X |  |  |
|  | *Philodendron tenuispadix* E.G.Gonç. | Hep |  | X | X |  |  |  |
|  | *Philodendron williamsii* Hook.f. | Hep |  | X | X |  |  |  |
|  | *Rhodospatha latifolia* Poepp. | Hep |  | X | X | X | X | X |
|  | *Rhodospatha oblongata* Poepp. | Hep |  | X | X | X |  | X |
|  | *Stenospermation spruceanum* Schott | Ep |  |  | X | X | X |  |
|  | *Syngonium podophyllum* Schott | Hep |  |  | X | X |  |  |
|  | *Syngonium vellozianum* Schott | Hep |  | X | X |  |  |  |
| **Araliaceae** | *Dendropanax cuneatus* (DC.) Decne. & Planch | Hep |  |  | X | X | X | X |
|  | *Dendropanax geniculatus* Fiaschi | Hep |  | X | X |  |  |  |
|  | *Oreopanax capitatus* (Jacq.) Decne. & Planch. | Hep |  |  | X | X |  |  |
| **Aspleniaceae** | *Asplenium abscissum* Willd. | Ep |  |  | X |  | X |  |
|  | *Asplenium auriculatum* Sw. | Ep |  |  | X | X | X |  |
|  | *Asplenium auritum* Sw. | Ep |  |  | X | X | X |  |
|  | *Asplenium austrobrasiliense* (Christ) Maxon | Ep |  | X | X |  |  |  |
|  | *Asplenium badinii* L.Sylvestre & P.G.Windisch | Ep |  | X | X |  |  |  |
|  | *Asplenium bradeanum* Handro | Ep | EN | X | X |  |  |  |
|  | *Asplenium cariocanum* Brade | Ep |  | X | X |  |  |  |
|  | *Asplenium cirrhatum* Rich. ex Willd. | Ep |  |  | X | X |  |  |
|  | *Asplenium feei* Kunze ex Fée | Ep |  |  | X | X |  |  |
|  | *Asplenium formosum* Willd. | Ep |  |  | X | X | X |  |
|  | *Asplenium gastonis* Fée | Ep |  |  | X |  |  |  |
|  | *Asplenium harpeodes* Kunze | Ep |  |  | X |  |  |  |
|  | *Asplenium incurvatum* Fée | Ep |  | X | X |  |  |  |
|  | *Asplenium jucundum* Fée | Ep |  | X | X |  |  |  |
|  | *Asplenium juglandifolium* Lam. | EP |  |  | X | X |  |  |
|  | *Asplenium kunzeanun* Klotzsch ex Rosenst. | Ep |  | X | X |  |  |  |
|  | *Asplenium mucronatum* C.Presl | Ep |  |  | X |  |  |  |
|  | *Asplenium muellerianum* Rosenst. | Ep |  | X | X |  |  |  |
|  | *Asplenium oligophyllum* Kaulf. | Ep |  |  | X |  |  |  |
|  | *Asplenium praemorsum* Sw. | Ep |  |  | X |  | X |  |
|  | *Asplenium pteropus* Kaulf. | Ep |  |  | X |  |  |  |
|  | *Asplenium raddianum* Gaudich. | Ep |  |  | X |  |  |  |
|  | *Asplenium salicifolium* L. | Ep |  |  | X | X | X |  |
|  | *Asplenium scandicinum* Kaulf. | Ep |  |  | X |  |  |  |
|  | *Asplenium serra* Langsd. & Fisch. | Ep |  |  | X | X |  |  |
|  | *Asplenium serratum* L. | Ep |  |  | X | X | X |  |
|  | *Asplenium stuebelianum* Hieron. | Ep |  |  | X | X | X |  |
|  | *Asplenium theciferum* (Kunth) Mett. | Ep |  |  | X |  |  |  |
|  | *Asplenium truncorum* F.B.Matos, Labiak & Sylvestre | Ep |  | X | X |  |  |  |
| **Begoniaceae** | *Begonia coccinea* Hook. | Ep | EN | X | X |  |  |  |
|  | *Begonia convolvulacea* (Klotzsch) A.DC. | Ep |  | X | X |  |  |  |
|  | *Begonia dentatiloba* A.DC. | Ep | EN | X | X |  |  |  |
|  | *Begonia depauperata* Schott | Ep |  | X | X |  |  |  |
|  | *Begonia fruticosa* (Klotzsch) A.DC. | Ep |  | X | X |  |  |  |
|  | *Begonia fulvosetulosa* Brade | Ep |  | X | X |  |  |  |
|  | *Begonia herbacea* Vell. | Ep |  | X | X |  |  |  |
|  | *Begonia hirtella* Link | Ep |  |  | X | X |  |  |
|  | *Begonia integerrima* Spreng. | Ep |  | X | X |  |  |  |
|  | *Begonia itaguassuencis* Brade | Hep |  | X | X |  |  |  |
|  | *Begonia lanceolata* Vell. | Ep |  | X | X |  |  |  |
|  | *Begonia obdeltata* Gregório & E.L.Jacques | Ep |  | X | X |  |  |  |
|  | *Begonia radicans* Vell. | Ep |  | X | X |  |  |  |
|  | *Begonia smilacina* A.DC. | Ep | EN | X | X |  |  |  |
|  | *Begonia solananthera* A.DC. | Ep |  | X | X |  |  |  |
| **Blechnaceae** | *Blechnum acutum* (Desv.) Mett. | Hep |  |  | X |  |  |  |
| **Bromeliaceae** | *Acanthostachys pitcairnioides* (Mez) Rauh & Barthlott | Ep |  | X | X |  |  |  |
|  | *Acanthostachys strobilacea* (Schultes & Schultes f.) Klotzsch | Ep |  |  | X |  | X |  |
|  | *Aechmea aiuruocensis* Leme | Ep |  | X | X |  |  |  |
|  | *Aechmea alba* Mez | Ep |  | X | X |  |  |  |
|  | *Aechmea alopecurus* Mez | Ep | EN | X | X |  |  |  |
|  | *Aechmea altocaririensis* Leme & L.Kollmann | Ep |  | X | X |  |  |  |
|  | *Aechmea amorimii* Leme | Ep |  | X | X |  |  |  |
|  | *Aechmea ampla* L.B.Sm. | Ep |  | X | X |  |  |  |
|  | *Aechmea andersoniana* Leme & H.Luther | Ep |  | X | X |  |  |  |
|  | *Aechmea andersonii* H.Luther & Leme | Ep |  | X | X |  |  |  |
|  | *Aechmea apocalyptica* Reitz | Ep | VU | X | X |  |  |  |
|  | *Aechmea aquilega* (Salisb.) Griseb. | Ep |  |  | X | X | X | X |
|  | *Aechmea araenosa* L.B.Sm. | Ep |  | X | X |  |  |  |
|  | *Aechmea atrovittata* Leme & J.A.Siqueira | Ep |  | X | X |  |  |  |
|  | *Aechmea azurea* L.B.Sm. | Ep |  | X | X |  |  |  |
|  | *Aechmea bambusoides* L.B.Sm. & Reitz | Ep |  | X | X |  |  |  |
|  | *Aechmea bicolor* L.B.Sm. | Ep |  | X | X |  |  |  |
|  | *Aechmea blanchetiana* (Baker) L.B.Sm. | Ep |  | X | X |  |  |  |
|  | *Aechmea blumenavii* Reitz | Ep |  | X | X |  |  |  |
|  | *Aechmea bocainensis* E.Pereira & Leme | Ep |  | X | X |  |  |  |
|  | *Aechmea bromeliifolia* (Rudge) Baker | Ep |  |  | X | X | X | X |
|  | *Aechmea bruggeri* Leme | Ep |  | X | X |  |  |  |
|  | *Aechmea burlemarxii* E.Pereira | Ep |  | X | X |  |  |  |
|  | *Aechmea caesia* E.Morren ex Baker | Ep |  | X | X |  |  |  |
|  | *Aechmea calyculata* (E.Morren) Baker | Ep |  |  | X |  |  |  |
|  | *Aechmea canaliculata* Leme & H.Luther | Ep |  | X | X |  |  |  |
|  | *Aechmea candida* E.Morren ex Baker | Ep |  | X | X |  |  |  |
|  | *Aechmea capixabae* L.B.Sm. | Ep |  | X | X |  |  |  |
|  | *Aechmea cariocae* L.B.Sm. | Ep | EN | X | X |  |  |  |
|  | *Aechmea carvalhoi* E.Pereira & Leme | Ep |  | X | X |  |  |  |
|  | *Aechmea castanea* L.B.Sm. | Ep | EN | X | X |  |  |  |
|  | *Aechmea castelnavii* Baker | Ep |  |  | X | X |  |  |
|  | *Aechmea catendensis* J.A.Siqueira & Leme | Ep |  | X | X |  |  |  |
|  | *Aechmea caudata* Lindm. | Ep |  | X | X |  |  |  |
|  | *Aechmea cephaloides* J.A.Siqueira & Leme | Ep |  | X | X |  |  |  |
|  | *Aechmea chrysocoma* Baker | Ep |  | X | X |  |  |  |
|  | *Aechmea coelestis* (K.Koch) E.Morren | Ep |  | X | X |  |  |  |
|  | *Aechmea comata* (Gaudich.) Baker | Ep |  | X | X |  |  |  |
|  | *Aechmea conifera* L.B.Sm. | Ep |  | X | X |  |  |  |
|  | *Aechmea correia-araujoi* E.Pereira & Moutinho | Ep |  | X | X |  |  |  |
|  | *Aechmea costantinii* (Mez) L.B.Sm. | Ep |  | X | X |  |  |  |
|  | *Aechmea curranii* (L.B.Sm.) L.B.Sm. & M.A.Spencer | Ep |  | X | X |  |  |  |
|  | *Aechmea cylindrata* Lindm. | Ep |  | X | X |  |  |  |
|  | *Aechmea dealbata* E.Morren ex Baker | Ep |  | X | X |  |  |  |
|  | *Aechmea depressa* L.B.Sm. | Ep | EN | X | X |  |  |  |
|  | *Aechmea digitata* L.B.Sm. & R.W.Read | Ep |  | X | X |  |  |  |
|  | *Aechmea disjuncta* (L.B.Sm.) Leme & J.A.Siqueira | Ep |  | X | X |  |  |  |
|  | *Aechmea distichantha* Lem. | Ep |  |  | X |  | X |  |
|  | *Aechmea emmerichiae* Leme | Ep |  | X | X |  |  |  |
|  | *Aechmea eurycorymbus* Harms | Ep |  | X | X |  |  | X |
|  | *Aechmea farinosa* (Regel) L.B.Sm. | Ep |  | X | X |  |  |  |
|  | *Aechmea fasciata* (Lindl.) Baker | Ep |  | X | X |  |  |  |
|  | *Aechmea flavorosea* E.Pereira | Ep |  | X | X |  |  |  |
|  | *Aechmea floribunda* Mart. ex Schult. & Schult.f. | Ep |  | X | X |  |  |  |
|  | *Aechmea fosteriana* L.B.Sm. | Ep | EN | X | X |  |  |  |
|  | *Aechmea fraudulosa* Mez | Ep |  | X | X |  |  |  |
|  | *Aechmea froesii* (L.B.Sm.) Leme & J.A.Siqueira | Ep |  | X | X |  |  |  |
|  | *Aechmea fulgens* Brongn. | Ep |  | X | X |  |  |  |
|  | *Aechmea gamosepala* Wittm. | Ep |  | X | X |  |  |  |
|  | *Aechmea gracilis* Lindm. | Ep |  | X | X |  |  |  |
|  | *Aechmea grazielae* Martinelli & Leme | Ep |  | X | X |  |  |  |
|  | *Aechmea guarapariensis* E.Pereira & Leme | Ep |  | X | X |  |  |  |
|  | *Aechmea guaratingensis* Leme & Kollmann | Ep |  | X | X |  |  |  |
|  | *Aechmea guaratubensis* E.Pereira | Ep |  | X | X |  |  |  |
|  | *Aechmea gurkeniana* E.Pereira & Moutinho | Ep |  | X | X |  |  |  |
|  | *Aechmea gustavoi* J.A.Siqueira & Leme | Ep | CR | X | X |  |  |  |
|  | *Aechmea heterosepala* Leme | Ep |  | X | X |  |  |  |
|  | *Aechmea incompta* Leme & H.Luther | Ep |  | X | X |  |  |  |
|  | *Aechmea kertesziae* Reitz | Ep | EN | X | X |  |  |  |
|  | *Aechmea kleinii* Reitz | Ep | EN | X | X |  |  |  |
|  | *Aechmea lactifera* Leme & J.A.Siqueira | Ep |  | X | X |  |  |  |
|  | *Aechmea laevigata* Leme | Ep |  | X | X |  |  |  |
|  | *Aechmea lamarchei* Mez | Ep |  | X | X |  | X |  |
|  | *Aechmea lanata* (L.B.Sm.) L.B.Sm. & M.A.Spencer | Ep |  | X | X |  |  |  |
|  | *Aechmea leonardkentiana* H.Luther & Leme | Ep |  | X | X |  |  |  |
|  | *Aechmea leppardii* Philcox | Ep |  | X | X |  |  |  |
|  | *Aechmea leptantha* (Harms) Leme & J.A.Siqueira | Ep |  | X | X |  |  | X |
|  | *Aechmea limai* Leme | Ep |  | X | X |  |  |  |
|  | *Aechmea linharesii* Leme | Ep |  | X | X |  |  |  |
|  | *Aechmea lymanii* W.Weber | Ep |  | X | X |  |  |  |
|  | *Aechmea maasii* Gouda & W.Till | Ep |  | X | X |  |  |  |
|  | *Aechmea macrochlamys* L.B.Sm. | Ep | EN | X | X |  |  |  |
|  | *Aechmea marauensis* Leme | Ep |  | X | X |  |  |  |
|  | *Aechmea mertensii* (G.Meyer) Schult. & Schult.f. | Ep |  |  | X | X |  |  |
|  | *Aechmea miniata* Beer | Ep |  | X | X |  |  |  |
|  | *Aechmea mira* Leme & H.Luther | Ep |  | X | X |  |  |  |
|  | *Aechmea multiflora* L.B.Sm. | Ep |  | X | X |  |  |  |
|  | *Aechmea muricata* (Arruda) L.B.Sm. | Ep | EN | X | X |  |  |  |
|  | *Aechmea mutica* L.B.Sm. | Ep | EN | X | X |  |  |  |
|  | *Aechmea nudicaulis* (L.) Griseb. | Ep |  |  | X |  | X |  |
|  | *Aechmea organensis* Wawra | Ep |  | X | X |  |  |  |
|  | *Aechmea orlandiana* L.B.Sm. | Ep | CR | X | X |  |  |  |
|  | *Aechmea ornata* Baker | Ep |  | X | X |  |  |  |
|  | *Aechmea paratiensis* Leme & Fraga | Ep |  | X | X |  |  |  |
|  | *Aechmea patentissima* (Mart. ex Schult. & Schult.f.) Baker | Ep |  |  | X | X |  |  |
|  | *Aechmea pectinata* Baker | Ep |  | X | X |  |  |  |
|  | *Aechmea pedicellata* Leme & H.Luther | Ep |  | X | X |  |  |  |
|  | *Aechmea perforata* L.B.Sm. | Ep |  | X | X |  |  |  |
|  | *Aechmea pernambucentris* J.A.Siqueira & Leme | Ep |  | X | X |  |  |  |
|  | *Aechmea phanerophlebia* Baker | Ep |  | X | X |  | X |  |
|  | *Aechmea pimentivelosoi* Reitz | Ep |  | X | X |  |  |  |
|  | *Aechmea pineliana* (Brong. ex Planch.) Baker | Ep |  | X | X |  |  |  |
|  | *Aechmea podantha* L.B.Sm. | Ep |  | X | X |  |  |  |
|  | *Aechmea prava* E.Pereira | Ep |  | X | X |  |  |  |
|  | *Aechmea pseudonudicaulis* Leme | Ep |  | X | X |  |  |  |
|  | *Aechmea purpureorosea* (Hook.) Wawra | Ep |  | X | X |  |  |  |
|  | *Aechmea racinae* L.B.Sm. | Ep |  | X | X |  |  |  |
|  | *Aechmea ramosa* Mart. ex Schult. & Schult.f. | Ep |  | X | X |  |  |  |
|  | *Aechmea recurvata* (Klotzsch) L.B.Sm. | Ep |  |  | X |  |  |  |
|  | *Aechmea recurvipetala* Leme & L.Kollmann | Ep |  | X | X |  |  |  |
|  | *Aechmea robertoseidelii* E.Pereira | Ep |  | X | X |  |  |  |
|  | *Aechmea rubrolilacina* Leme | Ep |  | X | X |  |  |  |
|  | *Aechmea saxicola* L.B.Sm. | Ep |  | X | X |  |  |  |
|  | *Aechmea serragrandensis* Leme & J.A.Siqueira | Ep |  | X | X |  |  |  |
|  | *Aechmea sphaerocephala* Baker | Ep | EN | X | X |  |  |  |
|  | *Aechmea squarrosa* Baker | Ep |  | X | X |  |  |  |
|  | *Aechmea subintegerrima* (Philcox) Leme | Ep |  | X | X |  |  |  |
|  | *Aechmea sulbahianensis* Leme et al. | Ep |  | X | X |  |  |  |
|  | *Aechmea tentaculifera* Leme et al. | Ep |  | X | X |  |  |  |
|  | *Aechmea tomentosa* Mez | Ep |  | X | X |  |  |  |
|  | *Aechmea triangularis* L.B.Sm | Ep | EN | X | X |  |  |  |
|  | *Aechmea triticina* Mez | Ep |  | X | X |  |  |  |
|  | *Aechmea turbinocalyx* Mez | Ep |  | X | X |  |  |  |
|  | *Aechmea vanhoutteana* (Van Houtte) Mez | Ep | VU | X | X |  |  |  |
|  | *Aechmea victoriana* L.B.Sm. | Ep |  | X | X |  |  |  |
|  | *Aechmea viridostigma* Leme & H.Luther | Ep |  | X | X |  |  |  |
|  | *Aechmea warasii* E.Pereira | Ep |  | X | X |  |  |  |
|  | *Aechmea weilbachii* F.Didr. | Ep |  | X | X |  |  |  |
|  | *Aechmea werdermannii* Harms | Ep | EN | X | X |  |  | X |
|  | *Aechmea winkleri* Reitz | Ep | CR | X | X |  |  |  |
|  | *Aechmea wittmackiana* (Regel) Mez | Ep |  | X | X |  |  |  |
|  | *Araeococcus chlorocarpus* (Wawra) Leme & J.A.Siqueira | Ep |  | X | X |  |  |  |
|  | *Araeococcus montanus* Leme | Ep | EN | X | X |  |  |  |
|  | *Araeococcus nigropurpureus* Leme & J.A.Siqueira | Ep |  | X | X |  |  |  |
|  | *Araeococcus parviflorus* (Mart. ex. Schult. & Schult.f.) Lindm. | Ep |  | X | X |  |  |  |
|  | *Araeococcus sessiliflorus* Leme & J.A.Siqueira | Ep |  | X | X |  |  |  |
|  | *Billbergia alfonsijoannis* Reitz | Ep |  | X | X |  | X |  |
|  | *Billbergia amoena* (Lodd.) Lindl. | Ep |  | X | X |  | X | X |
|  | *Billbergia bradeana* L.B.Sm. | Ep |  | X | X |  |  |  |
|  | *Billbergia brasiliensis* L.B.Sm. | Ep | EN | X | X |  |  |  |
|  | *Billbergia castelensis* E.Pereira | Ep |  | X | X |  |  |  |
|  | *Billbergia chlorantha* L.B.Sm. | Ep |  | X | X |  |  |  |
|  | *Billbergia distachia* (Vell.) Mez | Ep |  | X | X |  | X |  |
|  | *Billbergia elegans* Mart. ex Schult. & Schult.f. | Ep |  | X | X |  | X |  |
|  | *Billbergia euphemiae* E.Morren | Ep |  | X | X |  |  |  |
|  | *Billbergia horrida* Regel | Ep |  | X | X |  |  |  |
|  | *Billbergia iridifolia* (Nees & Mart.) Lindl. | Ep |  | X | X |  |  | X |
|  | *Billbergia kautskyana* E.Pereira | Ep |  | X | X |  |  |  |
|  | *Billbergia laxiflora* L.B.Sm. | Ep |  | X | X |  |  |  |
|  | *Billbergia leptopoda* L.B.Sm | Ep |  | X | X |  |  |  |
|  | *Billbergia lymanii* E.Pereira & Leme | Ep |  | X | X |  |  |  |
|  | *Billbergia macracantha* E.Pereira | Ep |  | X | X |  |  |  |
|  | *Billbergia magnifica* Mez | Ep |  |  | X |  | X |  |
|  | *Billbergia meyeri* Mez | Ep |  |  | X |  | X |  |
|  | *Billbergia minarum* L.B.Sm. | Ep |  | X | X |  |  |  |
|  | *Billbergia morelii* Brongn. | Ep |  | X | X |  |  |  |
|  | *Billbergia nana* E.Pereira | Ep |  | X | X |  |  |  |
|  | *Billbergia nutans* H.H.Wendl. ex Regel | Ep |  |  | X |  |  |  |
|  | *Billbergia pohliana* Mez | Ep |  | X | X |  |  |  |
|  | *Billbergia porteana* Brongn. ex Beer | Ep |  |  | X |  | X | X |
|  | *Billbergia pyramidalis* (Sims) Lindl. | Ep |  | X | X |  |  |  |
|  | *Billbergia reichardtii* Wawra | Ep |  | X | X |  |  |  |
|  | *Billbergia sanderiana* E.Morren | Ep |  | X | X |  | X |  |
|  | *Billbergia saundersii* Bull | Ep |  | X | X |  |  |  |
|  | *Billbergia tweedieana* Baker | Ep |  | X | X |  |  |  |
|  | *Billbergia vittata* Brongn. | Ep |  | X | X |  | X | X |
|  | *Billbergia zebrina* (Herb.) Lindl. | Ep |  |  | X |  |  |  |
|  | *Canistropsis albiflora* (L.B.Sm) H.Luther & Leme | Ep | VU | X | X |  |  |  |
|  | *Canistropsis billbergioides* (Schult. & Schult.f.) Leme | Ep |  | X | X |  |  |  |
|  | *Canistropsis burchellii* (Baker) Leme | Ep |  | X | X |  |  |  |
|  | *Canistropsis exigua* (E.Pereira & Leme) Leme | Ep |  | X | X |  |  |  |
|  | *Canistropsis microps* (E.Morren ex Leme) Leme | Ep |  | X | X |  |  |  |
|  | *Canistropsis seidelii* (L.B.Sm. & Reitz) Leme | Ep |  | X | X |  |  |  |
|  | *Canistropsis simulans* (E.Pereira & Leme) Leme | Ep |  | X | X |  |  |  |
|  | *Canistrum alagoanum* Leme & J.A.Siqueira | Ep | EN | X | X |  |  |  |
|  | *Canistrum aurantiacum* E.Morren | Ep | EN | X | X |  |  | X |
|  | *Canistrum auratum* Leme | Ep |  | X | X |  |  |  |
|  | *Canistrum camacaense* Martinelli & Leme | Ep | EN | X | X |  |  |  |
|  | *Canistrum fosterianum* L.B.Sm. | Ep | CR | X | X |  |  |  |
|  | *Canistrum guzmanioides* L.B.Sm. | Ep | EN | X | X |  |  |  |
|  | *Canistrum montanum* Leme | Ep | EN | X | X |  |  |  |
|  | *Canistrum pickelii* (A.Lima & L.B.Sm.) Leme & J.A.Siqueira | Ep | VU | X | X |  |  | X |
|  | *Canistrum sandrae* Leme | Ep |  | X | X |  |  |  |
|  | *Canistrum seidelianum* W.Weber | Ep |  | X | X |  |  |  |
|  | *Canistrum triangulare* L.B.Sm. & Reitz | Ep | EN | X | X |  |  |  |
|  | *Catopsis berteroniana* (Schult. & Schult.f.) Mez | Ep |  |  | X |  |  |  |
|  | *Catopsis sessiliflora* (Ruiz & Pav.) Mez | Ep |  |  | X | X |  |  |
|  | *Edmundoa ambigua* (Wand. & Leme) Leme | Ep |  | X | X |  |  |  |
|  | *Edmundoa lindenii* (Regel) Leme | Ep |  | X | X |  |  |  |
|  | *Edmundoa perplexa* (L.B.Sm.) Leme & J.A.Siqueira | Ep |  | X | X |  |  |  |
|  | *Guzmania lingulata* (L.) Mez | Ep |  |  | X | X |  |  |
|  | *Guzmania monostachia* (L.) Rusby ex Mez | Ep | VU |  | X |  |  |  |
|  | *Guzmania sanguinea* (André) André ex Mez | Ep | EN |  | X |  |  |  |
|  | *Hohenbergia augusta* (Vell.) Morren | Ep |  | X | X |  |  |  |
|  | *Hohenbergia barbarespina* Leme & Fraga | Ep |  | X | X |  |  |  |
|  | *Hohenbergia belemii* L.B.Sm. & R.W.Read | Ep |  | X | X |  |  |  |
|  | *Hohenbergia blanchetii* (Baker) E.Morren ex Mez | Ep |  | X | X |  |  |  |
|  | *Hohenbergia brachycephala* L.B.Sm. | Ep |  | X | X |  |  |  |
|  | *Hohenbergia burlemarxii* Leme & W.Till | Ep |  | X | X |  |  |  |
|  | *Hohenbergia capitata* Schult. & Schult.f. | Ep |  | X | X |  |  |  |
|  | *Hohenbergia castellanosii* L.B.Sm. & R.W.Read | Ep | EN | X | X |  |  |  |
|  | *Hohenbergia correia-araujoi* E.Pereira & Moutinho | Ep | CR | X | X |  |  |  |
|  | *Hohenbergia edmundoi* L.B.Sm. & R.W.Read | Ep |  | X | X |  |  |  |
|  | *Hohenbergia flava* Leme & C.C.Paula | Ep |  | X | X |  |  |  |
|  | *Hohenbergia hatschbachii* Leme | Ep |  | X | X |  |  |  |
|  | *Hohenbergia itamarajuensis* Leme & Baracho | Ep |  | X | X |  |  |  |
|  | *Hohenbergia lanata* E.Pereira & Moutinho | Ep |  | X | X |  |  |  |
|  | *Hohenbergia lemei* H.Luther & K.Norton | Ep |  | X | X |  |  |  |
|  | *Hohenbergia loredanoana* Leme & L.Kollmann | Ep |  | X | X |  |  |  |
|  | *Hohenbergia minor* L.B.Sm. | Ep |  | X | X |  |  |  |
|  | *Hohenbergia pabstii* L.B.Sm. & R.W.Read | Ep |  | X | X |  |  |  |
|  | *Hohenbergia ramageana* Mez | Ep |  | X | X |  |  |  |
|  | *Hohenbergia ridleyi* (Baker) Mez | Ep |  | X | X |  |  |  |
|  | *Hohenbergia rosea* L.B.Sm. & R.W.Read | Ep |  | X | X |  |  | X |
|  | *Hohenbergia salzmannii* (Baker) E.Morren ex Mez | Ep |  | X | X |  |  |  |
|  | *Hohenbergia stellata* Schult. & Schult.f. | Ep |  |  | X |  |  | X |
|  | *Lymania alvimii* (L.B.Sm. & R.W.Read) R.W.Read | Ep | EN | X | X |  |  |  |
|  | *Lymania azurea* Leme | Ep | EN | X | X |  |  |  |
|  | *Lymania brachycaulis* (E.Morren ex Baker) L.F.Sousa | Ep | EN | X | X |  |  |  |
|  | *Lymania corallina* (Brong ex Beer) R.W.Read | Ep | EN | X | X |  |  |  |
|  | *Lymania globosa* Leme | Ep | EN | X | X |  |  |  |
|  | *Lymania languida* Leme | Ep |  | X | X |  |  |  |
|  | *Lymania smithii* R.W.Read | Ep |  | X | X |  |  |  |
|  | *Lymania spiculata* Leme & Forzza | Ep | CR | X | X |  |  |  |
|  | *Neoregelia abendrothae* L.B.Sm. | Ep |  | X | X |  |  |  |
|  | *Neoregelia ampullacea* (E.Morren) L.B.Sm. | Ep |  | X | X |  |  |  |
|  | *Neoregelia angustibracetoala* E.Pereira & Leme | Ep | CR | X | X |  |  |  |
|  | *Neoregelia angustifolia* E.Pereira | Ep |  | X | X |  |  |  |
|  | *Neoregelia atroviridifolia* W.Weber | Ep |  | X | X |  |  |  |
|  | *Neoregelia azevedoi* Leme | Ep |  | X | X |  |  |  |
|  | *Neoregelia binotii* (Antoine) L.B.Sm. | Ep |  | X | X |  |  |  |
|  | *Neoregelia bragarum* (E.Pereira & L.B.Sm.) Leme | Ep |  | X | X |  |  |  |
|  | *Neoregelia brownii* Leme | Ep | CR | X | X |  |  |  |
|  | *Neoregelia camorimiana* E.Pereira & I.A.Penna | Ep |  | X | X |  |  |  |
|  | *Neoregelia capixaba* E.Pereira & Leme | Ep |  | X | X |  |  |  |
|  | *Neoregelia carcharodon* (Baker) L.B.Sm. | Ep |  | X | X |  |  |  |
|  | *Neoregelia carinata* Leme | Ep |  | X | X |  |  |  |
|  | *Neoregelia carolinae* (Beer) L.B.Sm. | Ep |  | X | X |  |  |  |
|  | *Neoregelia chlorosticta* (Baker) L.B.Sm. | Ep |  | X | X |  |  |  |
|  | *Neoregelia coimbrae* E.Pereira & Leme | Ep |  | X | X |  |  |  |
|  | *Neoregelia compacta* (Mez) L.B.Sm. | Ep |  | X | X |  |  |  |
|  | *Neoregelia concentrica* (Vell.) L.B.Sm. | Ep |  | X | X |  |  |  |
|  | *Neoregelia coriacea* (Antoine) L.B.Sm. | Ep |  | X | X |  |  |  |
|  | *Neoregelia correia-araujoi* E.Pereira & I.A.Penna | Ep |  | X | X |  |  |  |
|  | *Neoregelia crispata* Leme | Ep |  | X | X |  |  |  |
|  | *Neoregelia cruenta* (R.Graham) L.B.Sm. | Ep |  | X | X |  |  |  |
|  | *Neoregelia cyanea* (Beer) L.B.Sm. | Ep |  | X | X |  |  |  |
|  | *Neoregelia dayvidiana* Leme & A. P. Fontana | Ep |  | X | X |  |  |  |
|  | *Neoregelia diversifolia* E.Pereira | Ep |  | X | X |  |  |  |
|  | *Neoregelia doeringiana* L.B.Sm. | Ep |  | X | X |  |  |  |
|  | *Neoregelia dungsiana* E.Pereira | Ep |  | X | X |  |  |  |
|  | *Neoregelia eltoniana* W.Weber | Ep |  | X | X |  |  |  |
|  | *Neoregelia farinosa* (Ule) L.B.Sm. | Ep |  | X | X |  |  |  |
|  | *Neoregelia fluminensis* L.B.Sm. | Ep |  | X | X |  |  |  |
|  | *Neoregelia fosteriana* L.B.Sm. | Ep |  | X | X |  |  |  |
|  | *Neoregelia gavionensis* Martinelli & Leme | Ep |  | X | X |  |  |  |
|  | *Neoregelia gigas* Leme & L.Kollmann | Ep |  | X | X |  |  |  |
|  | *Neoregelia guttata* Leme | Ep |  | X | X |  |  |  |
|  | *Neoregelia hoehneana* L.B.Sm. | Ep | EN | X | X |  |  |  |
|  | *Neoregelia ibitipocensis* (Leme) Leme | Ep |  | X | X |  |  |  |
|  | *Neoregelia ilhana* Leme | Ep |  | X | X |  |  |  |
|  | *Neoregelia indecora* (Mez) L.B.Sm. | Ep |  | X | X |  |  |  |
|  | *Neoregelia inexspectata* Leme | Ep | EN | X | X |  |  |  |
|  | *Neoregelia johannis* (Carriere) L.B.Sm. | Ep |  | X | X |  |  |  |
|  | *Neoregelia kautskyi* E.Pereira | Ep |  | X | X |  |  |  |
|  | *Neoregelia kerryi* Leme | Ep |  | X | X |  |  |  |
|  | *Neoregelia kuhlmannii* L.B.Sm. | Ep |  | X | X |  |  |  |
|  | *Neoregelia lactea* H.Luther & Leme | Ep |  | X | X |  |  |  |
|  | *Neoregelia laevis* (Mez) L.B.Sm. | Ep |  | X | X |  |  |  |
|  | *Neoregelia leprosa* L.B.Sm. | Ep | VU | X | X |  | X |  |
|  | *Neoregelia leucophloea* (Baker) L.B.Sm. | Ep |  | X | X |  |  |  |
|  | *Neoregelia lilliputiana* E.Pereira | Ep |  | X | X |  |  |  |
|  | *Neoregelia lillyae* W.Weber | Ep |  | X | X |  |  |  |
|  | *Neoregelia longipedicellata* Leme | Ep |  | X | X |  |  |  |
|  | *Neoregelia longisepala* E.Pereira & I.A.Penna | Ep |  | X | X |  |  |  |
|  | *Neoregelia lymaniana* R.Braga & D.Sucre | Ep |  | X | X |  |  |  |
|  | *Neoregelia macahensis* (Ule) L.B.Sm. | Ep |  | X | X |  |  |  |
|  | *Neoregelia macrosepala* L.B.Sm. | Ep |  | X | X |  |  |  |
|  | *Neoregelia maculata* L.B.Sm. | Ep |  | X | X |  |  |  |
|  | *Neoregelia macwilliamsii* L.B.Sm. | Ep |  | X | X |  |  |  |
|  | *Neoregelia magdalenae* L.B.Sm. & Reitz | Ep |  | X | X |  |  |  |
|  | *Neoregelia marmorata* (Baker) L.B.Sm. | Ep |  | X | X |  |  |  |
|  | *Neoregelia martinellii*  W.Weber | Ep |  | X | X |  |  |  |
|  | *Neoregelia melanodonta* L.B.Sm. | Ep |  | X | X |  |  |  |
|  | *Neoregelia menescalii*  Leme | Ep | EN | X | X |  |  |  |
|  | *Neoregelia nevaresii* Leme & H.Luther | Ep |  | X | X |  |  |  |
|  | *Neoregelia nivea*  Leme | Ep |  | X | X |  |  |  |
|  | *Neoregelia odorata* Leme | Ep |  | X | X |  |  |  |
|  | *Neoregelia olens* (Hook.f.) L.B.Sm. | Ep |  | X | X |  |  |  |
|  | *Neoregelia oligantha* L.B.Sm. | Ep | VU | X | X |  |  |  |
|  | *Neoregelia paratiensis* Leme | Ep |  | X | X |  |  |  |
|  | *Neoregelia pascoaliana* L.B.Sm. | Ep |  | X | X |  |  |  |
|  | *Neoregelia pauciflora* L.B.Sm. | Ep |  | X | X |  |  |  |
|  | *Neoregelia paulistana* E.Pereira | Ep | EN | X | X |  |  |  |
|  | *Neoregelia pernambucana* Leme & J.A.Siqueira | Ep | EN | X | X |  |  |  |
|  | *Neoregelia petropolitana* Leme | Ep |  | X | X |  |  |  |
|  | *Neoregelia pineliana* (Lem.) L.B.Sm. | Ep |  | X | X |  |  |  |
|  | *Neoregelia pontualii* Leme | Ep |  | X | X |  |  |  |
|  | *Neoregelia princeps* (Baker) L.B.Sm. | Ep |  | X | X |  |  |  |
|  | *Neoregelia punctatissima* (Ruschi) Ruschi | Ep |  | X | X |  |  |  |
|  | *Neoregelia richteri* W.Weber | Ep |  | X | X |  |  |  |
|  | *Neoregelia roethii* W.Weber | Ep |  | X | X |  |  |  |
|  | *Neoregelia rothinessa* Leme, H.Luther & W.Till | Ep |  | X | X |  |  |  |
|  | *Neoregelia rubrifolia* Leme & B.R.Silva | Ep |  | X | X |  |  |  |
|  | *Neoregelia rubrovittata* Leme | Ep |  | X | X |  |  |  |
|  | *Neoregelia ruschii* Leme & B.R.Silva | Ep | EN | X | X |  |  |  |
|  | *Neoregelia sanguinea* Leme | Ep | EN | X | X |  |  |  |
|  | *Neoregelia sapiatibensis* E.Pereira & I.A.Penna | Ep |  | X | X |  |  |  |
|  | *Neoregelia sarmentosa* (Regel) L.B.Sm. | Ep |  | X | X |  |  |  |
|  | *Neoregelia schubertii* Roth | Ep |  | X | X |  |  |  |
|  | *Neoregelia seideliana* L.B.Sm. & Reitz | Ep |  | X | X |  |  |  |
|  | *Neoregelia silvomontana* Leme & J.A.Siqueira | Ep |  | X | X |  |  |  |
|  | *Neoregelia simulans* L.B.Sm. | Ep |  | X | X |  |  |  |
|  | *Neoregelia smithii* W.Weber | Ep |  | X | X |  |  |  |
|  | *Neoregelia spectabilis* (T.Moore) L.B.Sm. | Ep |  | X | X |  |  |  |
|  | *Neoregelia tenebrosa* Leme | Ep |  | X | X |  |  |  |
|  | *Neoregelia tigrina* (Ruschi) Ruschi | Ep |  | X | X |  |  |  |
|  | *Neoregelia tristis* (Beer) L.B.Sm. | Ep |  | X | X |  |  |  |
|  | *Neoregelia uleana* L.B.Sm. | Ep |  | X | X |  |  |  |
|  | *Neoregelia viridolineata* Leme | Ep |  | X | X |  |  |  |
|  | *Neoregelia viridovinosa* Leme & L.Kollmann | Ep |  | X | X |  |  |  |
|  | *Neoregelia wilsoniana* M.B.Foster | Ep |  | X | X |  |  |  |
|  | *Neoregelia zalawskyi* E.Pereira & Leme | Ep |  | X | X |  |  |  |
|  | *Nidularium albiflorum* (L.B.Sm.) Leme | Ep |  | X | X |  |  |  |
|  | *Nidularium alvimii* W.Weber | Ep |  | X | X |  |  |  |
|  | *Nidularium amazonicum* (Baker) Linden & E.Morren ex Lindm. | Ep |  | X | X |  |  |  |
|  | *Nidularium amorimii* Leme | Ep |  | X | X |  |  |  |
|  | *Nidularium angustibracteatum* Leme | Ep |  | X | X |  |  |  |
|  | *Nidularium angustifolium* Ule | Ep |  | X | X |  |  |  |
|  | *Nidularium antoineanum* Wawra | Ep |  | X | X |  |  |  |
|  | *Nidularium apiculatum* L.B.Sm. | Ep |  | X | X |  |  |  |
|  | *Nidularium azureum* (L.B.Sm.) Leme | Ep | CR | X | X |  |  |  |
|  | *Nidularium bicolor* (E.Pereira) Leme | Ep |  | X | X |  |  |  |
|  | *Nidularium bocainense* Leme | Ep | EN | X | X |  |  |  |
|  | *Nidularium campo-alegrense* Leme | Ep |  | X | X |  |  |  |
|  | *Nidularium cariacicaense* (W.Weber) Leme | Ep |  | X | X |  |  |  |
|  | *Nidularium catarinense* Leme | Ep |  | X | X |  |  |  |
|  | *Nidularium corallinum* (Leme) Leme | Ep | EN | X | X |  |  |  |
|  | *Nidularium espiritosantense* Leme | Ep |  | X | X |  |  |  |
|  | *Nidularium ferdinando-coburgii* Wawra | Ep |  | X | X |  |  |  |
|  | *Nidularium fradense* Leme | Ep |  | X | X |  |  |  |
|  | *Nidularium fulgens* Lem. | Ep |  | X | X |  |  |  |
|  | *Nidularium innocentii* Lem. | Ep |  | X | X |  |  |  |
|  | *Nidularium itaitiaiae* L.B.Sm. | Ep | EN | X | X |  |  |  |
|  | *Nidularium jonesianum* Leme | Ep | EN | X | X |  |  |  |
|  | *Nidularium kautskyanum* Leme | Ep | EN | X | X |  |  |  |
|  | *Nidularium longiflorum* Ule | Ep |  | X | X |  |  |  |
|  | *Nidularium mangaratibense* Leme | Ep | CR | X | X |  |  |  |
|  | *Nidularium marigoi* Leme | Ep |  | X | X |  |  |  |
|  | *Nidularium meeanum* Leme *et al.* | Ep |  | X | X |  |  |  |
|  | *Nidularium organense* Leme | Ep | EN | X | X |  |  |  |
|  | *Nidularium piciguabense* Leme | Ep |  | X | X |  |  |  |
|  | *Nidularium procerum* Lindm. | Ep |  | X | X |  |  |  |
|  | *Nidularium purpureum* Beer | Ep |  | X | X |  |  |  |
|  | *Nidularium rolfianum* Leme | Ep |  | X | X |  |  |  |
|  | *Nidularium rosulatum* Ule | Ep | VU | X | X |  |  |  |
|  | *Nidularium rutilans* E.Morren | Ep |  | X | X |  |  |  |
|  | *Nidularium scheremetiewii* Regel | Ep |  | X | X |  |  |  |
|  | *Nidularium serratum* Leme | Ep | VU | X | X |  |  |  |
|  | *Nidularium urticulosum* Ule | Ep | VU | X | X |  |  |  |
|  | *Nidularium viridipetalum* Leme | Ep |  | X | X |  |  |  |
|  | *Portea alatisepala* Philcox | Ep | VU | X | X |  |  |  |
|  | *Portea filifera* L.B.Sm. | Ep |  | X | X |  |  |  |
|  | *Portea fosteriana* L.B.Sm. | Ep | EN | X | X |  |  |  |
|  | *Portea grandiflora* Philcox | Ep | VU | X | X |  |  |  |
|  | *Portea kermesina* H.Luther | Ep | EN | X | X |  |  |  |
|  | *Portea nana* Leme & H.Luther | Ep | EN | X | X |  |  |  |
|  | *Portea petropolitana* (Wawra) Mez | Ep |  | X | X |  |  |  |
|  | *Portea silveirae* Mez | Ep |  | X | X |  |  |  |
|  | *Quesnelia alborosea* A.F.Costa & T.Fontoura | Ep |  | X | X |  |  |  |
|  | *Quesnelia alvimii* Leme | Ep |  | X | X |  |  |  |
|  | *Quesnelia arvensis* (Vell.) Mez | Ep |  | X | X |  |  |  |
|  | *Quesnelia augustocoburgii* Wawra | Ep |  | X | X |  |  |  |
|  | *Quesnelia clavata* Amorim & Leme | Ep |  | X | X |  |  |  |
|  | *Quesnelia conquistensis* Leme | Ep |  | X | X |  |  |  |
|  | *Quesnelia dubia* Leme | Ep |  | X | X |  |  |  |
|  | *Quesnelia edmundoi* L.B.Sm. | Ep |  | X | X |  |  |  |
|  | *Quesnelia humilis* Mez | Ep |  | X | X |  |  |  |
|  | *Quesnelia imbricata* L.B.Sm. | Ep |  | X | X |  |  |  |
|  | *Quesnelia indecora* Mez | Ep |  | X | X |  |  |  |
|  | *Quesnelia kautsky* C.Vieira | Ep | VU | X | X |  |  |  |
|  | *Quesnelia koltesii* Amorim & Leme | Ep |  | X | X |  |  |  |
|  | *Quesnelia lateralis* Wawra | Ep |  | X | X |  |  |  |
|  | *Quesnelia liboniana* (De Jongle) Mez | Ep |  | X | X |  |  |  |
|  | *Quesnelia marmorata* (Lem.) R.W.Read | Ep |  | X | X |  |  |  |
|  | *Quesnelia quesneliana* (Brongn.) L.B.Sm. | Ep |  | X | X |  |  |  |
|  | *Quesnelia seideliana* L.B.Sm. | Ep | EN | X | X |  |  |  |
|  | *Quesnelia strobilispica* Wawra | Ep |  | X | X |  |  |  |
|  | *Quesnelia testudo* Lindm. | Ep |  | X | X |  |  |  |
|  | *Quesnelia tubifolia* Leme & L.Kollmann | Ep |  | X | X |  |  |  |
|  | *Quesnelia violacea* Wand. & S.L.Proença | Ep |  | X | X |  |  |  |
|  | *Racinaea aerisincola* (Mez) M.A.Spencer & L.B.Sm. | Ep |  | X | X |  |  |  |
|  | *Racinaea domingosmartinsis* (Rauh) J.R.Grant | Ep |  | X | X |  |  |  |
|  | *Racinaea spiculosa* (Griseb) M.A.Spencer & L.B.Sm. | Ep |  |  | X |  |  |  |
|  | *Ronnbergia brasiliensis* E.Pereira & I.A.Penna | Ep |  | X | X |  |  |  |
|  | *Ronnbergia carvalhoi* Martinelli & Leme | Ep |  | X | X |  |  |  |
|  | *Ronnbergia neoregelioides* Leme | Ep |  | X | X |  |  |  |
|  | *Ronnbergia silvana* Leme | Ep |  | X | X |  |  |  |
|  | *Tillandsia aeranthos* (Loisel.) L.B.Sm. | Ep |  |  | X |  |  |  |
|  | *Tillandsia afonsoana* T.Strehl | Ep | CR | X | X |  |  |  |
|  | *Tillandsia araujei* Mez | Ep | EN | X | X |  |  |  |
|  | *Tillandsia arequitae* (André) André ex Mez | Ep |  |  | X |  |  |  |
|  | *Tillandsia bergerii* Mez | Ep |  |  | X |  |  |  |
|  | *Tillandsia bracyphylla* Baker | Ep | EN | X | X |  |  |  |
|  | *Tillandsia bulbosa* Hook.f. | Ep |  |  | X | X |  |  |
|  | *Tillandsia chapeuensis* Rauh | Ep |  | X | X |  | X | X |
|  | *Tillandsia crocata* (E.Morren) Baker | Ep | EN |  | X |  |  |  |
|  | *Tillandsia didisticha* (E.Morren) Baker | Ep |  |  | X |  | X |  |
|  | *Tillandsia dura* Baker | Ep |  | X | X |  |  |  |
|  | *Tillandsia eltoniana* E.Pereira | Ep |  | X | X |  |  |  |
|  | *Tillandsia gardneri* Lindl. | Ep |  |  | X |  | X | X |
|  | *Tillandsia geminiflora* Brongn. | Ep |  |  | X |  | X | X |
|  | *Tillandsia globosa* Wawra | Ep |  |  | X |  |  |  |
|  | *Tillandsia heubergeri* Ehlers | Ep | VU | X | X |  | X | X |
|  | *Tillandsia itaubensis* T.Strehl | Ep |  | X | X |  |  |  |
|  | *Tillandsia jonesii* T.Strehl | Ep | CR | X | X |  |  |  |
|  | *Tillandsia juncea* (Ruiz & Pav.) Poir. | Ep |  |  | X |  |  | X |
|  | *Tillandsia kautskyi* E.Pereira | Ep | VU | X | X |  |  |  |
|  | *Tillandsia kegeliana* Mez | Ep |  |  | X |  |  |  |
|  | *Tillandsia leonamiana*  E.Pereira | Ep |  | X | X |  |  |  |
|  | *Tillandsia linearis* Vell. | Ep |  | X | X |  | X |  |
|  | *Tillandsia loliacea* Mart. ex Schult & Schult.f. | Ep |  |  | X |  | X | X |
|  | *Tillandsia lorentziana* Griseb. | Ep |  |  | X |  | X |  |
|  | *Tillandsia mallemontii* Glaz. ex Mez | Ep |  | X | X |  | X | X |
|  | *Tillandsia montana* Reitz | Ep |  | X | X |  |  |  |
|  | *Tillandsia paraensis* Mez | Ep |  |  | X | X |  |  |
|  | *Tillandsia pohliana* Mez | Ep |  |  | X |  | X | X |
|  | *Tillandsia polystachia* (L.) L. | Ep |  |  | X |  | X | X |
|  | *Tillandsia pruinosa* Sw. | Ep |  |  | X | X |  |  |
|  | *Tillandsia recurvata* (L.) L. | Ep |  |  | X |  | X | X |
|  | *Tillandsia recurvifolia* Hook.f. | Ep |  | X | X |  |  |  |
|  | *Tillandsia rohdenardinii* T.Strehl | Ep |  | X | X |  |  |  |
|  | *Tillandsia roseiflora* Ehlers & W.Weber | Ep |  | X | X |  |  |  |
|  | *Tillandsia seideliana* E.Pereira | Ep |  | X | X |  |  |  |
|  | *Tillandsia sprengeliana* Klotzsch ex Mez | Ep |  | X | X |  | X |  |
|  | *Tillandsia streptocarpa* Baker | Ep |  |  | X |  | X | X |
|  | *Tillandsia stricta* Sol. | Ep |  |  | X |  | X | X |
|  | *Tillandsia tenuifolia* L. | Ep |  |  | X | X | X | X |
|  | *Tillandsia toropiensis* Rauh | Ep |  | X | X |  |  |  |
|  | *Tillandsia tricholepis* Baker | Ep |  |  | X |  | X | X |
|  | *Tillandsia usneoides* (L.) L. | Ep |  |  | X |  | X | X |
|  | *Tillandsia xiphioides* Ker-Gawler | Ep | EN |  | X |  |  |  |
|  | *Vriesea agostiniana* E.Pereira | Ep |  | X | X |  |  |  |
|  | *Vriesea altimontana* E.Pereira & Martinelli | Ep | EN | X | X |  |  |  |
|  | *Vriesea altodaserrae* L.B.Sm. | Ep |  | X | X |  |  |  |
|  | *Vriesea amadoi* Leme | Ep |  | X | X |  |  |  |
|  | *Vriesea amethystina* E.Morren | Ep | CR | X | X |  |  |  |
|  | *Vriesea arachnoidea* A.F.Costa | Ep | EN | X | X |  |  |  |
|  | *Vriesea atra* Mez | Ep |  | X | X |  |  |  |
|  | *Vriesea atrococcinea* Rauh | Ep |  | X | X |  |  |  |
|  | *Vriesea barbosae* J.A.Siqueira & Leme | Ep |  | X | X |  |  |  |
|  | *Vriesea barilletti* E.Morren | Ep |  | X | X |  |  |  |
|  | *Vriesea biguassuensis* Reitz | Ep | EN | X | X |  |  |  |
|  | *Vriesea billbergioides* E.Morren ex Mez | Ep |  | X | X |  |  |  |
|  | *Vriesea bituminosa* Wawra | Ep |  | X | X |  |  |  |
|  | *Vriesea blackburniana* Leme | Ep |  | X | X |  |  |  |
|  | *Vriesea bleherae* Roth & W.Weber | Ep | CR | X | X |  |  |  |
|  | *Vriesea breviscapa* (E.Pereira & I.A.Penna) Leme | Ep |  | X | X |  |  |  |
|  | *Vriesea brusquensis* Reitz | Ep |  | X | X |  |  |  |
|  | *Vriesea calimaniana* Leme & W.Till | Ep | CR | X | X |  |  |  |
|  | *Vriesea capixabae* Leme | Ep |  | X | X |  |  |  |
|  | *Vriesea carinata* Wawra | Ep |  | X | X |  |  |  |
|  | *Vriesea cearensis* L.B.Sm. | Ep | EN | X | X |  |  |  |
|  | *Vriesea colnagoi* E.Pereira & I.A.Penna | Ep |  | X | X |  |  |  |
|  | *Vriesea corcovadensis* (Britten) Mez | Ep |  | X | X |  |  |  |
|  | *Vriesea correia-araujoi* E.Pereira & I.A.Penna | Ep |  | X | X |  |  |  |
|  | *Vriesea debilis* Leme | Ep |  | X | X |  |  |  |
|  | *Vriesea declinata* Leme | Ep |  | X | X |  |  |  |
|  | *Vriesea delicatula* L.B.Sm. | Ep | VU | X | X |  |  |  |
|  | *Vriesea dictyographa* Leme | Ep |  | X | X |  |  |  |
|  | *Vriesea drepanocarpa* (Baker) Mez | Ep |  | X | X |  |  |  |
|  | *Vriesea duvaliana* E.Morren | Ep |  | X | X |  |  |  |
|  | *Vriesea eltoniana* E.Pereira | Ep | EN | X | X |  |  |  |
|  | *Vriesea ensiformis* (Vell.) Beer | Ep |  | X | X |  |  |  |
|  | *Vriesea erythrodactylon* E.Morren | Ep |  | X | X |  |  |  |
|  | *Vriesea fenestralis* Linden & André | Ep |  | X | X |  |  |  |
|  | *Vriesea fidelensis* Leme | Ep |  | X | X |  |  |  |
|  | *Vriesea flammea* L.B.Sm. | Ep |  | X | X |  |  |  |
|  | *Vriesea flava* A.F.Costa *et al.* | Ep |  | X | X |  |  |  |
|  | *Vriesea fluminensis* E.Pereira | Ep |  | X | X |  |  |  |
|  | *Vriesea fontourae* B.R.Silva & Leme | Ep |  | X | X |  |  |  |
|  | *Vriesea fradensis* A.Costa | Ep |  | X | X |  |  |  |
|  | *Vriesea friburgensis* Mez | Ep |  | X | X |  | X |  |
|  | *Vriesea gamba* F.J.Müll. | Ep |  | X | X |  |  |  |
|  | *Vriesea garlippiana* Leme | Ep |  | X | X |  |  |  |
|  | *Vriesea gigantea* Gaudich. | Ep |  | X | X |  |  |  |
|  | *Vriesea goniorachis* (Baker) Mez | Ep |  | X | X |  |  |  |
|  | *Vriesea gracilior* (L.B.Sm.) Leme | Ep | VU | X | X |  |  |  |
|  | *Vriesea graciliscapa* W.Weber | Ep |  | X | X |  |  |  |
|  | *Vriesea gradata* (Baker) Mez | Ep |  | X | X |  |  |  |
|  | *Vriesea grandiflora* Leme | Ep |  | X | X |  |  |  |
|  | *Vriesea guttata* Linden & André | Ep |  | X | X |  |  |  |
|  | *Vriesea heterostachys* (Baker) L.B.Sm | Ep |  | X | X |  |  |  |
|  | *Vriesea hieroglyphica* (Carriere) E.Morren | Ep |  | X | X |  |  |  |
|  | *Vriesea hoehneana* L.B.Sm. | Ep |  | X | X |  |  |  |
|  | *Vriesea hydrophora* Ule | Ep |  | X | X |  |  |  |
|  | *Vriesea incurvata* Gaudich. | Ep |  | X | X |  |  |  |
|  | *Vriesea inflata* (Wawra) Wawra | Ep |  | X | X |  |  |  |
|  | *Vriesea interrogatoria* L.B.Sm. | Ep |  | X | X |  |  |  |
|  | *Vriesea itatiaiae* Wawra | Ep |  | X | X |  |  |  |
|  | *Vriesea jonesiana* Leme | Ep |  | X | X |  |  |  |
|  | *Vriesea jonghei* (K.Koch) E.Morren | Ep |  | X | X |  |  |  |
|  | *Vriesea joyae* E.Pereira & I.A.Penna | Ep |  | X | X |  |  |  |
|  | *Vriesea kautskyana* E.Pereira & I.A.Penna | Ep | VU | X | X |  |  |  |
|  | *Vriesea lancifolia* (Baker) L.B.Sm. | Ep |  | X | X |  |  |  |
|  | *Vriesea languida* L.B.Sm. | Ep |  | X | X |  |  |  |
|  | *Vriesea leptantha* Harms | Ep | CR | X | X |  |  |  |
|  | *Vriesea lidicensis* Reitz | Ep |  | X | X |  |  |  |
|  | *Vriesea linharesiae* Leme & J.A.Siqueira | Ep |  | X | X |  |  |  |
|  | *Vriesea longicaulis* (Baker) Mez | Ep |  | X | X |  | X |  |
|  | *Vriesea longiscapa* Ule | Ep |  | X | X |  |  |  |
|  | *Vriesea longisepala* A.F.Costa | Ep |  | X | X |  |  |  |
|  | *Vriesea lubbersii* (Baker) E.Morren | Ep |  | X | X |  |  |  |
|  | *Vriesea menescalii* E.Pereira & Leme | Ep | EN | X | X |  |  |  |
|  | *Vriesea michaelii* W.Weber | Ep |  | X | X |  |  |  |
|  | *Vriesea minor* (L.B.Sm.) Leme | Ep |  | X | X |  |  |  |
|  | *Vriesea minuta* Leme | Ep |  | X | X |  |  |  |
|  | *Vriesea minutiflora* Leme | Ep |  | X | X |  |  |  |
|  | *Vriesea modesta* Mez | Ep |  | X | X |  |  |  |
|  | *Vriesea mollis* Leme | Ep |  | X | X |  |  |  |
|  | *Vriesea monacorum* L.B.Sm. | Ep | CR | X | X |  |  |  |
|  | *Vriesea morrenii* Wawra | Ep |  | X | X |  |  |  |
|  | *Vriesea muelleri* Mez | Ep |  | X | X |  |  |  |
|  | *Vriesea neoglutinosa* Mez | Ep |  | X | X |  |  |  |
|  | *Vriesea noblickii* Martinelli & Leme | Ep |  | X | X |  |  |  |
|  | *Vriesea pabstii* McWilliams & L.B.Sm. | Ep |  | X | X |  |  |  |
|  | *Vriesea paradoxa* Mez | Ep |  | X | X |  |  |  |
|  | *Vriesea paraibica* Wawra | Ep |  | X | X |  |  |  |
|  | *Vriesea paratiensis* E.Pereira | Ep |  | X | X |  |  |  |
|  | *Vriesea pardalina* Mez | Ep |  | X | X |  | X |  |
|  | *Vriesea parviflora* L.B.Sm. | Ep |  | X | X |  |  |  |
|  | *Vriesea pauciflora* Mez | Ep |  | X | X |  |  |  |
|  | *Vriesea pauperrima* E.Pereira | Ep |  | X | X |  |  |  |
|  | *Vriesea penduliflora* L.B.Sm. | Ep | EN | X | X |  |  |  |
|  | *Vriesea pereirae* L.B.Sm. | Ep |  | X | X |  |  |  |
|  | *Vriesea philippocoburgii* Wawra | Ep |  | X | X |  |  |  |
|  | *Vriesea pinottii* Reitz | Ep | EN | X | X |  |  |  |
|  | *Vriesea platynema* Gaudich. | Ep |  |  | X |  |  |  |
|  | *Vriesea platzmannii* E.Morren | Ep |  | X | X |  |  |  |
|  | *Vriesea poenulata* (Baker) E.Morren ex Mez | Ep |  | X | X |  |  |  |
|  | *Vriesea procera* (Mart. ex Schult. & Schult.f.) Wittm. | Ep |  |  | X |  | X | X |
|  | *Vriesea psittacina* (Hook.) Lindl. | Ep |  | X | X |  |  |  |
|  | *Vriesea pulchra* Leme & L.Kollmann | Ep |  | X | X |  |  |  |
|  | *Vriesea punctulata* E.Pereira & I.A.Penna | Ep |  | X | X |  |  |  |
|  | *Vriesea racinae* L.B.Sm. | Ep | EN | X | X |  |  |  |
|  | *Vriesea rafaelii* Leme | Ep |  | X | X |  |  |  |
|  | *Vriesea rastrensis* Leme | Ep |  | X | X |  |  |  |
|  | *Vriesea rectifolia* Rauh | Ep |  | X | X |  |  |  |
|  | *Vriesea recurvata* Gaudich. | Ep |  | X | X |  |  |  |
|  | *Vriesea regnellii* Mez | Ep |  | X | X |  |  |  |
|  | *Vriesea reitzii* Leme & A.F.Costa | Ep |  | X | X |  |  |  |
|  | *Vriesea repandostachys* Leme | Ep |  | X | X |  |  |  |
|  | *Vriesea revoluta* B.R.Silva & Leme | Ep |  | X | X |  |  |  |
|  | *Vriesea rhodostachys* L.B.Sm. | Ep |  | X | X |  |  |  |
|  | *Vriesea rodigasiana* E.Morren | Ep |  | X | X |  |  |  |
|  | *Vriesea rubens* J.Gomes-da-Silva & A.F.Costa | Ep |  | X | X |  |  |  |
|  | *Vriesea rubyae* E.Pereira | Ep | CR | X | X |  |  |  |
|  | *Vriesea ruschii* L.B.Sm. | Ep |  | X | X |  |  |  |
|  | *Vriesea sandrae* Leme | Ep |  | X | X |  |  |  |
|  | *Vriesea saundersii* (Carriere) E.Morren ex Mez | Ep |  | X | X |  |  |  |
|  | *Vriesea sazimae* Leme | Ep | VU | X | X |  |  |  |
|  | *Vriesea scalaris* E.Morren | Ep |  | X | X |  |  |  |
|  | *Vriesea sceptrum* Mez | Ep |  | X | X |  | X |  |
|  | *Vriesea schunkii* Leme | Ep |  | X | X |  |  |  |
|  | *Vriesea secundiflora* Leme | Ep |  | X | X |  |  |  |
|  | *Vriesea seideliana* W.Weber | Ep |  | X | X |  |  |  |
|  | *Vriesea serrana* E.Pereira & I.A.Penna | Ep |  | X | X |  |  |  |
|  | *Vriesea silvana* Leme | Ep |  | X | X |  |  |  |
|  | *Vriesea simplex* (Vell.) Beer | Ep |  | X | X |  |  |  |
|  | *Vriesea sparsiflora* L.B.Sm. | Ep |  | X | X |  |  |  |
|  | *Vriesea sucrei* L.B.Sm. & R.W.Read | Ep | EN | X | X |  |  |  |
|  | *Vriesea taritubensis* E.Pereira & I.A.Penna | Ep |  | X | X |  |  |  |
|  | *Vriesea teresopolitana* Leme | Ep |  | X | X |  |  |  |
|  | *Vriesea thyrsoidea* Mez | Ep |  | X | X |  |  |  |
|  | *Vriesea tijucana* E.Pereira | Ep |  | X | X |  |  |  |
|  | *Vriesea triangularis* Reitz | Ep |  | X | X |  |  |  |
|  | *Vriesea triligulata* Mez | Ep |  | X | X |  |  |  |
|  | *Vriesea unilateralis* (Baker) Mez | Ep |  | X | X |  |  |  |
|  | *Vriesea vagans* (L.B.Sm.) L.B.Sm. | Ep |  | X | X |  |  |  |
|  | *Vriesea vellozicola* Leme & J.A.Siqueira | Ep |  | X | X |  |  |  |
|  | *Vriesea vidalii* L.B.Sm. & Handro | Ep |  | X | X |  |  |  |
|  | *Vriesea vulpinoidea* L.B.Sm. | Ep |  | X | X |  |  |  |
|  | *Vriesea wawranea* Antoine | Ep | EN |  | X |  |  |  |
|  | *Vriesea weberi* E.Pereira & I.A.Penna | Ep | CR | X | X |  |  |  |
|  | *Wittrockia cyanthiformis* (Vell.) Leme | Ep |  | X | X |  |  |  |
|  | *Wittrockia gigantea* (Baker) Leme | Ep |  | X | X |  |  |  |
|  | *Wittrockia paulistana* Leme | Ep |  | X | X |  |  |  |
|  | *Wittrockia spiralipetala* Leme | Ep |  | X | X |  |  |  |
|  | *Wittrockia superba* Lindm. | Ep | EN | X | X |  |  |  |
|  | *Wittrockia tenuisepala* (Leme) Leme | Ep |  | X | X |  |  |  |
| **Cactaceae** | *Epiphyllum phyllanthus* (L.) Haw. | Ep |  |  | X | X | X | X |
|  | *Hatiora cylindrica* Britton & Rose | Ep |  | X | X |  |  |  |
|  | *Hatiora epiphylloides* (Porto & Werderm.) Buxb. | Ep |  | X | X |  |  |  |
|  | *Hatiora gaertneri* (Regel) Barthlott | Ep |  | X | X |  |  |  |
|  | *Hatiora herminiae* (Porto & Castell.) Backeb. ex Barthlott | Ep | VU | X | X |  |  |  |
|  | *Hatiora rosea* (Lagerh.) Barthlott | Ep |  | X | X |  |  |  |
|  | *Hatiora salicornioides* (Haw.) Britton & Rose | Ep |  | X | X |  |  |  |
|  | *Lepismium cruciforme* (Vell.) Miq. | Ep |  |  | X |  |  |  |
|  | *Lepismium houlletianum* (Lem.) Barthlott | Ep |  |  | X |  |  |  |
|  | *Lepismium lumbricoides* (Lem.) Barthlott | Ep |  | X | X |  |  |  |
|  | *Lepismium warmingianum* (K.Schum.) Barthlott | Ep |  |  | X |  |  |  |
|  | *Rhipsalis agudoensis* N.P.Taylor | Ep |  | X | X |  |  |  |
|  | *Rhipsalis baccifera* (J.M.Muell.) Stearn | Ep |  |  | X | X | X |  |
|  | *Rhipsalis burchellii* Britton & Rose | Ep |  | X | X |  |  |  |
|  | *Rhipsalis campos-portoana* Loefgr. | Ep |  | X | X |  |  |  |
|  | *Rhipsalis cereoides* (Backeb. & Voll) Backeb. | Ep | CR | X | X |  |  |  |
|  | *Rhipsalis cereuscula* Haw. | Ep |  | X | X |  |  |  |
|  | *Rhipsalis clavata* F.A.C.Weber | Ep |  | X | X |  |  |  |
|  | *Rhipsalis crispata* (Haw.) Pfeiffer | Ep |  | X | X |  |  |  |
|  | *Rhipsalis dissimilis* (G.Lindb.) K.Schum. | Ep |  | X | X |  |  |  |
|  | *Rhipsalis elliptica* G.Lindb. ex K.Schum. | Ep |  | X | X |  |  |  |
|  | *Rhipsalis ewaldiana* Barthlott & N.P.Taylor | Ep |  | X | X |  |  |  |
|  | *Rhipsalis floccosa* (Lagerh.) Britton & Rose | Ep |  |  | X |  | X | X |
|  | *Rhipsalis grandiflora* Haw. | Ep |  | X | X |  |  |  |
|  | *Rhipsalis hileiabaiana* (N.P.Taylor & Barthlott) N. Korotkova & Barthlott | Ep |  | X | X |  |  |  |
|  | *Rhipsalis hoelleri* Barthlott & N.P.Taylor | Ep |  | X | X |  |  |  |
|  | *Rhipsalis juengeri* Barthlott & N.P.Taylor | Ep |  | X | X |  |  |  |
|  | *Rhipsalis lindbergiana* K.Schum. | Ep |  | X | X |  |  | X |
|  | *Rhipsalis mesembryanthemoides* Haw. | Ep |  | X | X |  |  |  |
|  | *Rhipsalis neves-armondii* K.Schum. | Ep |  | X | X |  |  |  |
|  | *Rhipsalis oblonga* Loefgr. | Ep |  | X | X |  |  |  |
|  | *Rhipsalis olivifera* N.P.Taylor & Zappi | Ep |  | X | X |  |  |  |
|  | *Rhipsalis ormindoi* N.P.Taylor & Zappi | Ep |  | X | X |  |  |  |
|  | *Rhipsalis pachecoleonis* Loefgr. | Ep | EN | X | X |  |  |  |
|  | *Rhipsalis pachyptera* Pfeiff. | Ep |  | X | X |  |  |  |
|  | *Rhipsalis paradoxa* (Salm-Dyck ex Pfeiff.) Salm-Dyck | Ep | EN | X | X |  |  | X |
|  | *Rhipsalis pentaptera* A.Dietr. | Ep |  | X | X |  |  |  |
|  | *Rhipsalis pilocarpa* Loefgr. | Ep |  | X | X |  |  |  |
|  | *Rhipsalis pulchra* Loefgr. | Ep |  | X | X |  |  |  |
|  | *Rhipsalis puniceodiscus* G.Lindb. | Ep |  | X | X |  |  |  |
|  | *Rhipsalis russellii* Britton & Rose | Ep | VU | X | X |  | X | X |
|  | *Rhipsalis sulcata* F.A.C.Weber | Ep |  | X | X |  |  |  |
|  | *Rhipsalis teres* (Vell.) Steud. | Ep |  | X | X |  |  |  |
|  | *Rhipsalis trigona* Pfeiff. | Ep |  | X | X |  |  |  |
|  | *Schlumbergera kautskyi* (Horobin & McMillan) N.P.Taylor | Ep | EN | X | X |  |  |  |
|  | *Schlumbergera microsphaerica* (K.Schum.) Hoevel | Ep | VU | X | X |  |  |  |
|  | *Schlumbergera opuntioides* (Loefgr. & Dusen) D.Hunt. | Ep | VU | X | X |  |  |  |
|  | *Schlumbergera orssichiana* Barthlott & McMillan | Ep |  | X | X |  |  |  |
|  | *Schlumbergera russeliana* (Hook.) Britton & Rose | Ep |  | X | X |  |  |  |
|  | *Schlumbergera truncata* (Haw.) Moran | Ep |  | X | X |  |  |  |
| **Clusiaceae** | *Clusia criuva* Cambess. | Hep |  | X | X |  | X |  |
|  | *Clusia dardanoi* G.Mariz | Hep |  | X | X |  |  | X |
|  | *Clusia fluminensis* Planch. & Triana | Hep |  | X | X |  |  |  |
|  | *Clusia hoffmannseggiana* Schltdl. | Hep |  |  | X | X |  |  |
|  | *Clusia lanceolata* Cambess. | Hep |  | X | X |  |  |  |
|  | *Clusia panapanari* (Aubl.) Choisy | Hep |  |  | X | X |  | X |
|  | *Clusia paralicola* G.Mariz | Hep |  | X | X |  |  | X |
|  | *Clusia studartiana* C.Vieira & A.Gomes da Silva | Hep |  | X | X |  |  |  |
| **Cyclanthaceae** | *Asplundia brachypus* (Drude) Harl. | Hep |  | X | X |  |  |  |
|  | *Asplundia gardneri* (Hook.) Harling | Hep |  | X | X |  |  | X |
|  | *Asplundia maximiliani* Harling | Hep |  | X | X |  |  |  |
|  | *Asplundia polymera* (Hand.-Mzt.) Harl. | Hep |  | X | X |  |  |  |
|  | *Evodianthus funifer* (Poit.) Lindm. | Hep |  |  | X | X |  |  |
|  | *Thoracocarpus bissectus* (Vell.) Harling | Hep |  |  | X | X |  |  |
| **Dryopteridaceae** | *Elaphoglossum amorimii* F.B.Matos & Mickel | Ep |  | X | X |  |  |  |
|  | *Elaphoglossum bradeanum* Melo & Salino | Ep |  | X | X |  |  |  |
|  | *Elaphoglossum brevipetiolatum* F.B.Matos & Mickel | Ep |  | X | X |  |  |  |
|  | *Elaphoglossum burchelii* (Baker) C.Chr. | Ep |  |  | X |  |  |  |
|  | *Elaphoglossum decoratum* (Kunze) T.Moore | Ep |  |  | X | X |  |  |
|  | *Elaphoglossum didymoglossoides* C.Chr. | Ep |  | X | X |  |  |  |
|  | *Elaphoglossum dutrae* Brade | Ep |  | X | X |  |  |  |
|  | *Elaphoglossum edwalii* Rosenst. | Ep |  | X | X | X | X |  |
|  | *Elaphoglossum gardnerianum* (Kunze ex Fée) T.Moore | Ep |  | X | X |  |  |  |
|  | *Elaphoglossum gayanum* (Fée) T.Moore | Ep |  |  | X |  |  |  |
|  | *Elaphoglossum glabellum* J.Sm. | Ep |  |  | X | X |  |  |
|  | *Elaphoglossum glaziovii* (Fée) Brade | Ep |  | X | X |  |  |  |
|  | *Elaphoglossum herminieri* (Bory ex Fée) T.Moore | Ep |  |  | X |  |  |  |
|  | *Elaphoglossum horridulum* (Kaulf.) J.Sm. | Ep |  |  | X |  | X |  |
|  | *Elaphoglossum iguapense* Brade | Ep |  | X | X |  |  |  |
|  | *Elaphoglossum itatiayense* Rosenst. | Ep |  |  | X |  |  |  |
|  | *Elaphoglossum langsdorffii* (Hook. & Grev.) T.Moore | Ep |  |  | X |  | X |  |
|  | *Elaphoglossum lingua* (C.Presl) Brack. | Ep |  | X | X |  | X |  |
|  | *Elaphoglossum macahense* (Fée) Rosenst. | Ep |  | X | X |  |  |  |
|  | *Elaphoglossum muscosum* (Sw.)T.Moore | Ep |  |  | X |  |  |  |
|  | *Elaphoglossum nigrescens* (Hook.) T.Moore ex Diels | Ep |  |  | X | X |  |  |
|  | *Elaphoglossum pachydermum* (Fée) T.Moore | Ep |  | X | X |  |  |  |
|  | *Elaphoglossum paulistanum* Rosenst. | Ep |  | X | X |  |  |  |
|  | *Elaphoglossum peltatum* (Sw.) Urb. | Ep |  |  | X | X |  |  |
|  | *Elaphoglossum plumosum* (Fée) T.Moore | Ep |  |  | X | X |  |  |
|  | *Elaphoglossum pteropus* C.Chr. | Ep |  |  | X | X | X |  |
|  | *Elaphoglossum scolopendrifolium* (Raddi) J.Sm. | Ep |  | X | X |  |  |  |
|  | *Elaphoglossum squamipes* (Hook.) T.Moore | Ep |  |  | X | X |  |  |
|  | *Elaphoglossum strictum* (Raddi) T.Moore | Ep |  |  | X |  | X |  |
|  | *Elaphoglossum tectum* (Willd.) T.Moore | Ep |  |  | X |  |  |  |
|  | *Elaphoglossum vagans* (Mett.) Hieron. | Ep |  | X | X |  | X |  |
|  | *Mickelia ×atrans* R.C.Moran et al. | Hep |  | X | X |  |  |  |
|  | *Mickelia pradoi* R.C.Moran et al. | Hep |  | X | X |  |  |  |
|  | *Mickelia scandens* (Raddi) R.C.Moran et al. | Hep |  | X | X |  |  |  |
|  | *Olfersia cervina* (L.) Kunze | Ep |  |  | X | X | X |  |
|  | *Polybotrya cylindrica* Kaulf. | Hep |  | X | X |  |  |  |
|  | *Polybotrya espiritosantensis* Brade | Hep |  | X | X |  |  |  |
|  | *Polybotrya goyazensis* Brade | Hep |  | X | X | X | X |  |
|  | *Polybotrya semipinnata* Fée | Hep |  | X | X |  |  |  |
|  | *Polybotrya speciosa* Schott | Hep |  | X | X |  |  |  |
|  | *Rumohra adiantiformis* (G.Forst) Ching | Ep |  |  | X |  | X |  |
| **Gesneriaceae** | *Codonanthe carnosa* (Gardner) Hanst. | Ep |  | X | X |  |  |  |
|  | *Codonanthe cordifolia* Chautems | Ep |  | X | X |  |  |  |
|  | *Codonanthe devosiana* Lem. | Ep |  | X | X |  |  |  |
|  | *Codonanthe gibbosa* Rossini & Chautems | Ep |  | X | X |  |  |  |
|  | *Codonanthe gracilis* (Mart.) Hanst. | Ep |  | X | X |  |  |  |
|  | *Codonanthe mattos-silvae* Chautems | Ep |  | X | X |  |  |  |
|  | *Codonanthe serrulata* Chautems | Ep |  | X | X |  |  |  |
|  | *Codonanthe venosa* Chautems | Ep |  | X | X |  |  |  |
|  | *Codonanthopsis uleana* (Fritsch) Chautems & Mat.Perret | Ep |  |  | X | X |  |  |
|  | *Columnea sanguinea* (Pers.) Hanst. | Ep |  |  | X | X | X |  |
|  | *Columnea ulei* Mansf. | Ep |  | X | X |  |  |  |
|  | *Drymonia coccinea* (Aubl.) Wiehler | Ep |  |  | X | X |  |  |
|  | *Drymonia serrulata* (Poepp.) Wiehler | Ep |  |  | X | X | X | X |
|  | *Nematanthus albus* Chautems | Ep |  | X | X |  |  |  |
|  | *Nematanthus australis* Chautems | Ep |  | X | X |  |  |  |
|  | *Nematanthus bradei* (Handro) Chautems | Ep |  | X | X |  |  |  |
|  | *Nematanthus brasiliensis* (Vell.) Chautems | Ep |  | X | X |  |  |  |
|  | *Nematanthus corticola* Schrad. | Ep |  | X | X |  |  |  |
|  | *Nematanthus crassifolius* (Schott) Wiehler | Ep |  | X | X |  |  |  |
|  | *Nematanthus fissus* (Vell.) L.E.Skog | Ep |  | X | X |  |  |  |
|  | *Nematanthus fluminensis* (Vell.) Fritsch | Ep |  | X | X |  |  |  |
|  | *Nematanthus fornix* (Vell.) Chautems | Ep |  | X | X |  |  |  |
|  | *Nematanthus fritschii* Hoehne | Ep |  | X | X |  |  |  |
|  | *Nematanthus gregarius* D.L.Denham | Ep |  | X | X |  |  |  |
|  | *Nematanthus hirtellus* (Schott) Wiehler | Ep |  | X | X |  |  |  |
|  | *Nematanthus jolyanus* (Handro) Chautems | Ep |  | X | X |  |  |  |
|  | *Nematanthus kautskyi* Chautems & Rossini | Ep |  | X | X |  |  |  |
|  | *Nematanthus kuhlmannii* (Handro) Chautems | Ep |  | X | X |  |  |  |
|  | *Nematanthus lanceolatus* (Poir.) Chautems | Ep |  | X | X |  | X |  |
|  | *Nematanthus maculatus* (Fritsch) Wiehler | Ep |  | X | X |  |  |  |
|  | *Nematanthus mattosianus* (Handro) H.E.Moore | Ep |  | X | X |  |  |  |
|  | *Nematanthus mirabilis* (Handro) Chautems | Ep |  | X | X |  |  |  |
|  | *Nematanthus monanthos* (Vell.) Chautems | Ep |  | X | X |  |  |  |
|  | *Nematanthus punctatus* Chautems | Ep |  | X | X |  |  |  |
|  | *Nematanthus pycnophyllus* Chautems et al. | Ep |  | X | X |  |  |  |
|  | *Nematanthus sericeus* (Hanst.) Chautems | Ep |  | X | X |  | X |  |
|  | *Nematanthus serpens* (Vell.) Chautems | Ep |  | X | X |  |  |  |
|  | *Nematanthus striatus* (Handro) Chautems | Ep |  | X | X |  |  |  |
|  | *Nematanthus strigillosus* (Mart.) H.E.Moore | Ep |  | X | X |  | X |  |
|  | *Nematanthus teixeiranus* (Handro) Chautems | Ep |  | X | X |  |  |  |
|  | *Nematanthus tessmannii* (Hoehne) Chautems | Ep |  | X | X |  |  |  |
|  | *Nematanthus villosus* (Hanst.) Wiehler | Ep |  | X | X |  |  |  |
|  | *Nematanthus wettsteinii* (Fritsch) H.E.Moore | Ep |  | X | X |  |  |  |
|  | *Nematanthus wiehleri* Chautems & M.Peixoto | Ep |  | X | X |  |  |  |
|  | *Sinningia cooperi* (Paxton) Wiehler | Ep |  | X | X |  |  |  |
|  | *Sinningia douglasii* (Lindl.) Chautems | Ep |  |  | X |  |  |  |
|  | *Sinningia magnifica* (Otto & A.Dietr.) Wiehler | Ep |  | X | X |  | X |  |
| **Griseliniaceae** | *Griselinia ruscifolia* (Clos) Taub. | Hep |  |  | X |  |  |  |
| **Hymenophyllaceae** | *Didymoglossum angustifrons* Fée | Ep |  |  | X | X |  |  |
|  | *Didymoglossum hymenoides* (Hedw.) Copel. | Ep |  |  | X | X | X |  |
|  | *Didymoglossum kapplerianum* (J.W.Sturm) Ebihara & Dubuisson | Ep |  |  | X | X |  |  |
|  | *Didymoglossum krausii* (Hook. & Grev.) C.Presl | Ep |  |  | X | X |  |  |
|  | *Didymoglossum ovale* E.Fourn. | Ep |  |  | X | X |  |  |
|  | *Didymoglossum punctatum* (Poir.) Desv. | Ep |  |  | X | X |  |  |
|  | *Didymoglossum pusillum* (Sw.) Desv. | Ep |  |  | X |  |  |  |
|  | *Didymoglossum reptans* (Sw.) C.Presl | Ep |  |  | X |  |  |  |
|  | *Hymenophyllum asplenioides* (Sw.) Sw. | Ep |  |  | X | X |  |  |
|  | *Hymenophyllum caudiculatum* Mart. | Ep |  |  | X |  |  |  |
|  | *Hymenophyllum crispum* Kunth | Ep |  |  | X |  |  |  |
|  | *Hymenophyllum elegans* Spreng. | Ep |  |  | X | X |  |  |
|  | *Hymenophyllum filmenofilicum* Christenh. & Schwartsb. | Ep |  | X | X |  |  |  |
|  | *Hymenophyllum fragile* (Hedw.) C.V.Morton | Ep |  |  | X |  |  |  |
|  | *Hymenophyllum fucoides* (Sw.) Sw. | Ep |  |  | X | X |  |  |
|  | *Hymenophyllum glaziovii* Baker | Ep |  | X | X |  |  |  |
|  | *Hymenophyllum magellanicum* Willd. ex Kunze | Ep |  |  | X |  |  |  |
|  | *Hymenophyllum microcarpum* Desv. | Ep |  |  | X | X |  |  |
|  | *Hymenophyllum peltatum* (Poir.) Desv. | Ep |  |  | X | X |  |  |
|  | *Hymenophyllum plumosum* Kaulf. | Ep |  |  | X |  |  |  |
|  | *Hymenophyllum polyanthos* (Sw.) Sw. | Ep |  |  | X | X | X |  |
|  | *Hymenophyllum pulchellum* Schltdl. & Cham. | Ep |  |  | X |  |  |  |
|  | *Hymenophyllum rufum* Fée | Ep |  | X | X |  |  |  |
|  | *Hymenophyllum sampaioanum* Brade & Rosenst. | Ep | CR | X | X |  |  |  |
|  | *Hymenophyllum silveirae* Christ | Ep | CR | X | X |  |  |  |
|  | *Hymenophyllum undulatum* (Sw.) Sw. | Ep |  |  | X | X |  |  |
|  | *Hymenophyllum vestitum* (K.Presl) v.d.Bosch | Ep |  | X | X |  |  |  |
|  | *Hymenophyllum myriocarpum* Hook. | Ep |  |  | X | X |  |  |
|  | *Polyphlebium angustatum* (Carmich.) Ebihara & Dubuisson | Ep |  | X | X |  |  |  |
|  | *Polyphlebium diaphanum* (Kunth) Ebihara & Dubuisson | Ep |  | X | X | X |  |  |
|  | *Polyphlebium hymenophylloides* (Bosch) Ebihara & Dubuisson | Ep |  |  | X |  |  |  |
|  | *Polyphlebium pyxidiferum* (L.) Ebihara & Dubuisson | Ep |  |  | X |  |  |  |
|  | *Trichomanes accedens* C.Presl | Ep |  |  | X | X |  |  |
|  | *Trichomanes anadromum* Rosenst. | Ep |  |  | X |  | X |  |
|  | *Trichomanes arbuscula* Desv. | Ep |  |  | X | X |  |  |
|  | *Trichomanes lucens* Sw. | Ep |  |  | X |  |  |  |
|  | *Trichomanes pedicellatum* Desv. | Ep |  |  | X | X | X |  |
|  | *Trichomanes pilosum* Raddi | Ep |  |  | X |  | X |  |
|  | *Trichomanes polypodioides* L. | Ep |  |  | X |  | X |  |
|  | *Vandenboschia radicans* (Sw.) Copel. | Ep |  |  | X | X |  |  |
| **Lentibulariaceae** | *Utricularia jamesoniana* Oliv. | Ep |  |  | X | X |  |  |
|  | *Utricularia nelumbifolia* Gardner | Ep |  | X | X |  |  |  |
|  | *Utricularia reniformis* A.St.-Hil. | Ep |  | X | X |  |  |  |
|  | *Utricularia triloba* Benj. | Ep |  |  | X | X | X | X |
| **Lomariopsidaceae** | *Lomariopsis marginata* (Schrad.) Kuhn | Hep |  | X | X |  |  |  |
|  | *Nephrolepis biserrata* (Sw.) Schott | Ep |  |  | X | X | X | X |
|  | *Nephrolepis pendula* (Raddi) J.Sm. | Ep |  |  | X | X | X |  |
|  | *Nephrolepis rivularis* (Vahl) Mett. ex Krug | Ep |  |  | X | X | X |  |
| **Lycopodiaceae** | *Phlegmariurus acerosus* (Sw.) B.Øllg. | Ep |  |  | X |  |  |  |
|  | *Phlegmariurus aqualupianus* (Spring) B.Øllg. | Ep | EN |  | X |  |  |  |
|  | *Phlegmariurus biformis* (Hook.) B.Øllg. | Ep |  | X | X |  |  |  |
|  | *Phlegmariurus comans* (Nessel) B.Øllg. | Ep |  | X | X |  |  |  |
|  | *Phlegmariurus flexibilis* (Fée) B.Øllg. | Ep |  | X | X | X |  |  |
|  | *Phlegmariurus fontinaloides* (Spring) B.Øllg. | Ep |  | X | X |  |  |  |
|  | *Phlegmariurus heterocarpon* (Fée) B.Øllg. | Ep |  |  | X |  |  |  |
|  | *Phlegmariurus hexastichus* (B.Øllg. & P.G.Windisch) B.Øllg. | Ep |  | X | X |  |  |  |
|  | *Phlegmariurus loefgrenianus* (Silveira) B.Øllg. | Ep |  | X | X |  |  |  |
|  | *Phlegmariurus mandiocanus* (Raddi) B.Øllg. | Ep |  |  | X |  | X |  |
|  | *Phlegmariurus martii* (Wawra) B.Øllg. | Ep | EN | X | X |  | X |  |
|  | *Phlegmariurus mollicomus* (Spring) B.Øllg. | Ep | EN |  | X | X |  |  |
|  | *Phlegmariurus nudus* (Nessel) B.Øllg. | Ep | EN | X | X |  |  |  |
|  | *Phlegmariurus quadrifariatus* (Bory) B.Øllg. | Ep |  | X | X |  |  |  |
|  | *Phlegmariurus silveirae* (Nessel) B.Øllg. | Ep |  | X | X |  |  |  |
|  | *Phlegmariurus taxifolius* (Sw.) Á.Löve & D.Löve | Ep | EN |  | X | X |  |  |
| **Malvaceae** | *Spirotheca rivieri* (Decne.) Ulbr. | Hep |  | X | X |  |  |  |
| **Marcgraviaceae** | *Marcgravia comosa* C.Presl | Hep |  | X | X |  |  |  |
|  | *Marcgravia coriacea* Vahl | Hep |  |  | X | X | X | X |
|  | *Marcgravia picta* Willd. | Hep |  | X | X | X |  |  |
|  | *Marcgravia polyantha* Delpino | Hep |  | X | X |  | X |  |
|  | *Marcgraviastrum cuneifolium* (Gardner) Bedell | Hep |  | X | X |  |  |  |
|  | *Marcgraviastrum delpinianum* (Wittm.) Gir.-Cañas | Hep |  | X | X |  |  |  |
|  | *Schwartzia adamantium* (Cambess.) Bedell ex Gir.-Cañas | Hep |  | X | X |  | X |  |
|  | *Schwartzia brasiliensis* (Choisy) Bedell ex Gir.-Cañas | Hep |  | X | X |  | X |  |
|  | *Schwartzia geniculatiflora* Gir.-Cañas & Fiaschi | Hep |  | X | X |  |  |  |
|  | *Schwartzia jucuensis* Gir.-Cañas | Hep |  | X | X |  |  |  |
|  | *Souroubea guianensis* Aubl. | Hep |  |  | X | X |  |  |
|  | *Souroubea platyadenia* (Gilg) de Roon | Hep |  | X | X |  |  |  |
| **Melastomataceae** | *Bertolonia alternifolia* Baumgratz et al. | Ep |  | X | X |  |  |  |
|  | *Bertolonia bullata* Baumgratz et al. | Ep |  | X | X |  |  |  |
|  | *Pleiochiton blepharodes* (DC.) Reginato et al. | Ep |  | X | X |  |  |  |
|  | *Pleiochiton crassifolium* A.Gray | Ep |  | X | X |  |  |  |
|  | *Pleiochiton ebracteatum* Triana | Ep |  | X | X |  |  |  |
|  | *Pleiochiton glaziovianum* Cogn. | Ep |  | X | X |  |  |  |
|  | *Pleiochiton longipetiolatum* Brade | Ep |  | X | X |  |  |  |
|  | *Pleiochiton magdalenesis* Brade | Ep |  | X | X |  |  |  |
|  | *Pleiochiton micranthum* Cogn. | Ep |  | X | X |  |  |  |
|  | *Pleiochiton parasiticum* (Triana) Reginato et al. | Ep |  | X | X |  |  |  |
|  | *Pleiochiton parvifolium* Cogn. | Ep |  | X | X |  |  |  |
|  | *Pleiochiton roseum* Cogn. | Ep |  | X | X |  |  |  |
|  | *Pleiochiton setulosum* Cogn. | Ep |  | X | X |  |  |  |
| **Moraceae** | *Ficus arpazusa* Casar. | Hep |  |  | X | X | X | X |
|  | *Ficus bahiensis* C.C.Berg & Carauta | Hep |  | X | X |  | X | X |
|  | *Ficus broadwayi* Urb. | Hep |  |  | X | X | X | X |
|  | *Ficus calyptroceras* (Miq.) Miq. | Hep |  |  | X |  | X | X |
|  | *Ficus castellviana* Dugand | Hep |  |  | X | X |  | X |
|  | *Ficus catappifolia* Kunth & C.D.Bouché | Hep |  |  | X | X | X | X |
|  | *Ficus cestrifolia* Schott ex Spreng. | Hep |  | X | X |  |  |  |
|  | *Ficus christianii* Carauta | Hep |  | X | X | X |  |  |
|  | *Ficus citrifolia* Mill. | Hep |  |  | X | X | X | X |
|  | *Ficus clusiifolia* Schott | Hep |  | X | X |  |  |  |
|  | *Ficus cyclophylla* (Miq.) Miq. | Hep | VU | X | X |  |  |  |
|  | *Ficus duartei* C.C.Berg & Carauta | Hep |  |  | X |  |  |  |
|  | *Ficus enormis* Mart. ex Miq. | Hep |  | X | X |  | X | X |
|  | *Ficus eximia* Schott | Hep |  | X | X | X | X |  |
|  | *Ficus gomelleira* Kunth & C.D.Bouché | Hep |  |  | X | X | X | X |
|  | *Ficus guaranitica* Chodat | Hep |  |  | X |  | X |  |
|  | *Ficus guianensis* Desv. | Hep |  |  | X | X | X | X |
|  | *Ficus hirsuta* Schott | Hep |  | X | X |  |  | X |
|  | *Ficus holosericea* Schott | Hep |  | X | X | X |  |  |
|  | *Ficus lagoensis* C.C.Berg & Carauta | Hep |  | X | X |  |  |  |
|  | *Ficus laureola* Warb. ex C.C.Berg & Carauta | Hep |  | X | X |  |  |  |
|  | *Ficus longifolia* Schott | Hep |  | X | X |  |  |  |
|  | *Ficus luschnathiana* (Miq.) Miq. | Hep |  |  | X |  | X |  |
|  | *Ficus mariae* C.C.Berg et al. | Hep |  |  | X |  |  |  |
|  | *Ficus mathewsii* (Miq.) Miq. | Hep |  |  | X | X | X | X |
|  | *Ficus mexiae* Standl. | Hep |  | X | X |  | X | X |
|  | *Ficus monckii* Hassl. | Hep |  |  | X |  |  |  |
|  | *Ficus nymphaeifolia* Mill. | Hep |  |  | X | X | X | X |
|  | *Ficus obtusifolia* Kunth | Hep |  |  | X | X | X | X |
|  | *Ficus organensis* (Miq.) Miq. | Hep |  | X | X |  |  |  |
|  | *Ficus pakkensis* Standl. | Hep |  |  | X | X |  |  |
|  | *Ficus pertusa* L.f. | Hep |  |  | X | X | X |  |
|  | *Ficus salzmanniana* (Miq.) Miq. | Hep |  | X | X |  |  | X |
|  | *Ficus tomentella* (Miq.) Miq. | Hep |  |  | X | X | X |  |
|  | *Ficus trigona* L.f. | Hep |  |  | X | X | X | X |
|  | *Ficus trigonata* L. | Hep |  | X | X | X | X |  |
| **Oleandraceae** | *Oleandra articulata* (Sw.) C.Presl | Ep |  |  | X | X | X |  |
| **Onagraceae** | *Fuchsia regia* (Vell.) Munz | Hep |  | X | X |  | X |  |
| **Ophioglossaceae** | *Ophioglossum palmatum* L. | Ep |  |  | X | X |  |  |
| **Orchidaceae** | *Acianthera acuminatipetala* (A.Samp.) Luer | Ep |  | X | X |  |  |  |
|  | *Acianthera adirii* (Brade) Pridgeon & M.W.Chase | Ep | CR | X | X |  |  |  |
|  | *Acianthera alborosea* (Kraenzl.) Luer | Ep |  | X | X |  |  |  |
|  | *Acianthera antennata* (Garay) Pridgeon & M.W.Chase | Ep |  | X | X |  |  |  |
|  | *Acianthera aphthosa* (Lindl.) Pridgeon & M.W.Chase | Ep |  | X | X |  |  |  |
|  | *Acianthera asaroides* (Kraenzl.) Pridgeon & M.W.Chase | Ep |  | X | X |  |  |  |
|  | *Acianthera atroglossa* (Loefgr.) F.Barros & L. Guimarães | Ep |  | X | X |  |  |  |
|  | *Acianthera aurantiaca* (Barb.Rodr.) Campacci | Ep |  | X | X |  |  |  |
|  | *Acianthera auriculata* (Lindl.) Pridgeon & M.W.Chase | Ep |  | X | X |  |  |  |
|  | *Acianthera bidentula* (Barb.Rodr.) Pridgeon & M.W.Chase | Ep |  | X | X |  |  |  |
|  | *Acianthera binotii* (Regel) Pridgeon & M.W.Chase | Ep |  | X | X |  |  |  |
|  | *Acianthera brachyloba* (Hoehne) Pridgeon & M.W.Chase | Ep |  | X | X |  |  |  |
|  | *Acianthera bragae* (Ruschi) F.Barros | Ep |  | X | X |  |  |  |
|  | *Acianthera caldensis* (Hoehne & Schltr.) F.Barros | Ep |  | X | X |  |  |  |
|  | *Acianthera capanemae* (Barb.Rodr.) Pridgeon & M.W.Chase | Ep |  | X | X |  |  |  |
|  | *Acianthera capillaris* (Lindl.) Pridgeon & M.W.Chase | Ep |  | X | X |  |  |  |
|  | *Acianthera crinita* (Barb.Rodr.) Pridgeon & M.W.Chase | Ep |  |  | X |  |  |  |
|  | *Acianthera cristata* (Barb.Rodr.) Luer | Ep |  | X | X |  |  |  |
|  | *Acianthera cryptantha* (Barb.Rodr.) Pridgeon & M.W.Chase | Ep |  | X | X |  |  |  |
|  | *Acianthera cryptophoranthoides* (Loefgr.) Pridgeon & M.W.Chase | Ep |  | X | X |  | X |  |
|  | *Acianthera duartei* (Hoehne) Pridgeon & M.W.Chase | Ep |  | X | X |  |  |  |
|  | *Acianthera dutrae* (Pabst) C.N.Conç. & Waechter | Ep |  | X | X |  |  |  |
|  | *Acianthera exarticulata* (Barb.Rodr.) Pridgeon & M.W.Chase | Ep |  | X | X |  |  |  |
|  | *Acianthera exdrasii* (Luer & Toscano) Luer | Ep |  | X | X |  |  |  |
|  | *Acianthera fenestrara* (Barb.Rodr.) Pridgeon & M.W.Chase | Ep |  | X | X |  |  |  |
|  | *Acianthera freyi* (Luer) F.Barros & V.T.Rodrigues | Ep |  | X | X |  |  |  |
|  | *Acianthera glanduligera* (Lindl.) Luer | Ep |  | X | X |  |  |  |
|  | *Acianthera glumacea* (Lindl.) Pridgeon & M.W.Chase | Ep |  | X | X |  |  |  |
|  | *Acianthera gouveiae* (A. Samp.) F. Barros & L.Guimarães | Ep |  | X | X |  |  |  |
|  | *Acianthera gracilisepala* (Brade) Luer | Ep |  | X | X |  |  |  |
|  | *Acianthera guimaraensii* (Brade) F.Barros | Ep |  | X | X |  |  |  |
|  | *Acianthera heliconiscapa* (Hoehne) F.Barros | Ep |  | X | X |  |  |  |
|  | *Acianthera heringeri* (Hoehne) F.Barros | Ep | CR | X | X |  |  |  |
|  | *Acianthera hoffmannseggiana* (Rchb.f.) Luer | Ep |  | X | X |  |  |  |
|  | *Acianthera hygrophila* (Berb.Rodr.) Pridgeon &M.W.Chase | Ep |  | X | X |  |  |  |
|  | *Acianthera hystrix* (Kraenzl.) F.Barros | Ep |  |  | X |  |  |  |
|  | *Acianthera jordanensis* (Brade) F.Barros | Ep |  | X | X |  |  |  |
|  | *Acianthera karlii* (Pabst) C.N.Conç. & Waechter | Ep |  | X | X |  |  |  |
|  | *Acianthera klotzschiana* (Rchb.f.) Pridgeon & M.W.Chase | Ep |  | X | X |  |  |  |
|  | *Acianthera langeana* (Kraenzl.) Pridgeon & M.W.Chase | Ep | EN | X | X |  |  |  |
|  | *Acianthera leptotifolia* (Barb.Rodr.) Pridgeon & M.W.Chase | Ep |  | X | X |  |  |  |
|  | *Acianthera luteola* (Lindl.) Pridgeon & M.W.Chase | Ep |  |  | X |  |  |  |
|  | *Acianthera macropoda* (Barb.Rodr.) Pridgeon & M.W.Chase | Ep |  | X | X |  |  |  |
|  | *Acianthera macuconensis* (Barb.Rodr.) F.Barros | Ep |  |  | X |  |  |  |
|  | *Acianthera magalhanesii* (Pabst) F.Barros | Ep |  | X | X |  |  |  |
|  | *Acianthera malachantha* (Rchb.f.) Pridgeon & M.W.Chase | Ep |  | X | X |  |  |  |
|  | *Acianthera marumbyana* (Garay) Luer | Ep |  | X | X |  |  |  |
|  | *Acianthera melachyla* (Barb.Rodr.) Luer | Ep |  | X | X |  |  |  |
|  | *Acianthera micrantha* (Barb.Rodr.) Pridgeon & M.W.Chase | Ep |  | X | X |  |  |  |
|  | *Acianthera muscicola* (Barb.Rodr.) Pridgeon & M.W.Chase | Ep |  | X | X |  |  |  |
|  | *Acianthera muscosa* (Barb.Rodr.) Pridgeon & M.W.Chase | Ep |  | X | X |  |  |  |
|  | *Acianthera myrticola* (Barb. Rodr.) F. Barros & L.Guimarães | Ep |  | X | X |  |  |  |
|  | *Acianthera nemorosa* (Barb.Rodr.) F.Barros | Ep |  | X | X |  |  |  |
|  | *Acianthera octophrys* (Rchb.f.) Pridgeon & M.W.Chase | Ep |  | X | X |  |  |  |
|  | *Acianthera oligantha* (Barb.Rodr.) F.Barros | Ep |  | X | X |  |  |  |
|  | *Acianthera panduripetala* (Barb.Rodr.) Pridgeon & M.W.Chase | Ep |  | X | X |  |  |  |
|  | *Acianthera papillosa* (Lindl.) Pridgeon & M.W.Chase | Ep | VU | X | X |  |  |  |
|  | *Acianthera pardipes* (Rchb.f.) Pridgeon & M.W.Chase | Ep |  | X | X |  |  |  |
|  | *Acianthera parva* (Rolfe) F.Barros & L.Guimarães | Ep |  | X | X |  | X |  |
|  | *Acianthera pectinata* (Lindl.) Pridgeon & M.W.Chase | Ep |  | X | X |  |  |  |
|  | *Acianthera perdusenii* (Hoehne) F.Barros & L.Guimarães | Ep |  | X | X |  |  |  |
|  | *Acianthera pubescens* (Lindl.) Pridgeon & M.W.Chase | Ep |  |  | X |  | X | X |
|  | *Acianthera punctata* (Barb.Rodr.) F.Barros | Ep |  | X | X |  |  |  |
|  | *Acianthera purpureoviolacea* (Cogn.) F.Barros | Ep |  | X | X |  |  |  |
|  | *Acianthera ramosa* (Barb.Rodr.) F.Barros | Ep |  | X | X |  | X |  |
|  | *Acianthera recurva* (Lindl.) Pridgeon & M.W.Chase | Ep |  |  | X |  |  |  |
|  | *Acianthera rostellata* (Barb.Rodr.) Luer | Ep |  | X | X |  |  |  |
|  | *Acianthera saundersiana* (Rchb.f.) Pridgeon & M.W.Chase | Ep |  |  | X |  | X | X |
|  | *Acianthera saurocephala* (Lodd.) Pridgeon & M.W.Chase | Ep |  | X | X |  |  |  |
|  | *Acianthera serpentula* (Barb.Rodr.) F.Barros | Ep |  | X | X |  |  |  |
|  | *Acianthera sonderana* (Rchb.f.) Pridgeon & M.W.Chase | Ep |  | X | X |  |  |  |
|  | *Acianthera spilantha* (Barb.Rodr.) Luer | Ep |  | X | X |  |  |  |
|  | *Acianthera strupifolia* (Lindl.) Pridgeon & M.W.Chase | Ep |  | X | X |  |  |  |
|  | *Acianthera subrotundifolia* (Cogn.) F.Barros & V.T.Rodrigues | Ep |  | X | X |  |  |  |
|  | *Acianthera sulcata* (Porsch) F. Barros & V.T.Rodrigues | Ep |  | X | X |  |  |  |
|  | *Acianthera sulphurea* (Barb. Rodr.) F. Barros & V.T.Rodrigues | Ep |  | X | X |  |  |  |
|  | *Acianthera translucida* (Barb.Rodr.) Luer | Ep |  | X | X |  |  |  |
|  | *Acianthera tricarinata* (Poepp. & Endl.) Pridgeon & M.W.Chase | Ep |  | X | X |  |  |  |
|  | *Acianthera tristis* (Barb.Rodr.) Pridgeon & M.W.Chase | Ep |  | X | X |  |  |  |
|  | *Acianthera variegata* (Barb.Rodr.) Campacci | Ep |  | X | X |  |  |  |
|  | *Acianthera violaceomaculata* (Hoehne) Pridgeon & M.W.Chase | Ep |  | X | X |  |  |  |
|  | *Acianthera wageneriana* (Klotzsch) Pridgeon & M.W.Chase | Ep |  | X | X |  |  |  |
|  | *Acianthera wawraeana* (Barb. Rodr.) F.Barros & V.T.Rodrigues | Ep |  | X | X |  |  |  |
|  | *Acianthera wels-windischiae* (Pabst) Pridgeon & M.W.Chase | Ep |  | X | X |  |  |  |
|  | *Alatiglossum barbatum* (Lindl.) Baptista | Ep |  | X | X |  |  |  |
|  | *Alatiglossum ciliatum* (Lindl.) Baptista | Ep |  | X | X |  |  |  |
|  | *Alatiglossum cognauxianum* (Schltr.) Baptista | Ep |  | X | X |  |  |  |
|  | *Alatiglossum cogniauxianum* (Schltr.) Baptista | Ep |  | X | X |  |  |  |
|  | *Alatiglossum croesus* (Rchb.f.) Baptista | Ep | CR | X | X |  |  |  |
|  | *Alatiglossum longipes* (Lindl.) Baptista | Ep |  |  | X |  |  |  |
|  | *Alatiglossum macropetalum* (Lindl.) Baptista | Ep |  |  | X |  | X |  |
|  | *Alatiglossum micropogon* (Rchb.f.) Baptista | Ep |  | X | X |  |  |  |
|  | *Alatiglossum uniflorum* (Booth.) Baptista | Ep |  | X | X |  |  |  |
|  | *Anathallis adenochila* (Loefgr.) F.Barros | Ep |  | X | X |  |  |  |
|  | *Anathallis bleyensis* (Pabst) F.Barros | Ep |  | X | X |  |  |  |
|  | *Anathallis brevipes* (H.Focke) Pridgeon & M.W.Chase | Ep |  |  | X |  | X |  |
|  | *Anathallis bocainensis* (Porto & Brade) F.Barros & Barberena | Ep |  | X | X |  |  |  |
|  | *Anathallis caroli* (Schltr.) F.Barros & Barberena | Ep |  | X | X |  |  |  |
|  | *Anathallis carvalhoi* (Luer & Toscano) Luer | Ep |  | X | X |  |  |  |
|  | *Anathallis colnagoi* (Rchb.f.) Baptista | Ep | CR | X | X |  |  |  |
|  | *Anathallis corticicola* (Schltr. ex Hoehne) Pridgeon & M.W.Chase | Ep |  | X | X |  |  | X |
|  | *Anathallis crebrifolia* (Barb.Rodr.) Luer | Ep |  | X | X |  |  |  |
|  | *Anathallis dryadum* (Schltr.) F.Barros | Ep |  | X | X |  |  |  |
|  | *Anathallis ferdinandiana* (Barb.Rodr.) F.Barros | Ep |  | X | X |  |  |  |
|  | *Anathallis fernandiana* (Hoehne) F.Barros | Ep |  | X | X |  |  |  |
|  | *Anathallis flammea* (Barb.Rodr.) F.Barros | Ep |  | X | X |  |  |  |
|  | *Anathallis gehrtii* (Hoehne & Schltr.) F.Barros | Ep | VU | X | X |  |  |  |
|  | *Anathallis gerthatschbachii* (Hoehne) Pridgeon & M.W.Chase | Ep |  | X | X |  |  |  |
|  | *Anathallis githaginea* (Pabst & Garay) Pridgeon & M.W.Chase | Ep |  | X | X |  |  |  |
|  | *Anathallis globifera* (Pabst) F. Barros & Barberena | Ep |  | X | X |  |  |  |
|  | *Anathallis graveolens* (Pabst) F.Barros | Ep |  | X | X |  |  |  |
|  | *Anathallis guarujaensis* (Hoehne) F.Barros | Ep |  | X | X |  |  |  |
|  | *Anathallis helmutii* (Hoehne) F.Barros | Ep |  | X | X |  |  |  |
|  | *Anathallis imbricata* (Barb.Rodr.) F.Barros & F.Pinheiro | Ep |  | X | X |  |  |  |
|  | *Anathallis jordanensis* (Hoehne) F.Barros | Ep |  | X | X |  |  |  |
|  | *Anathallis kautskyi* (Pabst) Pridgeon & M.W.Chase | Ep |  | X | X |  |  |  |
|  | *Anathallis kleinii* (Pabst) Luer | Ep |  | X | X |  |  |  |
|  | *Anathallis laciniata* (Barb. Rodr.) F.Barros & Barberena | Ep |  | X | X |  |  |  |
|  | *Anathallis lichenophila* (Porto & Brade) Luer | Ep |  | X | X |  |  |  |
|  | *Anathallis linearifolia* (Cogn.) Pridgeon & M.W.Chase | Ep |  | X | X |  |  |  |
|  | *Anathallis liparanges* (Rchb.f.) Luer | Ep |  | X | X |  | X |  |
|  | *Anathallis malmeana* (Dutra ex Pabst) Pridgeon & M.W.Chase | Ep |  | X | X |  |  |  |
|  | *Anathallis marginata* (Barb. Rodr.) F.Barros & Barberena | Ep |  | X | X |  |  |  |
|  | *Anathallis microblephara* (Schltr.) Pridgeon & M.W.Chase | Ep |  | X | X |  |  |  |
|  | *Anathallis microgemma* (Schltr. ex Hoehne) Pridgeon & M.W.Chase | Ep |  | X | X |  |  |  |
|  | *Anathallis microphyta* (Barb.Rodr.) C.O.Azevedo & Van den Berg | Ep |  |  | X |  |  | X |
|  | *Anathallis montipelladensis* (Hoehne) F.Barros | Ep |  | X | X |  | X | X |
|  | *Anathallis muscoidea* (Lindl.) F.Barros & Barberena | Ep |  | X | X |  |  |  |
|  | *Anathallis nectarifera* Barb.Rodr. | Ep |  | X | X |  |  |  |
|  | *Anathallis obovata* (Lindl.) Pridgeon & M.W.Chase | Ep |  |  | X |  |  |  |
|  | *Anathallis pabstii* (Garay) Pridgeon & M.W.Chase | Ep | EN | X | X |  |  |  |
|  | *Anathallis paranaensis* (Schltr.) Pridgeon & M.W.Chase | Ep |  | X | X |  |  | X |
|  | *Anathallis paranapiacabensis* (Hoehne) F.Barros | Ep |  | X | X |  |  |  |
|  | *Anathallis peroupavae* (Hoehne & Brade) F.Barros | Ep |  | X | X |  |  |  |
|  | *Anathallis petersiana* (Schltr.) Pridgeon & M.W.Chase | Ep |  | X | X |  |  |  |
|  | *Anathallis petropolitana* (Hoehne) F.Barros & Barberena | Ep |  | X | X |  |  |  |
|  | *Anathallis piratiningana* (Hoehne) F.Barros | Ep |  | X | X |  |  |  |
|  | *Anathallis pubipetala* (Hoehne) Pridgeon & M.W.Chase | Ep |  | X | X |  |  |  |
|  | *Anathallis pusilla* (Barb.Rodr.) F.Barros | Ep |  | X | X |  |  |  |
|  | *Anathallis puttemansii* (Hoehne) F.Barros | Ep |  | X | X |  |  |  |
|  | *Anathallis radialis* (Porto & Brade) Pridgeon & M.W.Chase | Ep |  | X | X |  |  |  |
|  | *Anathallis recurvipetala* (Barb. Rodr.) F.Barros & Barberena | Ep |  | X | X |  |  |  |
|  | *Anathallis reedii* (Luer) F.Barros | Ep |  | X | X |  |  |  |
|  | *Anathallis rubens* (Lindl.) Pridgeon & M.W.Chase | Ep |  |  | X |  | X | X |
|  | *Anathallis rubrolimbata* (Hoehne) F. Barros & Barberena | Ep |  | X | X |  |  |  |
|  | *Anathallis rudolfii* (Pabst) Pridgeon &M.W.Chase | Ep |  | X | X |  |  |  |
|  | *Anathallis sclerophylla* (Lindl.) Pridgeon & M.W.Chase | Ep |  | X | X |  | X | X |
|  | *Anathallis seidelii* Luer et al. | Ep |  | X | X |  |  |  |
|  | *Anathallis simpliciglossa* (Loefgr.) Pridgeon & M.W.Chase | Ep |  | X | X |  |  |  |
|  | *Anathallis sororcula* (Schltr.) Luer | Ep |  | X | X |  |  |  |
|  | *Anathallis spannageliana* (Hoehne) Pridgeon & M.W.Chase | Ep |  | X | X |  |  |  |
|  | *Anathallis stictophylla* (Schltr.) Pridgeon & M.W.Chase | Ep |  | X | X |  |  |  |
|  | *Anathallis subnulla* (Luer & Toscano) F.Barros | Ep |  | X | X |  |  |  |
|  | *Anathallis tigridens* (Loefgr.) F.Barros & Barberena | Ep | VU | X | X |  |  |  |
|  | *Anathallis trullilabia* (Pabst) F.Barros | Ep |  | X | X |  |  |  |
|  | *Anathallis vitorinoi* (Luer & Toscano) F.Barros & Barberena | Ep |  | X | X |  |  |  |
|  | *Anathallis welteri* (Pabst) F.Barros | Ep |  | X | X |  |  |  |
|  | *Anathallis ypirangae* (Kraenzl.) Pridgeon & M.W.Chase | Ep |  | X | X |  |  |  |
|  | *Aspasia lunata* Lindl. | Ep |  | X | X |  |  |  |
|  | *Aspasia silvana* F.Barros | Ep |  | X | X |  |  |  |
|  | *Baptistonia albinoi* (Schltr.) Chiron & V.P.Castro | Ep |  | X | X |  |  |  |
|  | *Baptistonia brieniana* (Rchb.f.) V.P.Castro & Chiron | Ep |  |  | X |  |  |  |
|  | *Baptistonia colorata* (Koern. & J.G.Winman) Chiron | Ep |  | X | X |  |  |  |
|  | *Baptistonia cornigera* (Lindl.) Chiron & V.P.Castro | Ep |  |  | X |  |  |  |
|  | *Baptistonia cruciata* (Rchb.f.) Chiron & V.P.Castro | Ep |  | X | X |  |  |  |
|  | *Baptistonia damacenoi* Chiron & V.P.Castro | Ep |  | X | X |  |  |  |
|  | *Baptistonia echinata* Barb.Rodr. | Ep |  | X | X |  |  |  |
|  | *Baptistonia gutfreundiana* (Chiron & V.P.Castro) Chiron & V.P.Castro | Ep |  | X | X |  |  |  |
|  | *Baptistonia kautskyi* (Pabst) Chiron & V.P.Castro | Ep | EN | X | X |  |  |  |
|  | *Baptistonia leinigii* (Pabst) Chiron & V.P.Castro | Ep |  | X | X |  |  |  |
|  | *Baptistonia lietzei* (Regel) Chiron & V.P.Castro | Ep |  |  | X |  |  |  |
|  | *Baptistonia nitida* (Barb.Rodr.) Chiron & V.P.Castro | Ep |  | X | X |  |  |  |
|  | *Baptistonia pabstii* (Campacci & C.Espejo) Chiron & V.P.Castro | Ep |  | X | X |  |  |  |
|  | *Baptistonia pubes* (Lindl.) Chiron & V.P.Castro | Ep |  | X | X |  |  |  |
|  | *Baptistonia pulchella* (Regel) Chiron & V.P.Castro | Ep |  | X | X |  |  |  |
|  | *Baptistonia riograndensis* (Cogn.) Chiron & V.P.Castro | Ep |  | X | X |  |  |  |
|  | *Baptistonia sarcodes* (Lindl.) van den Berg & M.W.Chase | Ep |  | X | X |  |  |  |
|  | *Baptistonia silvana* (V.P.Castro & Campacci) V.P.Castro & Chiron | Ep |  | X | X |  |  |  |
|  | *Baptistonia truncata* (Pabst) Chiron & V.P.Castro | Ep | CR | X | X |  |  |  |
|  | *Baptistonia uhlii* Chiron & V.P.Castro | Ep |  | X | X |  |  |  |
|  | *Baptistonia velteriana* V.P.Castro & Chiron | Ep |  | X | X |  |  |  |
|  | *Baptistonia venusta* (Drapiez) Chiron | Ep |  | X | X |  |  |  |
|  | *Baptistonia widgrenii* (Lindl.) V.P.Castro & Chiron | Ep |  | X | X |  |  |  |
|  | *Baptistonia x amicta* (Lindl.) Chiron & V.P.Castro | Ep |  |  | X |  |  |  |
|  | *Barbosella australis* (Cogn.) Schltr. | Ep |  | X | X |  |  |  |
|  | *Barbosella cogniauxiana* (Speg. & Kraenzl.) Schltr. | Ep |  |  | X |  |  |  |
|  | *Barbosella crassifolia* (Edwall) Schltr. | Ep |  | X | X |  | X | X |
|  | *Barbosella dusenii* (A.Samp.) Schltr. | Ep |  | X | X |  |  |  |
|  | *Barbosella gardneri* (Lindl.) Schltr. | Ep |  | X | X |  |  |  |
|  | *Barbosella macaheensis* (Cogn.) Luer | Ep |  | X | X |  |  |  |
|  | *Barbosella miersii* (Lindl.) Schltr. | Ep |  | X | X |  |  |  |
|  | *Barbosella spiritusanctensis* (Pabst) F.Barros & Toscano | Ep |  | X | X |  |  |  |
|  | *Barbosella trilobata* Pabst | Ep | EN | X | X |  |  |  |
|  | *Bifrenaria atropurpurea* Lindl. | Ep |  | X | X |  |  |  |
|  | *Bifrenaria aureofulva* (Hook.) Lindl. | Ep |  | X | X |  | X | X |
|  | *Bifrenaria calcarata* Barb.Rodr. | Ep |  | X | X |  |  |  |
|  | *Bifrenaria charlesworthii* Rolfe | Ep |  | X | X |  |  |  |
|  | *Bifrenaria clavigera* Rchb.f. | Ep |  | X | X |  |  |  |
|  | *Bifrenaria harrisoniae* (Hook.) Rchb.f. | Ep |  | X | X |  |  |  |
|  | *Bifrenaria inodora* Lindl. | Ep |  | X | X |  |  |  |
|  | *Bifrenaria leucorrhoda* Rchb.f. | Ep |  | X | X |  |  |  |
|  | *Bifrenaria mellicolor* Rchb.f. | Ep |  | X | X |  |  |  |
|  | *Bifrenaria racemosa* (Hook.) Lindl. | Ep |  | X | X |  |  |  |
|  | *Bifrenaria silvana* V.P.Castro | Ep | CR | X | X |  |  |  |
|  | *Bifrenaria stefanae* V.P.Castro | Ep |  | X | X |  |  |  |
|  | *Bifrenaria tetragona* (Lindl.) Schltr. | Ep |  | X | X |  |  |  |
|  | *Bifrenaria vitellina* (Lindl.) Lindl. | Ep |  | X | X |  |  |  |
|  | *Bifrenaria wittigii* (Rchb.f.) Hoehne | Ep | EN | X | X |  |  |  |
|  | *Brachionidium restrepioides* (Hoehne) Pabst | Ep | VU | X | X |  |  |  |
|  | *Brasilaelia crispa* (Lindl.) Campacci | Ep | VU | X | X |  |  |  |
|  | *Brasilaelia fidelensis* (Pabst) Gutfreund | Ep |  | X | X |  |  |  |
|  | *Brasilaelia grandis* (Lindl. & Paxton) Gutfreund | Ep | VU | X | X |  |  |  |
|  | *Brasilaelia lobata* (Lindl.) Gutfreund | Ep | EN | X | X |  |  |  |
|  | *Brasilaelia perrinii* (Lindl.) Campacci | Ep | VU | X | X |  |  |  |
|  | *Brasilaelia purpurata* (Lindl. & Paxton) Campacci | Ep | VU | X | X |  |  |  |
|  | *Brasilaelia tenebrosa* (Rolfe) Campacci | Ep | EN | X | X |  |  |  |
|  | *Brasilaelia virens* (Lindl.) Campacci | Ep |  | X | X |  |  |  |
|  | *Brasilaelia xanthina* (Lindl.) Campacci | Ep | EN | X | X |  |  |  |
|  | *Brasilidium concolor* (Hook.) F.Barros & V.T.Rodrigues | Ep |  |  | X |  |  |  |
|  | *Brasilidium crispum* (Lodd.) Campacci | Ep |  | X | X |  |  |  |
|  | *Brasilidium curtum* (Lindl.) Campacci | Ep |  | X | X |  |  |  |
|  | *Brasilidium dasytyle* (Rchb.f.) F.Barros & V.T.Rodrigues | Ep |  | X | X |  |  |  |
|  | *Brasilidium forbesii* (Hook.) Campacci | Ep |  | X | X |  |  |  |
|  | *Brasilidium gardneri* (Lindl.) Campacci | Ep |  | X | X |  |  |  |
|  | *Brasilidium gravesianum* (Rolfe) Campacci | Ep |  | X | X |  | X |  |
|  | *Brasilidium marshallianum* (Rchb.f.) Campacci | Ep | CR | X | X |  |  |  |
|  | *Brasilidium pectorale* (Lindl.) Campacci | Ep | CR | X | X |  |  |  |
|  | *Brasilidium praetextum* (Rchb.f.) Campacci | Ep |  | X | X |  |  |  |
|  | *Brasilidium zappii* (Pabst) Campacci | Ep |  | X | X |  |  |  |
|  | *Brasiliorchis barbosae* (Loefgr.) R.Singer et al. | Ep |  | X | X |  |  |  |
|  | *Brasiliorchis chrysantha* (Barb.Rodr.) R.Singer et al. | Ep |  |  | X |  |  |  |
|  | *Brasiliorchis consanguinea* (Klotzsch) R.Singer et al. | Ep |  | X | X |  |  |  |
|  | *Brasiliorchis gracilis* (Lodd.) R.Singer et al. | Ep |  | X | X |  |  |  |
|  | *Brasiliorchis heismanniana* (Barb.Rodr.) R.B.Singer et al. | Ep |  | X | X |  |  |  |
|  | *Brasiliorchis kautskyi* (Pabst) R.Singer et al. | Ep |  | X | X |  |  |  |
|  | *Brasiliorchis marginata* (Lindl.) R.Singer et al. | Ep |  |  | X |  |  |  |
|  | *Brasiliorchis phoenicanthera* (Barb.Rodr.) R.Singer et al. | Ep |  | X | X |  |  |  |
|  | *Brasiliorchis picta* (Hook.) R.Singer et al. | Ep |  |  | X |  |  |  |
|  | *Brasiliorchis polyantha* (Barb.Rodr.) R.Singer et al. | Ep |  | X | X |  |  |  |
|  | *Brasiliorchis porphyrostele* (Rchb.f.) R.Singer et al. | Ep |  | X | X |  |  |  |
|  | *Brasiliorchis schunkeana* (Campacci & Kautsky) R.Singer et al. | Ep | EN | X | X |  |  |  |
|  | *Brasiliorchis ubatubana* (Hoehne) R.Singer et al. | Ep |  | X | X |  |  |  |
|  | *Brassavola flagellaris* Barb.Rodr. | Ep |  | X | X | X | X |  |
|  | *Brassavola reginae* Pabst | Ep |  | X | X |  |  |  |
|  | *Brassavola rhomboglossa* Pabst | Ep |  |  | X |  | X |  |
|  | *Brassavola tuberculata* Hook. | Ep |  |  | X |  | X | X |
|  | *Brassia arachnoidea* Barb.Rodr. | Ep | VU | X | X | X |  |  |
|  | *Bulbophyllum arianeae* Fraga & E.C.Smidt | Ep | CR | X | X |  |  |  |
|  | *Bulbophyllum atropurpureum* Barb.Rodr. | Ep |  | X | X |  |  |  |
|  | *Bulbophyllum barbatum* Barb.Rodr. | Ep |  | X | X |  | X |  |
|  | *Bulbophyllum boudetianum* Fraga | Ep | EN | X | X |  |  |  |
|  | *Bulbophyllum campos-portoi* Brade | Ep |  | X | X |  |  |  |
|  | *Bulbophyllum cantagallense* (Barb.Rodr.) Cogn. | Ep |  | X | X |  |  |  |
|  | *Bulbophyllum carassense* R.C.Mota et al. | Ep |  | X | X |  |  |  |
|  | *Bulbophyllum chloroglossum* Rchb.f. & Warm. | Ep |  | X | X |  | X |  |
|  | *Bulbophyllum chloropterum* Rchb.f. | Ep |  | X | X |  | X |  |
|  | *Bulbophyllum dracuzzi* Campacci | Ep |  | X | X |  |  |  |
|  | *Bulbophyllum dusenii* Kraenzl. | Ep |  | X | X |  | X |  |
|  | *Bulbophyllum epiphytum* (Barb.Rodr.) Cogn. | Ep |  |  | X |  | X |  |
|  | *Bulbophyllum exaltatum* Lindl. | Ep |  |  | X |  | X | X |
|  | *Bulbophyllum gehrtii* E.C.Smidt & Borba | Ep |  | X | X |  | X |  |
|  | *Bulbophyllum gladiatum* Lindl. | Ep |  |  | X |  | X |  |
|  | *Bulbophyllum glutinosum* (Barb.Rodr.) Cogn. | Ep |  | X | X |  |  |  |
|  | *Bulbophyllum granulosum* Barb.Rodr. | Ep |  | X | X |  | X |  |
|  | *Bulbophyllum kautskyi* Toscano | Ep | VU | X | X |  |  |  |
|  | *Bulbophyllum macroceras* Barb.Rodr. | Ep |  | X | X |  |  |  |
|  | *Bulbophyllum malachadenia* Cogn. | Ep |  | X | X |  |  |  |
|  | *Bulbophyllum meridense* Rchb.f. | Ep |  |  | X |  |  |  |
|  | *Bulbophyllum micranthum* Barb.Rodr. | Ep |  | X | X |  | X |  |
|  | *Bulbophyllum micropetaliforme* J.E.Leite | Ep |  | X | X |  |  |  |
|  | *Bulbophyllum napellii* Lindl. | Ep |  | X | X |  | X |  |
|  | *Bulbophyllum perii* Schltr. | Ep |  | X | X |  | X |  |
|  | *Bulbophyllum pitengoense* Campacci | Ep |  | X | X |  | X |  |
|  | *Bulbophyllum plumosum* (Barb.Rodr.) Cogn. | Ep |  | X | X |  | X | X |
|  | *Bulbophyllum regnellii* Rchb.f. | Ep |  |  | X |  | X |  |
|  | *Bulbophyllum teimosense* E.C.Smidt & Borba | Ep |  | X | X |  |  |  |
|  | *Bulbophyllum tripetalum* Lindl. | Ep |  |  | X |  | X |  |
|  | *Camaridium carinatum* (Barb.Rodr.) Hoehne | Ep |  |  | X |  |  |  |
|  | *Camaridium micranthum* M.A.Blanco | Ep |  |  | X | X |  |  |
|  | *Camaridium ochroleucum* Lindl. | Ep |  |  | X | X | X |  |
|  | *Campylocentrum aciculatum* (Rchb.f. & Warm.) Cogn. | Ep |  | X | X |  | X | X |
|  | *Campylocentrum aromaticum* Barb.Rodr. | Ep |  |  | X |  | X |  |
|  | *Campylocentrum brachycarpum* Cogn. | Ep |  | X | X |  |  |  |
|  | *Campylocentrum crassirhizum* Hoehne | Ep |  | X | X |  |  | X |
|  | *Campylocentrum densiflorum* Cogn. | Ep |  |  | X |  |  |  |
|  | *Campylocentrum gracile* Cogn. | Ep |  | X | X |  |  |  |
|  | *Campylocentrum grisebachii* Cogn. | Ep |  |  | X | X |  |  |
|  | *Campylocentrum hirtellum* Cogn. | Ep |  | X | X |  |  |  |
|  | *Campylocentrum iglesiasii* Brade | Ep |  | X | X |  |  |  |
|  | *Campylocentrum lansbergii* (Rchb.f.) Schltr. | Ep |  |  | X | X |  |  |
|  | *Campylocentrum linearifolium* Cogn. | Ep |  | X | X |  | X | X |
|  | *Campylocentrum micranthum* (Lindl.) Rolfe | Ep |  |  | X | X | X | X |
|  | *Campylocentrum neglectum* (Rchb.f. & Warm.) Cogn. | Ep |  |  | X |  | X | X |
|  | *Campylocentrum organense* (Rchb.f.) Rolfe | Ep |  | X | X |  |  |  |
|  | *Campylocentrum ornithorhynchum* (Lindl.) Rolfe | Ep |  | X | X |  |  |  |
|  | *Campylocentrum parahybunense* (Barb.Rodr.) Rolfe | Ep |  | X | X |  |  |  |
|  | *Campylocentrum pauloense* Schltr. & Hoehne | Ep |  |  | X |  |  |  |
|  | *Campylocentrum pernambucense* Hoehne | Ep | EN | X | X |  |  |  |
|  | *Campylocentrum pubirhachys* Schltr. | Ep |  | X | X |  |  |  |
|  | *Campylocentrum robustum* Cogn. | Ep |  | X | X | X | X |  |
|  | *Campylocentrum sellowii* (Rchb.f.) Rolfe | Ep |  | X | X | X |  |  |
|  | *Campylocentrum spannagelii* Hoehne | Ep |  | X | X |  |  |  |
|  | *Campylocentrum ulei* Cogn. | Ep |  |  | X |  |  |  |
|  | *Campylocentrum wawrae* (Rchb.f. ex Beck) Rolfe | Ep |  | X | X |  |  |  |
|  | *Capanemia adelaidae* Brade | Ep |  | X | X |  |  |  |
|  | *Capanemia angustilabia* Schltr. | Ep |  | X | X |  |  |  |
|  | *Capanemia australis* (Kraenzl.) Schltr. | Ep |  |  | X |  |  |  |
|  | *Capanemia carinata* Barb.Rodr. | Ep |  | X | X |  |  |  |
|  | *Capanemia fluminensis* Pabst | Ep |  | X | X |  |  |  |
|  | *Capanemia gehrtii* Hoehne | Ep |  | X | X |  | X |  |
|  | *Capanemia lossiana* L.Kollmann | Ep |  | X | X |  |  |  |
|  | *Capanemia micromera* Barb.Rodr. | Ep |  |  | X |  |  |  |
|  | *Capanemia paranaensis* Schltr. | Ep |  | X | X |  |  |  |
|  | *Capanemia riograndensis* Pabst | Ep |  | X | X |  |  |  |
|  | *Capanemia spathuliglossa* Pabst | Ep |  | X | X |  |  |  |
|  | *Capanemia superfula* (Rchb.f.) Garay | Ep |  |  | X |  |  |  |
|  | *Capanemia thereziae* Barb.Rodr. | Ep |  | X | X |  | X |  |
|  | *Catasetum arietinum* F.E.L.Miranda & K.G.Lacerda | Ep |  | X | X |  |  |  |
|  | *Catasetum atratum* Lindl. | Ep |  | X | X |  | X |  |
|  | *Catasetum blackii* Pabst | Ep |  | X | X |  | X | X |
|  | *Catasetum cernuum* (Lindl.) Rchb.f. | Ep |  | X | X |  | X |  |
|  | *Catasetum discolor* Lindl. | Ep |  |  | X | X |  |  |
|  | *Catasetum fimbriatum* Lindl. | Ep |  |  | X |  | X |  |
|  | *Catasetum globiflorum* Hook. | Ep |  | X | X |  |  |  |
|  | *Catasetum hookeri* Lindl. | Ep |  | X | X | X |  | X |
|  | *Catasetum joaquianum* Campacci & G.F.Carr | Ep |  | X | X |  |  |  |
|  | *Catasetum labiatum* Barb.Rodr. | Ep |  | X | X |  |  |  |
|  | *Catasetum luridum* Lindl. | Ep |  | X | X |  |  | X |
|  | *Catasetum macrocarpum* Rich. ex Kunth | Ep |  |  | X | X |  | X |
|  | *Catasetum mattosianum* Bicalho | Ep | EN | X | X |  |  |  |
|  | *Catasetum micranthum* Barb.Rodr. | Ep |  | X | X |  |  |  |
|  | *Catasetum purum* Nees & Sinnings | Ep |  | X | X |  |  |  |
|  | *Catasetum socco* (Vell.) Hoehne | Ep |  | X | X |  |  |  |
|  | *Catasetum triodon* Rchb.f. | Ep |  | X | X |  |  |  |
|  | *Catasetum uncatum* Rolfe | Ep |  | X | X |  |  |  |
|  | *Cattleya aclandiae* Lindl. | Ep | VU | X | X |  |  |  |
|  | *Cattleya amethystoglossa* Linden & Rchb.f. | Ep |  | X | X |  |  |  |
|  | *Cattleya bicolor* Lindl. | Ep |  | X | X |  | X |  |
|  | *Cattleya dormaniana* Rchb.f. | Ep | EN | X | X |  |  |  |
|  | *Cattleya forbesii* Lindl. | Ep |  | X | X |  |  |  |
|  | *Cattleya granulosa* Veitch | Ep | VU | X | X |  |  |  |
|  | *Cattleya guttata* Lindl. | Ep | VU | X | X |  |  |  |
|  | *Cattleya harrisoniana* Batem. ex Lindl. | Ep | VU | X | X |  |  |  |
|  | *Cattleya intermedia* Graham | Ep | VU | X | X |  |  |  |
|  | *Cattleya kerrii* Brieger & Bicalho | Ep |  | X | X |  |  |  |
|  | *Cattleya labiata* Lindl. | Ep | VU | X | X |  |  | X |
|  | *Cattleya loddigesii* Lindl. | Ep |  | X | X |  | X |  |
|  | *Cattleya porphyroglossa* Linden & Rchb.f. | Ep | CR | X | X |  |  |  |
|  | *Cattleya schilleriana* Rchb.f. | Ep | EN | X | X |  |  |  |
|  | *Cattleya schofieldiana* Rchb.f. | Ep | CR | X | X |  |  |  |
|  | *Cattleya tenuis* Campacci & Vedovello | Ep | EN |  | X |  | X | X |
|  | *Cattleya tigrina* A.Rich. | Ep | VU | X | X |  |  |  |
|  | *Cattleya velutina* Rchb.f. | Ep | VU | X | X |  |  |  |
|  | *Cattleya walkeriana* Gardner | Ep | VU | X | X | X | X |  |
|  | *Cattleya warneri* T.Moore | Ep | VU | X | X |  |  |  |
|  | *Centroglossa aurantiaca* Chiron & N.Sanson | Ep |  | X | X |  |  |  |
|  | *Centroglossa castellensis* Brade | Ep | CR | X | X |  |  |  |
|  | *Centroglossa greeniana* (Rchb.f.) Cogn. | Ep |  | X | X |  |  |  |
|  | *Centroglossa macroceras* Rchb.f. | Ep |  | X | X |  |  |  |
|  | *Centroglossa nuneslimae* Porto & Brade | Ep |  | X | X |  |  |  |
|  | *Centroglossa tripollinica* Barb.Rodr. | Ep |  | X | X |  |  |  |
|  | *Christensonella echinophyta* (Barb.Rodr.) Szlach. et al. | Ep |  | X | X |  |  |  |
|  | *Christensonella ferdinandiana* (Barb.Rodr.) Szlach. et al. | Ep |  | X | X |  |  |  |
|  | *Christensonella neuwiedii* (Rchb.f.) S.Koehler | Ep |  | X | X |  |  |  |
|  | *Christensonella pachyphylla* (Schltr. ex Hoehne) Szlach. et al. | Ep |  | X | X |  |  |  |
|  | *Christensonella paranaensis* (Barb.Rodr.) S.Koehler | Ep |  | X | X |  |  |  |
|  | *Christensonella pumila* (Hook.) Szlach. et al. | Ep |  |  | X | X |  |  |
|  | *Christensonella subulata* (Lindl.) Szlach. et al. | Ep |  | X | X |  | X |  |
|  | *Christensonella vernicosa* (Barb.Rodr.) Szlach. et al. | Ep |  | X | X |  |  |  |
|  | *Chytroglossa aurata* Rchb.f. | Ep |  | X | X |  |  |  |
|  | *Chytroglossa marileoniae* Rchb.f. | Ep |  | X | X |  |  |  |
|  | *Chytroglossa paulensis* Edwall | Ep |  | X | X |  |  |  |
|  | *Cirrhaea dependens* Rchb.f. | Ep |  | X | X |  |  |  |
|  | *Cirrhaea fuscolutea* Lindl. | Ep | EN | X | X |  |  |  |
|  | *Cirrhaea loddigesii* Lindl. | Ep | CR | X | X |  |  |  |
|  | *Cirrhaea longiracemosa* Hoehne | Ep | VU | X | X |  |  |  |
|  | *Cirrhaea nasuta* Brade | Ep |  | X | X |  |  |  |
|  | *Cirrhaea seidelii* Pabst | Ep |  | X | X |  |  |  |
|  | *Cirrhaea silvana* V.P.Castro & Campacci | Ep |  | X | X |  |  |  |
|  | *Cochleanthes flabelliformis* (Sw.) Schult. & Garay | Ep |  |  | X |  |  |  |
|  | *Comparettia coccinea* Lindl. | Ep |  | X | X |  | X |  |
|  | *Comparettia falcata* Poepp. & Endl. | Ep |  |  | X |  |  |  |
|  | *Constantia australis* (Cogn.) Porto & Brade | Ep |  | X | X |  |  |  |
|  | *Constantia rupestris* Barb.Rodr. | Ep |  | X | X |  |  |  |
|  | *Coppensia bifolia* (Sims) Dumort | Ep |  |  | X |  | X |  |
|  | *Coppensia edmundoi* (Pabst) Campacci | Ep |  | X | X |  |  |  |
|  | *Coppensia flexuosa* (Sims) Campacci | Ep |  |  | X |  |  |  |
|  | *Coppensia hookeri* (Rolfe) F.Barros & L.Guimarães | Ep |  | X | X |  |  |  |
|  | *Coppensia loefgrenii* (Cogn.) F.Barros & V.T.Rodrigues | Ep |  | X | X |  |  |  |
|  | *Coppensia longicorna* (Mutel) F.Barros & V.T.Rodrigues | Ep |  | X | X |  |  |  |
|  | *Coppensia macronyx* (Rchb.f.) F.Barros & V.T.Rodrigues | Ep | VU |  | X |  |  |  |
|  | *Coppensia majevskyi* (Toscano & V.P.Castro) Campacci | Hep | EN | X | X |  |  |  |
|  | *Coppensia ouricanensis* (V.P.Castro & Campacci) F.Barros & V.T.Rodrigues | Ep |  | X | X |  |  |  |
|  | *Coppensia paranaensis* (Kraenzl.) F.Barros & V.T.Rodrigues | Ep |  |  | X |  |  |  |
|  | *Coppensia paranana* (M.W.Chase & N.H.Williams) F.Barros & V.T.Rodrigues | Ep |  | X | X |  |  |  |
|  | *Coppensia ranifera* (Lindl.) F.Barros & V.T.Rodrigues | Ep |  | X | X |  |  |  |
|  | *Coppensia varicosa* (Lindl.) Campacci | Ep |  | X | X |  | X |  |
|  | *Coppensia warmingii* (Rchb.f.) Campacci | Ep |  |  | X |  | X |  |
|  | *Coppensia welteri* (Pabst) Campacci | Ep |  | X | X |  |  |  |
|  | *Coryanthes bueraremensis* Campacci & Bohnke | Ep |  | X | X |  |  |  |
|  | *Coryanthes speciosa* Hook. | Ep |  |  | X | X |  |  |
|  | *Cryptarrhena brasiliensis* Brade | Ep |  | X | X |  |  |  |
|  | *Cryptarrhena guatemalensis* Schltr. | Ep |  |  | X |  |  |  |
|  | *Cyclopogon multiflorus* Schltr. | Ep |  | X | X |  |  |  |
|  | *Cycnoches pentadactylum* Lindl. | Ep | EN | X | X |  | X |  |
|  | *Cyrtopodium gigas* (Vell.) Hoehne | Ep |  | X | X |  |  |  |
|  | *Cyrtopodium palmifrons* Rchb.f. & Warm. | Ep | VU |  | X |  | X |  |
|  | *Dichaea anchorifera* Rchb.f. | Ep |  | X | X |  |  |  |
|  | *Dichaea australis* Cogn. | Ep |  | X | X |  |  |  |
|  | *Dichaea brevicaulis* Cogn. | Ep |  | X | X |  |  |  |
|  | *Dichaea bryophyla* Rchb.f. | Ep |  | X | X |  | X |  |
|  | *Dichaea cogniauxiana* Schltr. | Ep |  | X | X |  | X |  |
|  | *Dichaea elianae* Xim.Bols. | Ep |  | X | X |  |  |  |
|  | *Dichaea mosenii* Cogn. | Ep | VU | X | X |  |  |  |
|  | *Dichaea pendula* (Aubl.) Cogn. | Ep |  |  | X | X |  |  |
|  | *Dichaea trulla* Rchb.f. | Ep |  |  | X | X |  |  |
|  | *Dimerandra emarginata* (G.Mey.) Hoehne | Ep |  |  | X | X |  |  |
|  | *Dryadella ataleiensis* Campacci | Ep |  | X | X |  |  |  |
|  | *Dryadella auriculigera* (Rchb.f.) Luer | Ep | CR | X | X |  |  |  |
|  | *Dryadella aviceps* (Rchb.f.) Luer | Ep |  |  | X |  |  |  |
|  | *Dryadella crenulata* (Pabst) Luer | Ep |  | X | X |  |  |  |
|  | *Dryadella edwallii* (Cogn.) Luer | Ep |  | X | X |  |  |  |
|  | *Dryadella espiritosantensis* (Pabst) Luer | Ep |  | X | X |  |  |  |
|  | *Dryadella gomesferreirae* (Pabst) Luer | Ep |  | X | X |  |  |  |
|  | *Dryadella kautskyi* (Pabst) Luer | Ep |  | X | X |  |  |  |
|  | *Dryadella liliputiana* (Cogn.) Luer | Ep | VU | X | X |  |  |  |
|  | *Dryadella litoralis* Campacci | Ep |  | X | X |  |  |  |
|  | *Dryadella susanae* (Pabst) Luer | Ep | CR | X | X |  |  |  |
|  | *Dryadella toscanoi* Luer | Ep |  | X | X |  |  |  |
|  | *Dryadella vitorinoi* Luer & Toscano | Ep |  | X | X |  |  |  |
|  | *Dryadella zebrina* (Porsch) Luer | Ep |  |  | X |  |  |  |
|  | *Dungsia harpophylla* (Rchb.f.) Chiron & V.P.Castro | Ep | VU | X | X |  |  |  |
|  | *Dungsia kautskyi* (Pabst) Chiron & V.P.Castro | Ep | CR | X | X |  |  |  |
|  | *Elleanthus brasiliensis* Rchb.f. | Ep |  |  | X |  |  |  |
|  | *Elleanthus crinipes* Rchb.f. | Ep |  | X | X |  | X |  |
|  | *Elleanthus hymenophorus* Rchb.f. | Ep |  |  | X |  |  |  |
|  | *Elleanthus linifolius* C.Presl | Ep |  |  | X | X |  |  |
|  | *Encyclia advena* (Rchb.f.) Porto & Brade | Ep |  | X | X |  | X |  |
|  | *Encyclia andrichii* L.C.Menezes | Ep |  | X | X |  |  |  |
|  | *Encyclia bonhkiana* V.P.Castro & Campacci | Ep |  | X | X |  |  |  |
|  | *Encyclia bracteata* (Barb.Rodr.) Schltr. | Ep |  | X | X |  |  |  |
|  | *Encyclia bragancae* Ruschi | Ep | EN | X | X |  |  |  |
|  | *Encyclia conchaechila* (Barb.Rodr.) Porto & Brade | Ep |  |  | X | X | X |  |
|  | *Encyclia cordigera* (Kunth) Dressler | Ep |  |  | X |  |  |  |
|  | *Encyclia cyperifolia* (C.Schweinf.) Carnevali & I.Ramírez | Ep |  |  | X | X |  |  |
|  | *Encyclia dichroma* (Lindl.) Schltr. | Ep |  | X | X |  |  |  |
|  | *Encyclia euosma* (Rchb.f.) Porto & Brade | Ep |  | X | X |  | X |  |
|  | *Encyclia fimbriatum* C.A.Bastos et al. | Ep |  | X | X |  |  |  |
|  | *Encyclia fowliei* Duveen | Ep |  | X | X |  |  |  |
|  | *Encyclia gallopavina* (Rchb.f.) Porto & Brade | Ep |  | X | X | X | X |  |
|  | *Encyclia marxiana* Campacci | Ep |  | X | X |  |  |  |
|  | *Encyclia oncidioides* (Lindl.) Schltr. | Ep |  |  | X | X | X | X |
|  | *Encyclia osmantha* (Barb.Rodr.) Schltr. | Ep |  | X | X |  | X |  |
|  | *Encyclia patens* Hook. | Ep |  | X | X |  | X |  |
|  | *Encyclia pauciflora* (Barb.Rodr.) Porto & Brade | Ep |  | X | X |  | X |  |
|  | *Encyclia silvana* V.P.Castro & Campacci | Ep |  | X | X |  |  |  |
|  | *Encyclia unaensis* Fowlie | Ep |  | X | X |  |  |  |
|  | *Encyclia zaslawskiana* Campacci | Ep |  | X | X |  |  |  |
|  | *Epidendrum addae* Pabst | Ep | VU | X | X |  |  |  |
|  | *Epidendrum ammophilum* Barb.Rodr. | Ep |  | X | X |  |  |  |
|  | *Epidendrum anatipedium* L.M.Sánchez & Hágsater | Ep |  | X | X |  |  |  |
|  | *Epidendrum* *anceps* Jacq. | Ep |  |  | X | X | X |  |
|  | *Epidendrum armeniacum* Lindl. | Ep |  |  | X |  |  |  |
|  | *Epidendrum avicula* Lindl. | Ep |  |  | X |  | X | X |
|  | *Epidendrum berkeleyi* (Rolfe) Baptista | Ep |  | X | X |  | X |  |
|  | *Epidendrum caldense* Barb.Rodr. | Ep |  | X | X |  |  |  |
|  | *Epidendrum campaccii* Hágsater & L.M.Sánchez | Ep |  | X | X |  |  |  |
|  | *Epidendrum carpophorum* Barb.Rodr | Ep |  |  | X | X |  | X |
|  | *Epidendrum carvalhoi* Toscano | Ep |  | X | X |  |  |  |
|  | *Epidendrum chlorinum* Barb.Rodr. | Ep |  | X | X |  | X |  |
|  | *Epidendrum cooperianum* Batem. | Ep |  | X | X |  |  |  |
|  | *Epidendrum coronatum* Ruiz & Pav. | Ep |  | X | X | X |  |  |
|  | *Epidendrum cristatum* Ruiz & Pav. | Ep |  |  | X |  | X | X |
|  | *Epidendrum densiflorum* Lindl. | Ep |  |  | X | X | X |  |
|  | *Epidendrum denticulatum* Barb.Rodr. | Ep |  | X | X |  | X | X |
|  | *Epidendrum ecostatum* Pabst | Ep | VU |  | X |  |  |  |
|  | *Epidendrum filicaule* (Sw.) Lindl. | Ep |  | X | X |  |  |  |
|  | *Epidendrum flexuosum* G.Mey. | Ep |  |  | X | X | X |  |
|  | *Epidendrum forcipatoides* Hágsater | Ep |  | X | X |  |  |  |
|  | *Epidendrum garciae* Pabst | Ep |  | X | X |  |  |  |
|  | *Epidendrum geniculatum* Barb.Rodr. | Ep |  | X | X |  |  |  |
|  | *Epidendrum goebelii* Schltr. | Ep |  | X | X |  |  |  |
|  | *Epidendrum harrisoniae* Hook. | Ep |  | X | X |  |  |  |
|  | *Epidendrum henschenii* Barb.Rodr. | Ep | EN | X | X |  |  |  |
|  | *Epidendrum hololeucum* Barb.Rodr. | Ep |  | X | X |  |  |  |
|  | *Epidendrum imatophyllum* Lindl. | Ep |  | X | X |  |  |  |
|  | *Epidendrum infaustum* Rchb.f. | Ep |  | X | X |  |  |  |
|  | *Epidendrum kautskyi* Pabst | Ep |  | X | X |  |  |  |
|  | *Epidendrum latilabre* Lindl. | Ep |  | X | X | X |  |  |
|  | *Epidendrum loefgrenii* Cogn. | Ep |  | X | X |  |  |  |
|  | *Epidendrum macrocarpum* Rich. | Ep |  |  | X | X |  |  |
|  | *Epidendrum magdalenense* Porto & Brade | Ep |  | X | X |  |  |  |
|  | *Epidendrum mantiqueiranum* Porto & Brade | Ep |  | X | X |  |  |  |
|  | *Epidendrum nocturnum* Jacq. | Ep |  |  | X | X | X |  |
|  | *Epidendrum nutans* Sw. | Ep |  | X | X |  |  |  |
|  | *Epidendrum obergii* Hawkes | Ep |  | X | X |  |  |  |
|  | *Epidendrum ochrochlorum* Barb.Rodr. | Ep |  | X | X |  | X | X |
|  | *Epidendrum paniculosum* Barb.Rodr. | Ep |  | X | X |  |  |  |
|  | *Epidendrum paranaense* Barb.Rodr. | Ep |  | X | X |  | X | X |
|  | *Epidendrum patens* Sw. | Ep |  |  | X | X |  |  |
|  | *Epidendrum polyanthum* Lindl. | Ep |  |  | X |  | X |  |
|  | *Epidendrum proligerum* Barb.Rodr. | Ep |  | X | X |  |  |  |
|  | *Epidendrum pseudodifforme* Hoehne & Schltr. | Ep |  | X | X |  |  |  |
|  | *Epidendrum ramosum* Jacq. | Ep |  | X | X | X | X |  |
|  | *Epidendrum rigidum* Jacq. | Ep |  |  | X | X | X | X |
|  | *Epidendrum robustum* Cogn. | Ep | VU | X | X |  |  |  |
|  | *Epidendrum rodriguesii* Cogn. | Ep |  |  | X | X |  |  |
|  | *Epidendrum rupicolum* Cogn. | Ep |  | X | X |  |  |  |
|  | *Epidendrum saxatile* Lindl. | Ep |  |  | X |  | X | X |
|  | *Epidendrum saximontanum* Pabst | Ep |  | X | X |  |  |  |
|  | *Epidendrum secundum* Jacq. | Ep |  |  | X | X | X | X |
|  | *Epidendrum strobiliferum* Rchb.f. | Ep |  |  | X | X | X |  |
|  | *Epidendrum successivum* Hágsater & F.E.L.Miranda | Ep |  | X | X |  |  |  |
|  | *Epidendrum tridactylum* Lindl. | Ep |  | X | X |  |  | X |
|  | *Epidendrum vesicatum* Lindl. | Ep |  | X | X |  |  |  |
|  | *Epidendrum waiandtii* V.P.Castro | Ep |  | X | X |  |  |  |
|  | *Epidendrum xanthinum* Lindl. | Ep |  |  | X | X |  | X |
|  | *Epidendrum zappii* Pabst | Ep | EN | X | X |  |  |  |
|  | *Erycina glossomistax* (Rchb.f.) N.H.Williams & M.W.Chase | Ep |  |  | X | X |  |  |
|  | *Erycina pumilio* (Rchb.f.) N.H.Williams & M.W.Chase | Ep |  |  | X | X |  |  |
|  | *Erycina pusilla* (L.) N.H.Williams & M.W.Chase | Ep |  |  | X | X |  |  |
|  | *Eurystyles actinosophila* (Barb.Rodr.) Schltr. | Ep |  |  | X |  | X |  |
|  | *Eurystyles cogniauxii* (Kraenzl.) Pabst | Ep |  | X | X |  |  |  |
|  | *Eurystyles cotyleton* Wawra | Ep |  | X | X |  |  |  |
|  | *Eurystyles crocodilus* Szlach. | Ep |  | X | X |  |  |  |
|  | *Eurystyles gardneri* (Lindl. ex Gardner) Garay | Ep |  | X | X |  |  |  |
|  | *Eurystyles hoehnei* Szlach. | Ep |  | X | X |  |  |  |
|  | *Eurystyles lobata* Chiron & V.P.Castro | Ep |  | X | X |  |  |  |
|  | *Eurystyles lorenzii* (Cogn.) Schltr. | Ep |  | X | X |  |  |  |
|  | *Eurystyles rutkowskiana* Szlach. | Ep |  | X | X |  |  |  |
|  | *Eurystyles splendissima* Szlach. | Ep |  | X | X |  |  |  |
|  | *Galeandra stangeana* Rchb.f. | Ep |  |  | X | X |  |  |
|  | *Galeottia ciliata* (Morel) Dressler & Christenson | Ep |  |  | X | X | X |  |
|  | *Gomesa alpina* Porsch | Ep |  | X | X |  |  |  |
|  | *Gomesa barkeri* (Hook.) Regel | Ep |  | X | X |  |  |  |
|  | *Gomesa brasiliensis* (Rolfe) M.W.Chase & N.H.Williams | Ep |  | X | X |  |  |  |
|  | *Gomesa crispa* (Lindl.) Klotzsch & Rchb.f. | Ep |  |  | X |  |  |  |
|  | *Gomesa divaricata* Hoffmanns. ex Schltr. | Ep |  | X | X |  |  |  |
|  | *Gomesa doeringii* (Hoehne) Pabst | Ep |  |  | X |  |  |  |
|  | *Gomesa duseniana* Kraenzl. | Ep |  | X | X |  |  |  |
|  | *Gomesa fischeri* Regel | Ep |  | X | X |  |  |  |
|  | *Gomesa foliosa* (Hook.) Klotzsch & Rchb.f. | Ep |  | X | X |  | X |  |
|  | *Gomesa glaziovii* Cogn. | Ep |  | X | X |  |  |  |
|  | *Gomesa gomezoides* (Barb.Rodr.) Pabst | Ep |  | X | X |  |  |  |
|  | *Gomesa handroi* (Hoehne) Pabst | Ep |  | X | X |  |  |  |
|  | *Gomesa jucunda* (Rchb.f.) M.W.Chase & N.H.Williams | Ep |  | X | X |  |  |  |
|  | *Gomesa laxiflora* (Lindl.) Klotzsch & Rchb.f. | Ep |  | X | X |  |  |  |
|  | *Gomesa messmeriana* (Campacci) Laitano | Ep |  | X | X |  |  |  |
|  | *Gomesa paranaensis* Kraenzl. | Ep |  | X | X |  |  |  |
|  | *Gomesa petropolitana* (Pabst) M.W.Chase & N.H.Williams | Ep |  | X | X |  |  |  |
|  | *Gomesa planifolia* (Lindl.) Klotzsch & Rchb.f. | Ep |  |  | X |  |  |  |
|  | *Gomesa recurva* R.Br. | Ep |  |  | X |  | X |  |
|  | *Gomesa sessilis* Barb.Rodr. | Ep |  | X | X |  | X |  |
|  | *Gongora bufonia* Lindl. | Ep |  | X | X |  |  |  |
|  | *Gongora meneziana* V.P.Castro & G.Gerlach | Ep |  | X | X |  |  |  |
|  | *Gongora nigrita* Lindl. | Ep |  | X | X | X |  |  |
|  | *Gongora quinquenervis* Ruiz & Pav. | Ep |  |  | X | X |  |  |
|  | *Gongora vitorinoana* Chiron & L.C.Menezes | Ep |  | X | X |  |  |  |
|  | *Grandiphyllum auricula* (Vell.) Docha Neto | Ep |  | X | X |  |  |  |
|  | *Grandiphyllum divaricatum* (Lindl.) Docha Neto | Ep | VU | X | X |  |  |  |
|  | *Grandiphyllum edwallii* (Cogn.) Docha Neto | Ep |  | X | X |  |  |  |
|  | *Grandiphyllum hians* (Lindl.) Docha Neto | Ep | VU | X | X |  |  |  |
|  | *Grandiphyllum pohlianum* (Cogn.) Docha Neto | Ep |  | X | X |  |  |  |
|  | *Grandiphyllum pulvinatum* (Lindl.) Docha Neto | Ep |  | X | X |  |  |  |
|  | *Grandiphyllum schunkianum* (Campacci & Catharino) Docha Neto | Ep |  | X | X |  |  |  |
|  | *Grandiphyllum sphegiferum* (Lindl.) Docha Neto | Ep |  | X | X |  |  |  |
|  | *Grobya amherstiae* Lindl. | Ep |  | X | X |  | X |  |
|  | *Grobya fascifera* Rchb.f. | Ep | VU | X | X |  |  |  |
|  | *Grobya galeata* Lindl. | Ep |  | X | X |  |  |  |
|  | *Grobya guieselii* F.Barros & R.Lourenço | Ep |  | X | X |  |  |  |
|  | *Hadrolaelia alaorii* (Brieger & Bicalho) Chiron & V.P.Castro | Ep | CR | X | X |  |  |  |
|  | *Hadrolaelia coccinea* (Lindl.) Chiron & V.P.Castro | Ep |  |  | X |  |  |  |
|  | *Hadrolaelia dayana* (Rchb.f.) Chiron & V.P.Castro | Ep |  | X | X |  |  |  |
|  | *Hadrolaelia mantiqueirae* (Fowlie) Fowlie | Ep |  | X | X |  |  |  |
|  | *Hadrolaelia pumila* (Hook.) Chiron & V.P.Castro | Ep | VU | X | X |  |  |  |
|  | *Hadrolaelia pygmaea* (Pabst) Chiron & V.P.Castro | Ep | EN | X | X |  |  |  |
|  | *Hadrolaelia wittigiana* (Barb.Rodr.) Chiron & V.P.Castro | Ep | EN | X | X |  |  |  |
|  | *Heterotaxis brasiliensis* (Brieger & Bicalho) F.Barros | Ep |  | X | X |  |  |  |
|  | *Heterotaxis discolor* (Lodd. ex Lindl.) Ojeda & Carnevali | Ep |  |  | X | X |  |  |
|  | *Heterotaxis valenzuelana* (A.Rich.) F.Barros | Ep |  |  | X |  |  |  |
|  | *Hoehneella gehrtiana* (Hoehne) Ruschi | Ep |  | X | X |  |  |  |
|  | *Hoehneella heloisae* Ruschi | Ep | CR | X | X |  |  |  |
|  | *Homalopetalum hypoleptum* (Lindl.) Soto-Arenas | Ep |  | X | X |  |  |  |
|  | *Houlletia broklehurstiana* Lindl. | Ep | EN | X | X |  |  |  |
|  | *Huntleya meleagris* Lindl. | Ep |  |  | X |  |  |  |
|  | *Ionopsis utricularioides* (Sw.) Lindl. | Ep |  |  | X | X | X |  |
|  | *Isabelia pulchella* (Kraenzl.) Senghas & Teuscher | Ep |  | X | X |  |  |  |
|  | *Isabelia violacea* (Lindl.) van den Berg & M.W.Chase | Ep |  | X | X |  | X |  |
|  | *Isabelia virginalis* Barb.Rodr. | Ep | VU |  | X |  |  |  |
|  | *Isochilus linearis* (Jacq.) R.Br. | Ep |  |  | X |  | X |  |
|  | *Jacquiniella globosa* (Jacq.) Schltr. | Ep |  |  | X | X |  |  |
|  | *Jacquiniella teretifolia* (Sw.) Britton & Wilson | Ep |  |  | X | X |  |  |
|  | *Koellensteinia graminea* (Lindl.) Rchb.f. | Ep |  | X | X |  |  |  |
|  | *Laelia gloriosa* (Rchb.f.) L.O.Williams | Ep |  |  | X | X | X |  |
|  | *Lankesterella caespitosa* (Lindl.) Hoehne | Ep |  | X | X |  |  |  |
|  | *Lankesterella ceracifolia* (Barb.Rodr.) Mansf. | Ep |  | X | X |  |  | X |
|  | *Lankesterella gnoma* (Kraenzl.) Hoehne | Ep |  | X | X |  |  |  |
|  | *Lankesterella longicollis* (Cogn.) Hoehne | Ep |  | X | X |  |  |  |
|  | *Lankesterella majus* (Hoehne & Schltr.) Mansf. | Ep |  | X | X |  |  |  |
|  | *Lankesterella parvula* (Kraenzl.) Pabst | Ep |  | X | X |  |  |  |
|  | *Lankesterella pilosa* (Cogn.) Hoehne | Ep |  | X | X |  |  |  |
|  | *Lankesterella spannageliana* (Hoehne & Brade) Mansf. | Ep |  | X | X |  |  |  |
|  | *Lepanthopsis densiflora* (Barb.Rodr.) Ames | Ep |  | X | X |  |  |  |
|  | *Lepanthopsis floripecten* (Rchb.f.) Ames | Ep |  |  | X | X |  |  |
|  | *Leptotes bicolor* Lindl. | Ep |  |  | X |  | X |  |
|  | *Leptotes bohnkiana* Campacci | Ep |  | X | X |  |  |  |
|  | *Leptotes harryphillipsii* Christenson | Ep |  | X | X |  |  |  |
|  | *Leptotes pauloensis* Hoehne | Ep |  | X | X |  |  |  |
|  | *Leptotes pohlitinocoi* V.P.Castro & Chiron | Ep |  | X | X |  |  |  |
|  | *Leptotes tenuis* Rchb.f. | Ep |  | X | X |  |  |  |
|  | *Leptotes unicolor* Barb.Rodr. | Ep |  |  | X |  |  |  |
|  | *Lockhartia ludibunda* Rchb.f. | Ep |  | X | X | X |  |  |
|  | *Lockhartia lunifera* (Lindl.) Rchb.f. | Ep |  | X | X | X |  |  |
|  | *Loefgrenianthus blanche-amesii* (Loefgr.) Hoehne | Ep |  | X | X |  |  |  |
|  | *Lophiaris pumila* (Lindl.) Braem | Ep |  |  | X |  | X |  |
|  | *Lophiaris schwambachiae* (V.P.Castro & Toscano) Braem | Ep | VU | X | X | X |  |  |
|  | *Macradenia multiflora* (Kraenzl.) Cogn. | Ep |  | X | X | X | X |  |
|  | *Macradenia paulensis* Cogn. | Ep |  | X | X |  |  |  |
|  | *Mapinguari desvauxianus* (Rchb.f.) Carnevali & R.Singer | Ep |  |  | X | X |  |  |
|  | *Masdevallia curtipes* Barb.Rodr. | Ep |  | X | X |  |  |  |
|  | *Masdevallia discoidea* Luer & Würstle | Ep | CR | X | X |  |  |  |
|  | *Masdevallia infracta* Lindl. | Ep |  | X | X |  | X |  |
|  | *Masdevallia sururuana* Campacci | Ep |  | X | X |  |  |  |
|  | *Maxillaria binotii* De Wild. | Ep |  | X | X |  |  |  |
|  | *Maxillaria bradei* Schltr. | Ep |  | X | X |  |  |  |
|  | *Maxillaria candida* Lodd. ex Lindl. | Ep |  | X | X |  |  |  |
|  | *Maxillaria caparaoensis* Brade | Ep |  | X | X |  | X |  |
|  | *Maxillaria chlorantha* Lindl. | Ep |  |  | X | X |  |  |
|  | *Maxillaria leucaimata* Barb.Rodr. | Ep |  | X | X | X |  |  |
|  | *Maxillaria lindleyana* Schltr. | Ep |  | X | X |  |  |  |
|  | *Maxillaria milenae* V.P.Castro & Chiron | Ep |  | X | X |  |  |  |
|  | *Maxillaria monantha* Barb.Rodr. | Ep |  | X | X |  |  |  |
|  | *Maxillaria ochroleuca* Lodd. ex Lindl. | Ep |  |  | X | X | X |  |
|  | *Maxillaria porrecta* Lindl. | Ep |  |  | X | X | X |  |
|  | *Maxillaria pterocarpa* Barb.Rodr. | Ep |  | X | X |  |  |  |
|  | *Maxillaria robusta* Barb.Rodr. | Ep |  | X | X | X |  |  |
|  | *Maxillaria rodriguesii* Cogn. | Ep |  | X | X |  |  |  |
|  | *Maxillaria silvana* Campacci | Ep |  | X | X |  |  |  |
|  | *Maxillaria spiritusanctensis* Pabst | Ep |  | X | X |  |  |  |
|  | *Maxillariella robusta* (Barb.Rodr.) M.A.Blanco & Carnevali | Ep |  | X | X |  |  |  |
|  | *Microlaelia lundii* (Rchb.f.) Chiron & V.P.Castro | Ep |  | X | X |  | X |  |
|  | *Miltonia candida* Lindl. | Ep |  | X | X |  |  |  |
|  | *Miltonia clowesii* Lindl. | Ep |  | X | X |  |  |  |
|  | *Miltonia cuneata* Lindl. | Ep |  | X | X |  |  |  |
|  | *Miltonia flava* Lindl. | Ep |  | X | X |  |  |  |
|  | *Miltonia flavescens* (Lindl.) Lindl. | Ep |  |  | X |  |  | X |
|  | *Miltonia kayasimae* Pabst | Ep | CR | X | X |  |  |  |
|  | *Miltonia moreliana* A.Rich. | Ep |  | X | X |  |  |  |
|  | *Miltonia regnellii* Rchb.f. | Ep |  | X | X |  |  |  |
|  | *Miltonia russelliana* (Lindl.) Lindl. | Ep |  | X | X |  |  |  |
|  | *Miltonia spectabilis* Lindl. | Ep |  |  | X |  |  |  |
|  | *Mormolyca calimaniana* (V.P.Castro) F.Barros & L.Guimarães | Ep |  | X | X |  |  |  |
|  | *Mormolyca cleistogama* (Brieger & Illg) M.A.Blanco | Ep |  | X | X | X |  |  |
|  | *Mormolyca galeata* (C.Schweinf.) Garay & Wirth | Ep |  | X | X |  |  |  |
|  | *Mormolyca rufescens* (Lindl.) M.A.Blanco | Ep |  |  | X | X |  |  |
|  | *Myoxanthus exasperatus* (Lindl.) Luer | Ep |  |  | X |  |  |  |
|  | *Myoxanthus lonchophyllus* (Barb.Rodr.) Luer | Ep |  | X | X |  | X |  |
|  | *Myoxanthus pulvinatus* (Barb.Rodr.) Luer | Ep |  | X | X |  |  |  |
|  | *Myoxanthus punctatus* (Barb.Rodr.) Luer | Ep |  | X | X |  |  |  |
|  | *Myoxanthus ruschii* Fraga & L.Kollmann | Ep | CR | X | X |  |  |  |
|  | *Myoxanthus seidelii* Pabst | Ep | CR | X | X |  |  |  |
|  | *Nemaconia striata* (Lindl.) van den Berg *et al.* | Ep |  |  | X |  |  |  |
|  | *Neogardneria murrayana* (Gardner ex Hook.) Schltr. | Ep | EN | X | X |  |  |  |
|  | *Nitidobulbon nasutum* (Rchb.f.) Ojeda & Carnevali | Ep |  |  | X | X |  |  |
|  | *Notylia barkeri* Lindl. | Ep |  |  | X | X |  |  |
|  | *Notylia hemitricha* Barb.Rodr. | Ep |  | X | X |  | X |  |
|  | *Notylia inversa* Barb.Rodr. | Ep |  | X | X |  |  |  |
|  | *Notylia longispicata* Hoehne & Schltr. | Ep |  | X | X |  |  |  |
|  | *Notylia lyrata* S.Moore | Ep |  |  | X | X | X |  |
|  | *Notylia microchila* Cogn. | Ep | EN |  | X | X |  |  |
|  | *Notylia nemorosa* Barb.Rodr. | Ep |  | X | X |  |  |  |
|  | *Notylia pubescens* Lindl. | Ep |  | X | X |  |  |  |
|  | *Notylia stenatha* Rchb.f. | Ep |  | X | X |  | X |  |
|  | *Notylia trullulifera* Brade | Ep |  | X | X |  | X |  |
|  | *Octomeria aetheoantha* Barb.Rodr. | Ep |  | X | X |  |  |  |
|  | *Octomeria alexandri* Schltr. | Ep | EN | X | X |  | X |  |
|  | *Octomeria aloefolia* Barb.Rodr. | Ep |  | X | X |  |  |  |
|  | *Octomeria anceps* Porto & Brade | Ep |  | X | X |  |  |  |
|  | *Octomeria caetensis* Pabst | Ep |  | X | X |  |  |  |
|  | *Octomeria caldensis* Hoehne | Ep |  | X | X |  |  |  |
|  | *Octomeria campos-portoi* Schltr. | Ep |  | X | X |  |  |  |
|  | *Octomeria cariocana* Pabst | Ep |  | X | X |  |  |  |
|  | *Octomeria chamaeleptotes* Rchb.f. | Ep | VU |  | X |  |  |  |
|  | *Octomeria chloidophylla* (Rchb.f.) Garay | Ep |  | X | X |  |  |  |
|  | *Octomeria cochlearis* Rchb.f. | Ep |  | X | X |  |  |  |
|  | *Octomeria concolor* Barb.Rodr. | Ep |  |  | X |  |  |  |
|  | *Octomeria crassifolia* Lindl. | Ep |  |  | X |  | X |  |
|  | *Octomeria cucullata* Porto & Brade | Ep |  | X | X |  |  |  |
|  | *Octomeria decumbens* Cogn. | Ep |  | X | X |  |  |  |
|  | *Octomeria diaphana* Lindl. | Ep |  | X | X |  |  |  |
|  | *Octomeria estrellensis* Hoehne | Ep |  | X | X |  |  |  |
|  | *Octomeria exchlorophyllata* Barb.Rodr. | Ep |  | X | X |  |  |  |
|  | *Octomeria exigua* C.Schweinf. | Ep |  | X | X |  |  |  |
|  | *Octomeria fibrifera* Schltr. | Ep |  | X | X |  |  |  |
|  | *Octomeria fimbriata* Porto & Peixoto | Ep |  | X | X |  |  |  |
|  | *Octomeria flabellifera* Pabst | Ep |  | X | X |  |  |  |
|  | *Octomeria geraensis* Barb.Rodr. | Ep | VU | X | X |  |  |  |
|  | *Octomeria gracilis* Lodd. ex Lindl. | Ep |  | X | X |  |  |  |
|  | *Octomeria grandiflora* Lindl. | Ep |  |  | X | X | X |  |
|  | *Octomeria hatschbachii* Schltr. | Ep | VU | X | X |  |  | X |
|  | *Octomeria helvola* Barb.Rodr. | Ep |  | X | X |  |  |  |
|  | *Octomeria hoehnei* Schltr. | Ep | EN | X | X |  |  |  |
|  | *Octomeria irrorata* Schltr. | Ep |  | X | X |  |  |  |
|  | *Octomeria itatiaiae* Brade & Pabst | Ep |  | X | X |  |  |  |
|  | *Octomeria juncifolia* Barb.Rodr. | Ep |  | X | X |  |  |  |
|  | *Octomeria leptophylla* Barb.Rodr. | Ep |  | X | X |  |  |  |
|  | *Octomeria lichenicola* Barb.Rodr. | Ep | EN | X | X |  |  |  |
|  | *Octomeria linearifolia* Barb.Rodr. | Ep |  | X | X |  |  |  |
|  | *Octomeria lithophila* Barb.Rodr. | Ep |  | X | X |  |  |  |
|  | *Octomeria micrantha* Barb.Rodr. | Ep |  |  | X |  |  |  |
|  | *Octomeria minuta* Cogn. | Ep |  | X | X |  |  |  |
|  | *Octomeria montana* Barb.Rodr. | Ep |  | X | X |  |  |  |
|  | *Octomeria multiflora* Barb.Rodr. | Ep |  | X | X |  |  |  |
|  | *Octomeria ochroleuca* Barb.Rodr. | Ep |  | X | X |  |  |  |
|  | *Octomeria octomeriantha* (Hoehne) Pabst | Ep |  | X | X |  |  |  |
|  | *Octomeria palmyrabellae* Barb.Rodr. | Ep |  | X | X |  |  |  |
|  | *Octomeria praestans* Barb.Rodr. | Ep |  | X | X |  |  |  |
|  | *Octomeria pusilla* Lindl. | Ep |  |  | X |  |  |  |
|  | *Octomeria recchiana* Hoehne | Ep |  | X | X |  |  |  |
|  | *Octomeria rhodoglossa* Schltr. | Ep |  | X | X |  |  |  |
|  | *Octomeria riograndensis* Schltr. | Ep |  | X | X |  |  |  |
|  | *Octomeria rodeiensis* Barb.Rodr. | Ep |  | X | X |  |  |  |
|  | *Octomeria rodriguesii* Cogn. | Ep |  | X | X |  |  |  |
|  | *Octomeria rohrii* Pabst | Ep |  | X | X |  |  |  |
|  | *Octomeria rotundiglossa* Hoehne | Ep |  | X | X |  |  |  |
|  | *Octomeria rubrifolia* Barb.Rodr. | Ep |  | X | X |  |  |  |
|  | *Octomeria sagittata* (Rchb.f.) Garay | Ep |  |  | X | X |  |  |
|  | *Octomeria sancti-angeli* Kraenzl. | Ep |  | X | X |  |  |  |
|  | *Octomeria sarcophylla* Barb.Rodr. | Ep |  | X | X |  |  |  |
|  | *Octomeria spannagelii* Hoehne | Ep |  | X | X |  |  |  |
|  | *Octomeria stellaris* Barb.Rodr. | Ep |  | X | X |  |  | X |
|  | *Octomeria tricolor* Rchb.f. | Ep |  | X | X |  |  |  |
|  | *Octomeria truncicola* Barb.Rodr. | Ep | VU | X | X |  |  |  |
|  | *Octomeria unguiculata* Schltr. | Ep |  | X | X |  |  |  |
|  | *Octomeria warmingii* Rchb.f. | Ep |  |  | X |  | X |  |
|  | *Octomeria wawrae* Rchb.f. | Ep | EN | X | X |  |  |  |
|  | *Octomeria wilsoniana* Hoehne | Ep | CR | X | X |  |  |  |
|  | *Oncidium baueri* Lindl. | Ep |  |  | X | X |  |  |
|  | *Ornithidium pendulum* (Poepp. & Endl.) Cogn. | Ep |  |  | X |  |  |  |
|  | *Ornithidium rigidum* (Barb.Rodr.) M.A.Blanco & Ojeda | Ep |  |  | X |  |  |  |
|  | *Ornithocephalus brachystachyus* Schltr. | Ep |  | X | X |  |  |  |
|  | *Ornithocephalus myrticola* Lindl. | Ep |  |  | X |  | X |  |
|  | *Ornithophora itapetingensis* (V.P.Castro & Chiron) F.Barros & V.T.Rodrigues | Ep |  | X | X |  |  |  |
|  | *Ornithophora radicans* (Rchb.f.) Garay & Pabst | Ep |  | X | X | X |  |  |
|  | *Pabstia jugosa* (Lindl.) Garay | Ep | EN | X | X |  |  |  |
|  | *Pabstia modestior* (Rchb.f.) Garay | Ep |  | X | X |  |  |  |
|  | *Pabstia placanthera* (Hook.) Garay | Ep |  | X | X |  |  |  |
|  | *Pabstia schunkiana* V.P.Castro | Ep | CR | X | X |  |  |  |
|  | *Pabstia viridis* (Lindl.) Garay | Ep |  | X | X |  |  |  |
|  | *Pabstiella alligatorifera* (Rchb.f.) Luer | Ep |  | X | X |  |  |  |
|  | *Pabstiella arcuata* (Lindl.) Luer | Ep |  | X | X |  |  |  |
|  | *Pabstiella armeniaca* (Barb.Rodr.) L. Kollmann | Ep |  | X | X |  |  |  |
|  | *Pabstiella aveniformis* (Hoehne) Luer | Ep |  |  | X |  |  |  |
|  | *Pabstiella bacillaris* (Pabst) Luer | Ep | EN | X | X |  |  |  |
|  | *Pabstiella bradei* (Schltr.) Luer | Ep |  | X | X |  |  |  |
|  | *Pabstiella calcarata* (Cogn.) Luer | Ep |  | X | X |  |  |  |
|  | *Pabstiella campestris* (Barb.Rodr.) Luer | Ep |  | X | X |  |  |  |
|  | *Pabstiella carinifera* (Barb.Rodr.) Luer | Ep | VU | X | X |  |  |  |
|  | *Pabstiella carrisii* (Brade) Luer | Ep |  | X | X |  |  |  |
|  | *Pabstiella castellensis* (Brade) Luer | Ep | CR | X | X |  |  |  |
|  | *Pabstiella colorata* (Pabst) F.Barros & C.F.Hall | Ep |  | X | X |  |  |  |
|  | *Pabstiella conspersa* (Hoehne) Luer | Ep | EN | X | X |  |  |  |
|  | *Pabstiella cordilabia* (Pabst) Luer | Ep |  | X | X |  |  |  |
|  | *Pabstiella crassicaulis* (Barb.Rodr.) Luer | Ep |  | X | X |  |  |  |
|  | *Pabstiella curti-bradei* (Pabst) Luer | Ep |  | X | X |  |  |  |
|  | *Pabstiella elegantula* (Cogn.) Luer | Ep |  | X | X |  |  |  |
|  | *Pabstiella ephemera* (Lindl.) Luer | Ep |  | X | X | X |  |  |
|  | *Pabstiella fasciata* (Seehawer) Luer | Ep |  | X | X |  |  |  |
|  | *Pabstiella fluminensis* (Pabst) Luer | Ep |  | X | X |  |  |  |
|  | *Pabstiella fusca* (Lindl.) Chiron & Xim.Bols. | Ep |  |  | X |  | X |  |
|  | *Pabstiella garayi* (Pabst) Luer | Ep | CR | X | X |  |  |  |
|  | *Pabstiella ghillanyi* (Pabst) Luer | Ep |  | X | X |  |  |  |
|  | *Pabstiella gracilicaulis* (Seehawer) Luer | Ep |  | X | X |  |  |  |
|  | *Pabstiella granulosa* (Barb.Rodr.) Chiron & Xim.Bols. | Ep |  | X | X |  |  |  |
|  | *Pabstiella henrique-aragonii* (Pabst) Chiron & Xim.Bols. | Ep |  | X | X |  |  |  |
|  | *Pabstiella hians* (Lindl.) Luer | Ep |  | X | X |  |  |  |
|  | *Pabstiella hypnicola* (Lindl.) Luer | Ep |  |  | X |  | X |  |
|  | *Pabstiella imbeana* (Brade) F.Barros & C.F.Hall | Ep |  | X | X |  |  |  |
|  | *Pabstiella leucosepala* (Loefgr.) F.Barros & C.F.Hall | Ep |  | X | X |  |  |  |
|  | *Pabstiella lineolata* (Barb.Rodr.) Luer | Ep |  | X | X |  |  |  |
|  | *Pabstiella lingua* (Lindl.) Luer | Ep | EN | X | X |  |  |  |
|  | *Pabstiella matinhensis* (Hoehne) Luer | Ep |  | X | X |  |  |  |
|  | *Pabstiella mentigera* (Kraenzl.) L.Kollmann | Ep |  | X | X |  |  |  |
|  | *Pabstiella miniatolineolata* (Hoehne) Luer | Ep |  | X | X |  |  |  |
|  | *Pabstiella mirabilis* (Schltr.) Brieger & Senghas | Ep |  | X | X |  |  |  |
|  | *Pabstiella miragliae* (J.E.Leite) Luer | Ep |  | X | X |  |  |  |
|  | *Pabstiella mouraeoides* (Hoehne) Luer | Ep |  | X | X |  |  |  |
|  | *Pabstiella pandurifera* (Lindl.) F.Barros & C.F.Hall | Ep |  | X | X |  |  |  |
|  | *Pabstiella parvifolia* (Lindl.) Luer | Ep |  | X | X |  |  |  |
|  | *Pabstiella pellifeloidis* (Barb.Rodr.) Luer | Ep |  | X | X |  |  |  |
|  | *Pabstiella piraquarensis* (Hoehne) Luer | Ep |  | X | X |  |  |  |
|  | *Pabstiella pleurothalloides* (Cogn.) Luer | Ep |  | X | X |  |  |  |
|  | *Pabstiella podoglossa* (Hoehne) Luer | Ep |  | X | X |  |  |  |
|  | *Pabstiella pristeoglossa* (Rchb.f. & Warm.) Luer | Ep |  | X | X |  | X |  |
|  | *Pabstiella pterophora* (Cogn.) Chiron | Ep |  | X | X |  |  |  |
|  | *Pabstiella punctatifolia* (Barb.Rodr.) Luer | Ep |  | X | X |  |  |  |
|  | *Pabstiella quadridentata* (Barb.Rodr.) Luer | Ep |  | X | X |  |  |  |
|  | *Pabstiella ramphastorhyncha* (Barb.Rodr.) L.Kollmann | Ep |  | X | X |  | X |  |
|  | *Pabstiella robertoi* (Luer & Toscano) Luer | Ep |  | X | X |  |  |  |
|  | *Pabstiella rubrolineata* (Hoehne) Luer | Ep |  | X | X |  |  |  |
|  | *Pabstiella ruschii* (Hoehne) Luer | Ep | CR | X | X |  |  |  |
|  | *Pabstiella sarcopetala* (Barb.Rodr.) Luer | Ep |  | X | X |  |  |  |
|  | *Pabstiella seriata* (Lindl.) F.Barros & C.F.Hall | Ep |  |  | X |  |  |  |
|  | *Pabstiella sordida* (Kraenzl.) Luer | Ep |  | X | X |  | X |  |
|  | *Pabstiella spathuliglossa* (Hoehne) Luer | Ep |  | X | X |  |  |  |
|  | *Pabstiella tabacina* (Barb.Rodr.) Luer | Ep |  | X | X |  |  |  |
|  | *Pabstiella tenera* (Barb.Rodr.) Luer | Ep |  | X | X |  |  |  |
|  | *Pabstiella transparens* (Schltr.) Luer | Ep |  | X | X |  |  |  |
|  | *Pabstiella trifida* (Lindl.) Luer | Ep |  | X | X |  |  |  |
|  | *Pabstiella trimeropetala* (Pabst) Luer | Ep |  | X | X |  |  |  |
|  | *Pabstiella tripterantha* (Rchb.f.) F.Barros | Ep |  |  | X |  |  |  |
|  | *Pabstiella truncicola* (Rchb.f.) Luer | Ep |  | X | X |  |  |  |
|  | *Pabstiella uniflora* (Lindl.) Luer | Ep |  | X | X | X |  |  |
|  | *Pabstiella vellozoana* (Schltr.) Luer | Ep |  | X | X |  |  |  |
|  | *Pabstiella versicolor* (Porsch) Luer | Ep |  | X | X |  |  |  |
|  | *Pabstiella viridula* (Barb.Rodr.) F.Barros & C.F.Hall | Ep |  | X | X |  |  |  |
|  | *Pabstiella wacketii* (Handro & Pabst) Luer | Ep |  | X | X |  |  |  |
|  | *Pabstiella wanderbildtiana* (Pabst) F.Barros & C.F.Hall | Ep |  | X | X |  |  |  |
|  | *Pabstiella wawraeana* (Barb.Rodr.) Chiron & Xim.Bols. | Ep |  | X | X |  |  |  |
|  | *Phloeophila bradei* (Schltr.) Garay | Ep |  | X | X |  |  |  |
|  | *Phloeophila nummularia* (Barb.Rodr.) Hoehne & Schltr. | Ep |  |  | X |  |  |  |
|  | *Phloeophila similis* (Schltr.) Garay | Ep |  |  | X |  |  |  |
|  | *Phymatidium aquinoi* Schltr. | Ep |  | X | X |  |  |  |
|  | *Phymatidium delicatulum* Lindl. | Ep |  | X | X |  |  |  |
|  | *Phymatidium falcifolium* Lindl. | Ep |  |  | X |  |  |  |
|  | *Phymatidium geiselii* Ruschi | Ep | EN | X | X |  |  |  |
|  | *Phymatidium glaziovii* Toscano | Ep | VU | X | X |  |  |  |
|  | *Phymatidium hysteranthum* Barb.Rodr. | Ep |  | X | X |  |  |  |
|  | *Phymatidium limae* Porto & Brade | Ep |  | X | X |  |  |  |
|  | *Phymatidium mellobarretoi* Hoehne & Williams | Ep |  | X | X |  |  |  |
|  | *Phymatidium microphyllum* (Barb.Rodr.) Toscano | Ep |  | X | X |  |  |  |
|  | *Phymatidium vogelii* Pabst | Ep | VU | X | X |  |  |  |
|  | *Phymatochilum brasiliense* Christenson | Ep |  |  | X |  |  |  |
|  | *Platyrhiza quadricolor* Barb.Rodr. | Ep |  | X | X |  |  |  |
|  | *Platystele oxyglossa* (Schltr.) Garay | Ep |  | X | X |  |  |  |
|  | *Pleurobotryum albopurpureum* (Kraenzl.) Garay | Ep |  | X | X |  |  |  |
|  | *Pleurobotryum atropurpureum* Barb.Rodr. | Ep |  | X | X |  | X |  |
|  | *Pleurobotryum crepinianum* (Cogn.) Hoehne | Ep |  | X | X |  |  |  |
|  | *Pleurobotryum hatschbachii* (Schltr.) Hoehne | Ep |  | X | X |  |  |  |
|  | *Pleurobotryum mantiquyranum* (Barb.Rodr.) Hoehne | Ep |  | X | X |  |  |  |
|  | *Pleurobotryum rhabdosepalum* (Schltr.) Hoehne | Ep |  | X | X |  |  |  |
|  | *Pleurobotryum subulifolium* (Kraenzl.) Pabst | Ep |  | X | X |  |  |  |
|  | *Pleurothallis ruscifolia* (Jacq.) R.Br. | Ep |  | X | X | X |  |  |
|  | *Pleurothallopsis nemorosa* (Barb.Rodr.) Porto & Brade | Ep |  | X | X |  |  |  |
|  | *Polycycnis silvana* F.Barros | Ep |  | X | X |  |  |  |
|  | *Polystachya bradei* Schltr. | Ep |  | X | X |  |  |  |
|  | *Polystachya caespitosa* Barb.Rodr. | Ep |  | X | X |  |  |  |
|  | *Polystachya concreta* (Jacq.) Garay & Sweet | Ep |  |  | X | X | X | X |
|  | *Polystachya estrellensis* Rchb.f. | Ep |  | X | X | X | X | X |
|  | *Polystachya foliosa* (Lindl.) Rchb.f. | Ep |  |  | X | X | X |  |
|  | *Polystachya hoehneana* Schltrl. | Ep |  | X | X |  |  |  |
|  | *Polystachya micrantha* Schltr. | Ep |  | X | X |  | X | X |
|  | *Polystachya pinicola* Schltr. | Ep |  | X | X |  | X |  |
|  | *Prescottia lancifolia* Lindl. | Ep |  | X | X |  |  |  |
|  | *Promenaea acuminata* Schltr. | Ep |  | X | X |  |  |  |
|  | *Promenaea albescens* Schltr. | Ep |  | X | X |  |  |  |
|  | *Promenaea catharinensis* Schltr. | Ep |  | X | X |  |  |  |
|  | *Promenaea dusenii* Schltr. | Ep |  | X | X |  |  |  |
|  | *Promenaea fuerstenbergiana* Schltr. | Ep | EN | X | X |  |  |  |
|  | *Promenaea guttata* Rchb.f. | Ep |  | X | X |  |  |  |
|  | *Promenaea lentiginosa* Lindl. | Ep |  | X | X |  |  |  |
|  | *Promenaea nigricans* Königer & J.G.Weinm. | Ep |  | X | X |  |  |  |
|  | *Promenaea ovatiloba* (Kingle) Cogn. | Ep |  | X | X |  |  |  |
|  | *Promenaea paranaensis* Schltr. | Ep |  | X | X |  |  |  |
|  | *Promenaea riograndensis* Schltr. | Ep |  | X | X |  |  |  |
|  | *Promenaea rollisonii* (Lindl.) Lindl. | Ep |  | X | X |  |  |  |
|  | *Promenaea silvana* F.Barros & Catharino | Ep |  | X | X |  |  |  |
|  | *Promenaea stapelioides* (Link & Otto) Lindl. | Ep |  | X | X |  |  |  |
|  | *Promenaea xanthina* Lindl. | Ep |  | X | X |  |  |  |
|  | *Prosthechea alagoensis* (Pabst) W.E.Higgins | Ep |  | X | X |  |  |  |
|  | *Prosthechea allemanoides* (Hoehne) W.E.Higgins | Ep |  | X | X |  |  |  |
|  | *Prosthechea bueraremensis* (Campacci) Campacci | Ep |  | X | X |  |  |  |
|  | *Prosthechea bulbosa* (Vell.) W.E.Higgins | Ep |  |  | X |  |  | X |
|  | *Prosthechea calamaria* (Lindl.) W.E.Higgins | Ep |  | X | X |  |  |  |
|  | *Prosthechea campos-portoi* (Pabst) W.E.Higgins | Ep |  | X | X |  |  |  |
|  | *Prosthechea christyana* (Rchb.f.) Garay & Withner | Ep |  | X | X |  |  |  |
|  | *Prosthechea ebanii* Chiron & V.P.Castro | Ep |  | X | X |  |  |  |
|  | *Prosthechea fausta* (Rchb.f. ex Cogn.) W.E.Higgins | Ep |  | X | X |  |  |  |
|  | *Prosthechea fragans* (Sw.) W.E.Higgins | Ep |  |  | X | X |  |  |
|  | *Prosthechea glumacea* (Lindl.) W.E.Higgins | Ep |  |  | X |  |  |  |
|  | *Prosthechea kautskyi* (Pabst) W.E.Higgins | Ep |  | X | X |  |  |  |
|  | *Prosthechea pachysepala* (Klotzsch) Chiron & V.P.Castro | Ep |  | X | X |  | X |  |
|  | *Prosthechea papilio* (Vell.) W.E.Higgins | Ep |  | X | X |  |  |  |
|  | *Prosthechea punctifera* (Rchb.f.) W.E.Higgins | Ep |  | X | X |  |  |  |
|  | *Prosthechea pygmaea* (Hook.) W.E.Higgins | Ep |  |  | X | X |  |  |
|  | *Prosthechea regnelliana* (Hoehne & Schltr.) W.E.Higgins | Ep |  | X | X |  |  |  |
|  | *Prosthechea serpentilingua* Withner & D.G.Hunt | Ep |  | X | X |  |  |  |
|  | *Prosthechea sessiliflora* (Edwall) W.E.Higgins | Ep |  | X | X |  | X |  |
|  | *Prosthechea silvana* Catharino & V.P.Castro | Ep |  | X | X |  |  |  |
|  | *Prosthechea suzanensis* (Hoehne) W.E.Higgins | Ep |  | X | X |  |  |  |
|  | *Prosthechea vespa* (Vell.) W.E.Higgins | Ep |  | X | X | X | X |  |
|  | *Pseudolaelia aromatica* Campacci | Ep |  | X | X |  |  |  |
|  | *Pseudolaelia ataleiensis* Campacci | Ep |  | X | X |  |  |  |
|  | *Pseudolaelia corcovadensis* Porto & Brade | Ep |  | X | X |  |  |  |
|  | *Pseudolaelia dutrae* Ruschi | Ep | VU | X | X |  |  |  |
|  | *Pseudolaelia freyi* Chiron & V.P.Castro | Ep |  | X | X |  |  |  |
|  | *Pseudolaelia geraensis* Pabst | Ep |  | X | X |  | X |  |
|  | *Pseudolaelia irwiniana* Pabst | Ep |  | X | X |  | X |  |
|  | *Pseudolaelia vellozicola* (Hoehne) Porto & Brade | Ep |  | X | X |  | X |  |
|  | *Pygmaeorchis brasiliensis* Brade | Ep |  | X | X |  |  |  |
|  | *Rauhiella brasiliensis* Pabst & Braga | Ep |  | X | X |  |  |  |
|  | *Rauhiella seehaweri* (I.Bock) Toscano & Christenson | Ep |  | X | X |  |  |  |
|  | *Rauhiella silvana* Toscano | Ep | EN | X | X |  |  |  |
|  | *Rhetinantha notylioglossa* (Rchb.f.) M.A.Blanco | Ep |  |  | X | X |  |  |
|  | *Rodriguezia bahiensis* Rchb.f. | Ep |  | X | X |  |  |  |
|  | *Rodriguezia bifolia* Barb.Rodr. | Ep |  | X | X |  |  |  |
|  | *Rodriguezia bracteata* (Vell.) Hoehne | Ep |  | X | X |  |  |  |
|  | *Rodriguezia decora* (Lem.) Rchb.f. | Ep |  |  | X |  | X |  |
|  | *Rodriguezia leeana* Rchb.f. | Ep |  |  | X | X |  |  |
|  | *Rodriguezia leucantha* Barb.Rodr. | Ep |  | X | X |  |  |  |
|  | *Rodriguezia limae* Brade | Ep |  | X | X |  |  |  |
|  | *Rodriguezia obtusifolia* (Lindl.) Rchb.f. | Ep |  | X | X |  |  |  |
|  | *Rodriguezia pardina* Rchb.f. | Ep |  | X | X |  |  |  |
|  | *Rodriguezia pubescens* (Lindl.) Rchb.f. | Ep |  | X | X |  |  |  |
|  | *Rodriguezia rigida* (Lindl.) Rchb.f. | Ep |  | X | X |  |  |  |
|  | *Rodriguezia sticta* M.W.Chase | Ep |  | X | X |  |  |  |
|  | *Rodriguezia sucrei* Braga | Ep |  | X | X |  |  |  |
|  | *Rodriguezia venusta* Rchb.f. | Ep |  | X | X |  |  |  |
|  | *Rodrigueziopsis eleutherosepala* (Barb.Rodr.) Schltr. | Ep |  | X | X |  |  |  |
|  | *Rodrigueziopsis microphyta* (Barb.Rodr.) Schltr. | Ep |  | X | X |  |  |  |
|  | *Sanderella discolor* (Barb.Rodr.) Cogn. | Ep |  |  | X |  | X |  |
|  | *Sanderella riograndensis* Dutra ex Pabst | Ep |  |  | X |  |  |  |
|  | *Saundersia mirabilis* Rchb.f. | Ep | EN | X | X |  | X |  |
|  | *Saundersia paniculata* Brade | Ep | VU | X | X |  |  |  |
|  | *Scaphyglottis brasiliensis* (Schltr.) Dressler | Ep |  | X | X |  |  |  |
|  | *Scaphyglottis emarginata* (Garay) Dressler | Ep |  |  | X | X |  |  |
|  | *Scaphyglottis fusiformis* (Griseb.) Schult. | Ep |  |  | X | X |  |  |
|  | *Scaphyglottis livida* (Lindl.) Schltr. | Ep |  |  | X |  | X |  |
|  | *Scaphyglottis modesta* (Rchb.f.) Schltr. | Ep |  |  | X | X |  | X |
|  | *Scaphyglottis prolifera* (R.Br.) Cogn. | Ep |  |  | X | X | X |  |
|  | *Scaphyglottis reflexa* Lindl. | Ep |  |  | X | X |  |  |
|  | *Scaphyglottis sickii* Pabst | Ep |  |  | X | X |  |  |
|  | *Schunkea vierlingii* Senghas | Ep |  | X | X |  |  |  |
|  | *Scuticaria hadwenii* Hook. | Ep |  | X | X |  |  |  |
|  | *Scuticaria kautskyi* Pabst | Ep | CR | X | X |  |  |  |
|  | *Scuticaria novaesii* F.Barros & Catharino | Ep |  | X | X |  |  |  |
|  | *Scuticaria strictifolia* Hoehne | Ep | EN | X | X |  |  |  |
|  | *Sobralia sesselis* Lindl. | Ep |  | X | X | X | X | X |
|  | *Sophronitis alagoensis* V.P.Castro & Chiron | Ep |  | X | X |  |  |  |
|  | *Sophronitis cernua* Lindl. | Ep |  |  | X |  | X |  |
|  | *Sophronitis pterocarpa* Lindl. & Paxton | Ep |  |  | X |  |  |  |
|  | *Specklinia acutidentata* (Cogn.) Luer | Ep |  | X | X |  |  |  |
|  | *Specklinia barbosana* (De Wild.) Campacci | Ep |  | X | X |  |  |  |
|  | *Specklinia deltoglossa* (Cogn.) Luer | Ep |  | X | X |  |  |  |
|  | *Specklinia gomesferreirae* (Pabst) Luer | Ep | CR | X | X |  |  |  |
|  | *Specklinia grobyi* (Batem. ex Lindl.) F.Barros | Ep |  |  | X | X |  | X |
|  | *Specklinia hymenantha* (Lindl.) F. Barros & V.T.Rodrigues | Ep |  | X | X |  |  |  |
|  | *Specklinia marginalis* (Rchb.f.) F.Barros | Ep |  |  | X |  |  |  |
|  | *Specklinia pantherina* (Seehawer) Luer | Ep |  | X | X |  |  |  |
|  | *Specklinia scabripes* (Lindl.) Luer | Ep |  | X | X |  |  |  |
|  | *Specklinia subpicta* (Schltr.) F.Barros | Ep |  | X | X |  |  |  |
|  | *Stanhopea guttulata* Lindl. | Ep |  | X | X |  |  |  |
|  | *Stanhopea insignis* Frost ex Hook. | Ep |  | X | X |  |  |  |
|  | *Stanhopea lietzei* (Regel) Schltr. | Ep |  | X | X |  |  |  |
|  | *Stelis amoena* Pridgeon & M.W. Chase | Ep |  | X | X |  |  |  |
|  | *Stelis aprica* Lindl. | Ep |  | X | X |  | X | X |
|  | *Stelis aquinoana* Schltr. | Ep |  | X | X |  |  |  |
|  | *Stelis argentata* Lindl. | Ep |  | X | X | X |  |  |
|  | *Stelis caespitosa* Lindl. | Ep |  | X | X |  |  |  |
|  | *Stelis catharinensis* Lindl. | Ep |  | X | X | X |  |  |
|  | *Stelis chlorantha* Barb.Rodr. | Ep |  | X | X |  |  |  |
|  | *Stelis deregularis* Barb.Rodr. | Ep |  | X | X |  |  |  |
|  | *Stelis fraterna* Lindl. | Ep |  |  | X |  |  |  |
|  | *Stelis gelida* (Lindl.) Pridgeon & M.W.Chase | Ep |  |  | X | X |  |  |
|  | *Stelis gigas* Barb.Rodr. | Ep |  | X | X |  |  |  |
|  | *Stelis grandiflora* Lindl. | Ep |  |  | X |  |  |  |
|  | *Stelis intermedia* Poepp. & Endl. | Ep |  |  | X |  |  |  |
|  | *Stelis itatiayae* Schltr. | Ep |  | X | X |  |  |  |
|  | *Stelis laxiflora* (Porsch) Pridgeon & M.W.Chase | Ep |  | X | X |  |  |  |
|  | *Stelis leiningii* Pabst | Ep |  | X | X |  |  |  |
|  | *Stelis loefgrenii* Cogn. | Ep |  | X | X |  |  |  |
|  | *Stelis megantha* Barb.Rodr. | Ep |  | X | X |  |  |  |
|  | *Stelis minutiflora* (Hoffmanns.) Rchb.f. ex Hoffmanns. | Ep |  | X | X |  |  |  |
|  | *Stelis modesta* Barb.Rodr. | Ep |  | X | X |  |  |  |
|  | *Stelis oligantha* Barb.Rodr. | Ep |  | X | X |  |  |  |
|  | *Stelis palmeirensis* Barb.Rodr. | Ep |  | X | X |  |  |  |
|  | *Stelis papaquerensis* Rchb.f. | Ep |  |  | X | X |  |  |
|  | *Stelis parvifolia* Garay | Ep |  | X | X |  |  |  |
|  | *Stelis parvula* Lindl. | Ep |  | X | X |  | X | X |
|  | *Stelis pauciflora* Lindl. | Ep |  | X | X | X |  |  |
|  | *Stelis pauloensis* Hoehne & Schltr. | Ep |  | X | X |  |  |  |
|  | *Stelis peliochyla* Barb.Rodr. | Ep |  | X | X |  |  |  |
|  | *Stelis perpusilla* Cogn. | Ep |  | X | X |  | X |  |
|  | *Stelis porschiana* Schltr. | Ep |  | X | X |  |  |  |
|  | *Stelis pumila* Pridgeon & M.W. Chase | Ep |  | X | X |  |  |  |
|  | *Stelis pusilla* Kunth | Ep |  |  | X |  |  |  |
|  | *Stelis reitzii* Garay | Ep |  | X | X |  |  |  |
|  | *Stelis ruprechtiana* Rchb.f. | Ep |  | X | X |  |  |  |
|  | *Stelis schenkii* Schltr. | Ep |  | X | X |  |  |  |
|  | *Stelis serrulata* (Barb.Rodr.) Pridgeon & M.W. Chase | Ep |  | X | X |  |  |  |
|  | *Stelis susanensis* (Hoehne) Pridgeon & M.W.Chase | Ep |  | X | X |  |  |  |
|  | *Stelis synsepala* Cogn. | Ep |  | X | X |  |  |  |
|  | *Stelis thermophila* Schltr. | Ep |  | X | X |  |  |  |
|  | *Stelis triangularis* Barb.Rodr. | Ep |  | X | X |  |  |  |
|  | *Stenia bohnkiana* V.P.Castro & G.F.Carr | Ep |  | X | X |  |  |  |
|  | *Stigmatosema polyaden* (Vell.) Garay | Ep |  |  | X |  | X |  |
|  | *Thysanoglossa jordanensis* Porto & Brade | Ep | EN | X | X |  |  |  |
|  | *Thysanoglossa organensis* Brade | Ep |  | X | X |  |  |  |
|  | *Thysanoglossa spiritusanctensis* N. Sanson & Chiron | Ep |  | X | X |  |  |  |
|  | *Trichocentrum fuscum* Lindl. | Ep |  |  | X | X | X |  |
|  | *Trichocentrum tenuiflorum* Lindl. | Ep |  | X | X |  |  |  |
|  | *Trichopilia brasiliensis* Cogn. | Ep |  | X | X | X | X |  |
|  | *Trichopilia laxa* (Lindl.) Rchb.f. | Ep |  |  | X |  |  |  |
|  | *Trichopilia santoslimae* Brade | Ep | CR | X | X |  |  |  |
|  | *Trichosalpinx dura* (Lindl.) Luer | Ep |  |  | X | X |  |  |
|  | *Trichosalpinx mathildae* (Brade) Toscano & Luer | Ep |  | X | X |  |  |  |
|  | *Trichosalpinx montana* (Barb.Rodr.) Luer | Ep |  | X | X |  |  |  |
|  | *Trichosalpinx purpurea* Seehawer | Ep |  | X | X |  |  |  |
|  | *Trichosalpinx violacea* (Lem.) Luer | Ep |  | X | X |  |  |  |
|  | *Trigonidium acuminatum* Batem. ex Lindl. | Ep |  |  | X | X | X |  |
|  | *Trigonidium latifolium* Lindl. | Ep |  | X | X |  | X |  |
|  | *Trigonidium macranthum* Barb.Rodr. | Ep |  | X | X | X | X |  |
|  | *Trigonidium obtusum* Lindl. | Ep |  | X | X | X |  |  |
|  | *Trigonidium turbinatum* Rchb.f. | Ep |  | X | X |  |  |  |
|  | *Trizeuxis falcata* Lindl. | Ep |  |  | X | X | X | X |
|  | *Vanilla angustipetala* Schltr. | Hep |  |  | X |  |  |  |
|  | *Vanilla bahiana* Hoehne | Hep |  | X | X |  | X | X |
|  | *Vanilla chamissonis* Klotzsch | Hep |  |  | X |  | X | X |
|  | *Vanilla denticulata* Pabst | Hep |  | X | X |  |  |  |
|  | *Vanilla dubia* Hoehne | Hep | EN | X | X |  | X |  |
|  | *Vanilla dungsii* Pabst | Hep |  | X | X |  |  |  |
|  | *Vanilla edwallii* Hoehne | Hep |  | X | X |  | X |  |
|  | *Vanilla gardneri* Rolfe | Hep |  |  | X | X |  |  |
|  | *Vanilla organensis* Rolfe | Hep |  | X | X |  |  |  |
|  | *Vanilla palmarum* (Salzm. ex Lindl.) Lindl. | Hep |  |  | X | X | X | X |
|  | *Vanilla parvifolia* Barb.Rodr. | Hep |  | X | X |  |  |  |
|  | *Vanilla perexilis* Bertoni | Hep |  |  | X |  |  |  |
|  | *Warczewiczella candida* (Lindl.) Rchb.f. | Ep |  | X | X |  |  |  |
|  | *Warczewiczella wailesiana* (Lindl.) Rchb.f. ex Morren | Ep |  | X | X |  |  |  |
|  | *Warmingia eugenii* Rchb.f. | Ep |  |  | X |  | X |  |
|  | *Xylobium colleyi* (Batem. ex Lindl.) Rolfe | Ep |  |  | X | X |  |  |
|  | *Xylobium variegatum* (Ruiz & Pav.) Mansf. | Ep |  |  | X | X | X | X |
|  | *Zootrophion atropurpureum* (Lindl.) Luer | Ep |  | X | X |  |  |  |
|  | *Zygopetalum crinitum* Lodd. | Ep |  | X | X |  |  |  |
|  | *Zygopetalum maxillare* Lodd. | Ep |  |  | X |  | X |  |
|  | *Zygostates alleniana* Kraenzl. | Ep |  |  | X |  |  |  |
|  | *Zygostates bradei* (Schltr.) Garay | Ep |  | X | X |  |  |  |
|  | *Zygostates cornigera* (Cogn.) Toscano | Ep |  | X | X |  |  |  |
|  | *Zygostates cornuta* Lindl. | Ep |  | X | X |  |  |  |
|  | *Zygostates dazhyrhiza* (Kraenzl.) Schltr. | Ep |  |  | X |  |  |  |
|  | *Zygostates grandiflora* (Lindl.) Mansf. | Ep |  | X | X |  |  |  |
|  | *Zygostates kuhlmannii* Brade | Ep | EN | X | X |  |  |  |
|  | *Zygostates linearisepala* (Senghas) Toscano | Ep | CR | X | X |  |  |  |
|  | *Zygostates lunata* Lindl. | Ep |  | X | X |  |  |  |
|  | *Zygostates multiflora* (Rolfe) Schltr. | Ep |  | X | X |  |  |  |
|  | *Zygostates octavioreisii* Porto & Brade | Ep |  | X | X |  |  |  |
|  | *Zygostates ovatipetala* (Brade) Toscano | Ep |  | X | X |  |  |  |
|  | *Zygostates pellucida* Rchb.f. | Ep |  | X | X |  |  |  |
|  | *Zygostates pustulata* (Kraenzl.) Schltr. | Ep |  | X | X |  |  |  |
| **Piperaceae** | *Peperomia alata* Ruiz & Pav. | Ep |  |  | X | X | X | X |
|  | *Peperomia argyreia* (Miq.) E.Morr. | Ep |  | X | X |  |  |  |
|  | *Peperomia armondii* Yunck. | Ep |  | X | X |  |  |  |
|  | *Peperomia campinasana* Yunck. | Ep |  | X | X |  | X |  |
|  | *Peperomia castelosensis* Yunck. | Ep |  | X | X |  |  |  |
|  | *Peperomia catharinae* Miq. | Ep |  |  | X |  |  |  |
|  | *Peperomia caulibarbis* Miq. | Ep |  | X | X |  |  |  |
|  | *Peperomia choroniana* C.DC. | Ep |  | X | X |  |  |  |
|  | *Peperomia circinnata* Link | Ep |  |  | X | X | X |  |
|  | *Peperomia clivicola* Yunck. | Ep |  | X | X |  |  |  |
|  | *Peperomia corcovadensis* Gardner | Ep |  |  | X |  |  |  |
|  | *Peperomia crinicaulis* C.DC. | Ep |  | X | X |  | X |  |
|  | *Peperomia delicatula* Henschen | Ep |  |  | X | X |  |  |
|  | *Peperomia diaphanoides* Dahlst. | Ep |  | X | X |  |  |  |
|  | *Peperomia dichotoma* Regel | Ep |  | X | X |  |  |  |
|  | *Peperomia distachya* (L.) A.Dietr. | Ep |  |  | X |  |  |  |
|  | *Peperomia divaricata* Yunck. | Ep |  |  | X | X |  |  |
|  | *Peperomia elongata* Kunth | Ep |  |  | X | X | X |  |
|  | *Peperomia emarginella* (Sw.) C.DC. | Ep |  |  | X |  |  |  |
|  | *Peperomia galioides* H.B.K. | Ep |  |  | X |  | X |  |
|  | *Peperomia glabella* (Sw.) A.Dietr. | Ep |  |  | X | X |  |  |
|  | *Peperomia glazioui* C.DC. | Ep |  | X | X |  |  |  |
|  | *Peperomia gracilis* Dahlst. | Ep | EN | X | X |  |  |  |
|  | *Peperomia guarujana* C.DC. | Ep | CR | X | X |  |  |  |
|  | *Peperomia hernandiifolia* (Vahl) A.Dietr. | Ep |  |  | X | X |  |  |
|  | *Peperomia hilariana* Miq. | Ep |  | X | X |  |  |  |
|  | *Peperomia ibiramana* Yunck. | Ep |  | X | X |  |  |  |
|  | *Peperomia loxensis* Kunth | Ep |  |  | X |  | X |  |
|  | *Peperomia macrostachya* (Vahl) A.Dietr. | Ep |  |  | X | X |  |  |
|  | *Peperomia magnoliifolia* (Jacq.) A.Dietr. | Ep |  |  | X | X |  |  |
|  | *Peperomia mandioccana* Miq. | Ep |  | X | X |  |  |  |
|  | *Peperomia martiana* Miq. | Ep |  |  | X |  |  |  |
|  | *Peperomia megapotamica* Dahlst. | Ep |  | X | X |  |  |  |
|  | *Peperomia minensis* Henschen | Ep |  | X | X |  |  |  |
|  | *Peperomia nitida* Dahlst. | Ep |  | X | X |  | X | X |
|  | *Peperomia obtusifolia* (L.) A.Dietr. | Ep |  |  | X | X | X | X |
|  | *Peperomia pereirae* Yunck. | Ep |  | X | X |  |  |  |
|  | *Peperomia pereskiifolia* (Jacq.) Kunth | Ep |  |  | X | X | X |  |
|  | *Peperomia pernambucensis* Miq. | Ep |  | X | X |  |  |  |
|  | *Peperomia pilicaulis* C.DC. | Ep |  |  | X | X |  |  |
|  | *Peperomia pseudobcordata* Yunck. | Ep |  | X | X |  |  |  |
|  | *Peperomia pseudoestrellensis* C.DC. | Ep |  | X | X |  |  |  |
|  | *Peperomia psilostachya* C.DC. | Ep |  |  | X | X | X |  |
|  | *Peperomia quadrifolia* (L.) Kunth | Ep |  |  | X |  | X |  |
|  | *Peperomia rhombea* Ruiz & Pav. | Ep |  |  | X | X |  |  |
|  | *Peperomia rizzinii* Yunck. | Ep |  | X | X |  |  |  |
|  | *Peperomia rotundifolia* (L.) Kunth | Ep |  |  | X | X |  |  |
|  | *Peperomia rubricaulis* (Nees) A.Dietr. | Ep |  | X | X |  | X |  |
|  | *Peperomia rufispica* Yunck. | Ep | CR | X | X |  |  |  |
|  | *Peperomia serpens* (Sw.) Loud. | Ep |  |  | X | X |  |  |
|  | *Peperomia stroemfeltii* Dahlst. | Ep |  | X | X |  |  |  |
|  | *Peperomia subemarginata* Yunck. | Ep |  | X | X |  |  |  |
|  | *Peperomia suboppositifolia* Yunck. | Ep | EN | X | X |  |  |  |
|  | *Peperomia subpilosa* Yunck. | Ep |  | X | X |  |  |  |
|  | *Peperomia subretusa* Yunck. | Ep |  | X | X |  |  |  |
|  | *Peperomia subternifolia* Yunck. | Ep |  | X | X |  |  |  |
|  | *Peperomia tenella* (Sw.) A.Dietr. | Ep |  |  | X | X | X |  |
|  | *Peperomia tetraphylla* (G.Forst.) Hook. & Arn. | Ep |  |  | X |  | X | X |
|  | *Peperomia trinervis* Ruiz & Pav. | Ep |  |  | X |  |  |  |
|  | *Peperomia trineura* Miq. | Ep |  |  | X |  | X | X |
|  | *Peperomia trineuroides* Dahlst. | Ep |  | X | X |  |  |  |
|  | *Peperomia turbinata* Dahlst. | Ep |  | X | X |  |  |  |
|  | *Peperomia urocarpa* Fisch. & C.A.Mey. | Ep |  | X | X | X | X |  |
|  | *Peperomia velloziana* Miq. | Ep |  | X | X |  | X | X |
| **Polypodiaceae** | *Alansmia alfaroi* (Donn. Sm.) Moguel & M.Kessler | Ep |  | X | X |  |  |  |
|  | *Alansmia reclinata* (Brack.) Moguel & M.Kessler | Ep |  | X | X |  |  |  |
|  | *Alansmia senilis* (Fée) Moguel & M.Kessler | Ep | CR |  | X | X |  |  |
|  | *Campyloneurum acrocarpon* Fée | Ep |  |  | X |  |  |  |
|  | *Campyloneurum aglaolepis* (Alston) de la Sota | Ep |  |  | X |  |  |  |
|  | *Campyloneurum austrobrasilianum* (Alston) de la Sota | Ep |  |  | X |  |  |  |
|  | *Campyloneurum centrobrasilianum* Lellinger | Ep |  | X | X |  | X |  |
|  | *Campyloneurum decurrens* (Raddi) C.Presl | Ep |  | X | X |  |  |  |
|  | *Campyloneurum fallax* Fée | Ep |  | X | X |  |  |  |
|  | *Campyloneurum minus* Fée | Ep |  | X | X |  |  |  |
|  | *Campyloneurum nitidum* (Kaulf.) C.Presl | Ep |  |  | X |  |  |  |
|  | *Campyloneurum rigidum* J.Sm. | Ep |  |  | X |  |  |  |
|  | *Campyloneurum vulpinum* (Lindm.) Ching | Ep |  |  | X |  |  |  |
|  | *Ceradenia albidula* (Baker) L.E.Bishop | Ep |  | X | X |  |  |  |
|  | *Ceradenia capillaris* (Desv.) L.E.Bishop | Ep | VU |  | X | X |  |  |
|  | *Ceradenia glaziovii* (Baker) Labiak | Ep | EN | X | X |  |  |  |
|  | *Ceradenia itatiaiensis* Labiak & Condack | Ep |  | X | X |  |  |  |
|  | *Ceradenia spixiana* (Mart. ex Mett.) L.E.Bishop | Ep |  |  | X |  |  |  |
|  | *Cochlidium pumilum* C.Chr. | Ep |  |  | X | X | X | X |
|  | *Cochlidium punctatum* (Raddi) L.E.Bishop | Ep |  | X | X |  |  |  |
|  | *Cochlidium serrulatum* (Sw.) L.E.Bishop | Ep |  |  | X | X | X | X |
|  | *Grammitis fluminensis* Fée | Ep | EN | X | X |  |  |  |
|  | *Grammitis leptopoda* (C.H.Wright) Copel. | Ep |  |  | X |  |  |  |
|  | *Lellingeria apiculata* (Kunze ex Klotzsch) A.R.Sm. & R.C.Moran | Ep |  |  | X |  |  |  |
|  | *Lellingeria brasiliensis* (Rosenst.) Labiak | Ep | VU | X | X |  |  |  |
|  | *Lellingeria brevistipes* (Mett. ex Kuhn) A.R.Sm. & R.C.Moran | Ep |  | X | X |  |  |  |
|  | *Lellingeria depressa* (C.Chr.) A.R.Sm. & R.C.Moran | Ep |  | X | X |  |  |  |
|  | *Lellingeria suspensa* (L.) A.R.Sm. & R.C.Moran | Ep |  |  | X | X |  |  |
|  | *Leucotrichum organense* (Gardner) Labiak | Ep |  | X | X |  |  |  |
|  | *Leucotrichum schenckii* (Hieron.) Labiak | Ep |  | X | X |  |  |  |
|  | *Melpomene albicans* Lenhert | Ep |  |  | X |  |  |  |
|  | *Melpomene flabelliformis* (Poir.) A.R.Sm. & R.C.Moran | Ep |  |  | X |  |  |  |
|  | *Melpomene melanostica* (Kunze) A.R.Sm. & R.C.Moran | Ep |  |  | X | X |  |  |
|  | *Melpomene pilosissima* (M.Martens & Galeotti) A.R.Sm. & R.C.Moran | Ep |  |  | X |  |  |  |
|  | *Microgramma crispata* (Fée) R.M.Tryon & A.F.Tryon | Ep |  | X | X |  |  |  |
|  | *Microgramma geminata* (Schrad.) A.F.Tryon & R.M.Tryon | Ep |  | X | X |  |  |  |
|  | *Microgramma lindbergii* (Kuhn) de la Sota | Ep |  |  | X |  | X |  |
|  | *Microgramma lycopodioides* (L.) Copel. | Ep |  |  | X | X |  |  |
|  | *Microgramma microsoroides* Salino et al. | Ep |  | X | X |  |  |  |
|  | *Microgramma percussa* (Cav.) de la Sota | Ep |  |  | X | X | X | X |
|  | *Microgramma persicariifolia* (Schrad.) C.Presl | Ep |  |  | X | X | X |  |
|  | *Microgramma squamulosa* (Kaulf.) de la Sota | Ep |  |  | X |  | X |  |
|  | *Microgramma tecta* (Kaulf.) Alston | Ep |  |  | X | X | X |  |
|  | *Microgramma vaccinniifolia* (Langsd. & Fisch.) Copel. | Ep |  |  | X |  | X |  |
|  | *Moranopteris achilleifolia* (Kaulf.) R.Y.Hirai & J.Prado | Ep |  |  | X |  |  |  |
|  | *Moranopteris gradata* (Baker) R.Y.Hirai & J.Prado | Ep |  | X | X |  |  |  |
|  | *Moranopteris perpusilla* (Maxon) R.Y.Hirai & J.Prado | Ep |  | X | X |  |  |  |
|  | *Moranopteris setosa* (Kaulf.) R.Y.Hirai & J.Prado | Ep |  | X | X |  |  |  |
|  | *Moranopteris x bradei* (Labiak & F.B.Matos) R.Y.Hirai & J.Prado | Ep |  | X | X |  |  |  |
|  | *Niphidium crassifolium* (L.) Lellinger | Ep |  |  | X | X | X |  |
|  | *Niphidium rufosquamatum* Lellinger | Ep |  |  | X |  |  |  |
|  | *Pecluma camptophyllaria* (Fée) M.G.Price | Ep |  |  | X |  |  |  |
|  | *Pecluma chnoophora* (Kunze) Salino & Assis | Ep |  |  | X |  |  |  |
|  | *Pecluma dispersa* (A.M. Evans) M.G.Price | Ep |  |  | X |  |  |  |
|  | *Pecluma filicula* (Kaulf) M.G.Price | Ep |  |  | X |  |  |  |
|  | *Pecluma imbeana* (Brade) Salino | Ep | CR | X | X |  |  |  |
|  | *Pecluma pectinatiformis* (Lindm.) M.G.Price | Ep |  |  | X |  |  |  |
|  | *Pecluma pilosa* (A.M. Evans) M.Kessler & A.R.Sm. | Ep |  |  | X |  |  |  |
|  | *Pecluma plumula* (Willd.) M.G.Price | Ep |  |  | X |  |  |  |
|  | *Pecluma recurvata* (Kaulf.) M.G.Price | Ep |  |  | X |  |  |  |
|  | *Pecluma sicca* (Lindm.) M.G.Price | Ep |  |  | X |  |  |  |
|  | *Pecluma singeri* (de la Sota) M.G.Price | Ep |  |  | X |  |  |  |
|  | *Pecluma truncorum* (Lindm.) M.G.Price | Ep |  |  | X |  |  |  |
|  | *Phlebodium aureum* (L.) J.Sm. | Ep |  |  | X | X | X |  |
|  | *Phlebodium decumanum* (Willd.) J.Sm. | Ep |  |  | X | X | X |  |
|  | *Phlebodium pseudoaureum* (Cav.) Lellinger | Ep |  |  | X | X | X |  |
|  | *Pleopeltis astrolepis* (Liebm.) E.Fourn. | Ep |  |  | X |  |  |  |
|  | *Pleopeltis furcata* (L.) A.R.Sm. | Ep |  |  | X |  |  |  |
|  | *Pleopeltis hirsutissima* (Raddi) de la Sota | Ep |  |  | X |  | X |  |
|  | *Pleopeltis lepidopteris* (Langsd. & Fisch.) de la Sota | Ep |  | X | X |  |  |  |
|  | *Pleopeltis macrocarpa* (Willd.) Kaulf. | Ep |  |  | X | X | X |  |
|  | *Pleopeltis minima* (Bory) J.Prado & R.Y.Hirai | Ep |  |  | X |  |  |  |
|  | *Pleopeltis monoides* (Weath.) Salino | Ep | EN | X | X |  |  |  |
|  | *Pleopeltis pleopeltidis* (Fée) de la Sota | Ep |  |  | X |  |  |  |
|  | *Pleopeltis pleopeltifolia* (Raddi) Alston | Ep |  | X | X |  | X |  |
|  | *Pleopeltis polypodioides* (L.) E.G.Andrews & Windham | Ep |  |  | X | X | X |  |
|  | *Polypodium dulce* Poir. | Ep |  |  | X |  |  |  |
|  | *Serpocaulon adnatum* (Kunze ex Klotzsch) A.R.Sm. | Ep |  |  | X | X |  |  |
|  | *Serpocaulon catharinae* (Langsd. & Fisch.) A.R.Sm. | Ep |  |  | X |  |  |  |
|  | *Serpocaulon fraxinifolium* (Jacq.) A.R.Sm. | Ep |  |  | X | X | X |  |
|  | *Serpocaulon glandulosissimum* (Brade) Labiak & J.Prado | Ep |  | X | X |  |  |  |
|  | *Serpocaulon hirsutulum* (T.Moore) Schwartsb. & A.R.Sm. | Ep |  | X | X |  |  |  |
|  | *Serpocaulon laetum* (C.Presl) Schwartsb. & A.R.Sm. | Ep |  | X | X |  |  |  |
|  | *Serpocaulon latipes* (Langsd. & Fisch.) A.R.Sm. | Ep |  |  | X |  |  |  |
|  | *Serpocaulon levigatum* (Cav.) A.R.Sm. | Ep |  |  | X |  |  |  |
|  | *Serpocaulon meniscifolium* (Langsd. & Fisch.) A.R.Sm. | Ep |  | X | X |  |  |  |
|  | *Serpocaulon mexiae* (Copel.) A.R.Sm. | Ep |  | X | X |  | X |  |
|  | *Serpocaulon triseriale* (Sw.) A.R.Sm. | Ep |  |  | X | X | X | X |
|  | *Serpocaulon x pubescens* (Rosenst.) Schwartsb. & A.R.Sm. | Ep |  | X | X |  |  |  |
|  | *Stenogrammitis limula* (Christ) Labiak | Ep | CR |  | X |  |  |  |
|  | *Stenogrammitis pumila* (Labiak) Labiak | Ep | CR | X | X |  |  |  |
|  | *Stenogrammitis wittigiana* (Fée & Glaz.) Labiak | Ep |  | X | X |  |  |  |
|  | *Terpsichore asplenifolia* (L.) A.R.Sm. | Ep |  |  | X | X |  |  |
|  | *Terpsichore chrysleri* (Copel.) A.R.Sm. | Ep |  |  | X |  |  |  |
|  | *Terpsichore semihirsuta* (Klotzsch) A.R.Sm. | Ep | EN |  | X |  |  |  |
|  | *Terpsichore taxifolia* (L.) A.R.Sm. | Ep | EN |  | X | X |  |  |
|  | *Zygophlebia longipilosa* (C.Chr.) L.E.Bishop | Ep |  | X | X |  |  |  |
| **Psilotaceae** | *Psilotum nudum* (L.) P.Beauv. | Ep |  |  | X | X | X |  |
| **Pteridaceae** | *Ananthacorus angustifolius* (Sw.) Underw. & Maxon | Ep |  |  | X | X | X |  |
|  | *Anetium citrifolium* (L.) Splitg. | Ep |  |  | X | X |  |  |
|  | *Hecistopteris pumila* (Spreng.) J.Sm. | Ep |  |  | X | X | X |  |
|  | *Polytaenium cajenense* (Desv.) Benedict | Ep |  |  | X | X |  |  |
|  | *Polytaenium feei* (W. Schaffn.) Maxon | Ep |  |  | X |  |  |  |
|  | *Polytaenium guayanense* (Hieron.) Alston | Ep |  |  | X | X |  |  |
|  | *Polytaenium lineatum* (Sw.) J.Sm. | Ep |  |  | X |  |  |  |
|  | *Radiovittaria gardneriana* (Fée) E.H.Crane | Ep |  |  | X |  |  |  |
|  | *Radiovittaria stipitata* (Kunze) E.H.Crane | Ep |  |  | X | X |  |  |
|  | *Vittaria graminifolia* Kaulf. | Ep |  |  | X | X | X |  |
|  | *Vittaria lineata* (L.) Sm. | Ep |  |  | X | X | X |  |
|  | *Vittaria scabrida* Klotzsch | Ep |  |  | X |  |  |  |
| **Rubiaceae** | *Hillia illustris* (Vell.) K.Schum. | Ep |  |  | X | X |  |  |
|  | *Hillia parasitica* Jacq. | Ep |  | X | X | X |  |  |
|  | *Hillia saldanhaei* K.Schum. | Ep |  | X | X |  |  |  |
|  | *Hillia ulei* K.Krause | Ep |  |  | X | X |  |  |
|  | *Notopleura bahiensis* C.M.Taylor | Ep |  | X | X |  |  |  |
|  | *Schradera polycephala* DC. | Hep |  |  | X | X |  |  |
| **Solanaceae** | *Dyssochroma longipes* (Sendtn.) Miers | Hep |  | X | X |  |  |  |
|  | *Dyssochroma viridiflorum* (Sims) Miers | Hep |  | X | X |  |  |  |
|  | *Markea atlantica* Stehmann & Giacomin | Hep |  | X | X |  |  |  |
|  | *Solandra grandiflora* Sw. | Ep |  |  | X |  |  |  |
| **Urticaceae** | *Boehmeria caudata* Sw. | Ep |  |  | X |  | X |  |
|  | *Boehmeria cylindrica* (L.) Sw. | Ep |  |  | X |  | X |  |
|  | *Coussapoa floccosa* Akkermans & C.C.Berg | Hep |  | X | X |  |  |  |
|  | *Coussapoa microcarpa* (Schott) Rizzini | Hep |  | X | X |  | X |  |
|  | *Coussapoa pachyphylla* Akkermans & C.C.Berg | Hep |  | X | X |  |  |  |
